# Supplementary material for: Timing and ecological priority shaped the diversification of sedges in the Himalayas
Source: PeerJ. 2019 Jun 7;7:e6792. doi: 10.7717/peerj.6792 (PMC6557248; doi:10.7717/peerj.6792)
Supplement: Figure S1 — Constraint trees (A, B, C, D and E) represent node which is constrained for identifying nature of Himalayan Carex lineages in each clade. Here every shape represents all the given species in that particular clade [(a) Vignea clade: 240 species, (b) core Unispicate clade: 136 species, (c) core Carex clade: 575 species, (d) three clades combined: 940 species, (e) Kobresia clade: 42 species, in all outgroup included] and each constrained topology showed monophyletic Himalayan lineages. [file peerj-07-6792-s001.pdf]

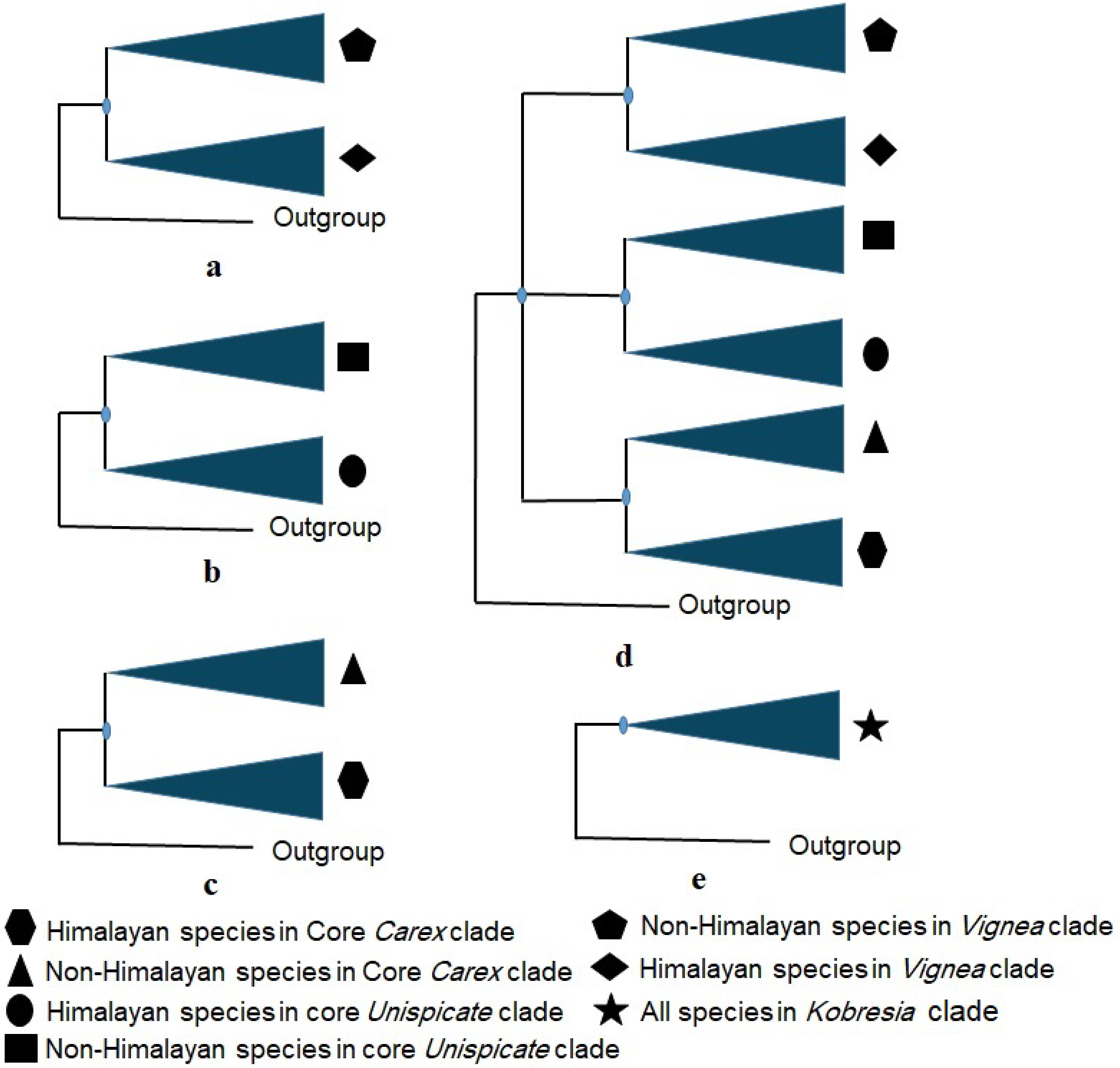
Outgroup
a
Outgroup
b
Outgroup
c
Outgroup
d
Outgroup
e
Himalayan species in Core *Carex* clade
Non-Himalayan species in Core *Carex* clade
Himalayan species in core *Unispicate* clade
Non-Himalayan species in core *Unispicate* clade
Non-Himalayan species in *Vignea* clade
Himalayan species in *Vignea* clade
All species in *Kobresia* clade
