# Supplementary figures and images for: Timing and ecological priority shaped the diversification of sedges in the Himalayas
Source: PeerJ. 2019 Jun 7;7:e6792. doi: 10.7717/peerj.6792 (PMC6557248; doi:10.7717/peerj.6792)

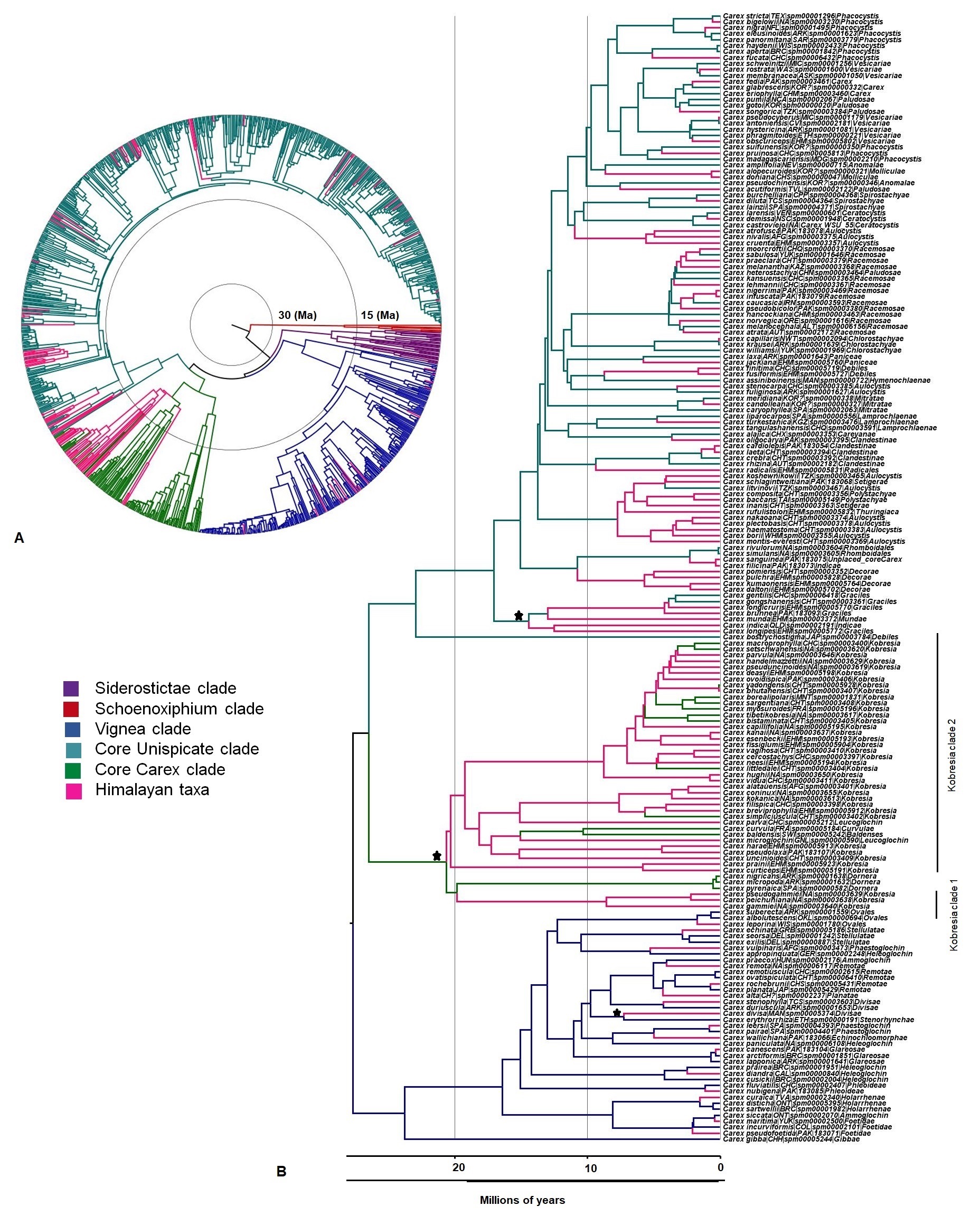

Supplement: Figure S2 — Circular dated phylogeny (A) presented 966 Carex species from treePL analysis showing divergence of Himalayan taxa in tribe Cariceae. (B) Rectangular tree is pruned represented 300 taxa, here each colored band denoated major clades while black colored branches in three clades (Vignea, core Unispicate and core Carex) represent Himalayan lineages. Star showed divergence for the Himalayan taxa in three major clades (Vignea, core Unispicate, core Carex) [file peerj-07-6792-s002.jpg]

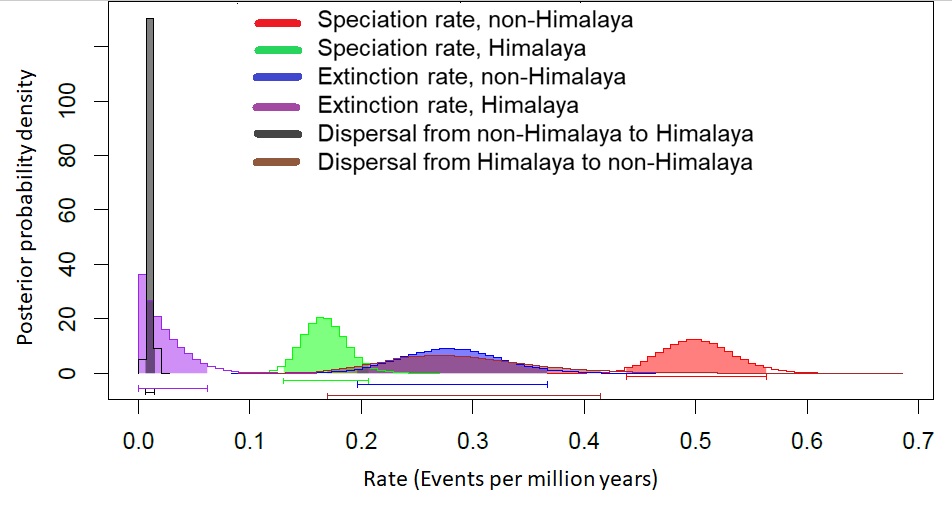

Supplement: Figure S3 — Rate of speciation, extinction, and dispersal for Himalayan and non-Himalayan lineages, estimated under the model Geographic State Speciation and Extinction (GeoSSE). Here, rates are in events per million years. [file peerj-07-6792-s003.jpg]

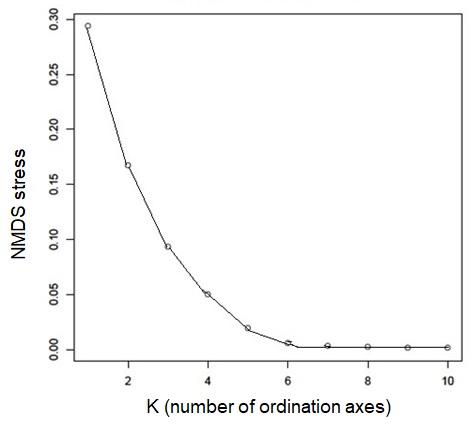

Supplement: Figure S4 — The ordination in a set of initial non-metric multidimensional scaling (NMDS) ordinations including data for both the tip states and ancestral reconstructions in order to get best ordination [file peerj-07-6792-s004.jpg]

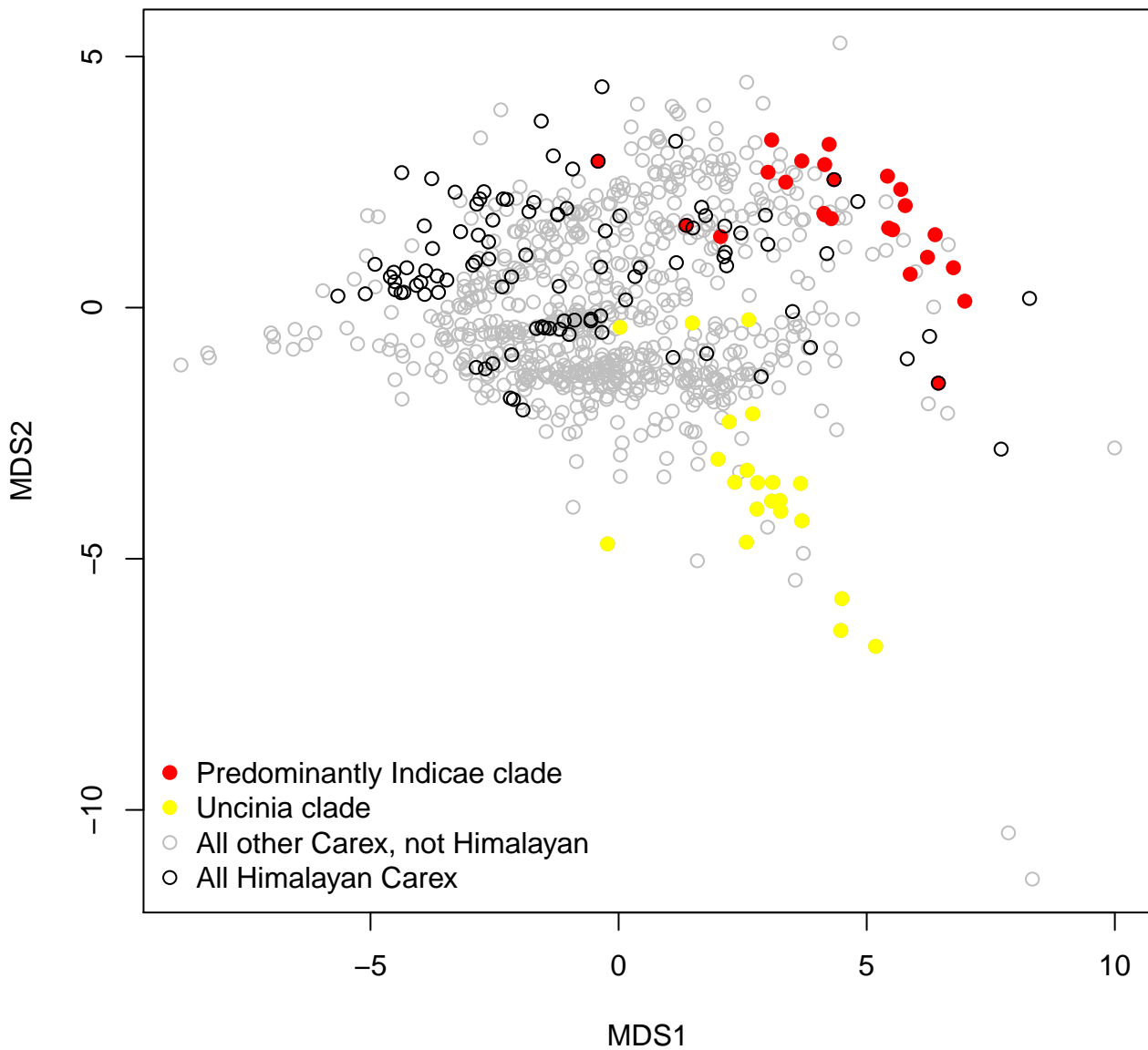

Supplement: Figure S5 — Ordinations showed Himalayan taxa overall are widespread in climatic niche and present no consistent trend [file peerj-07-6792-s005.pdf]

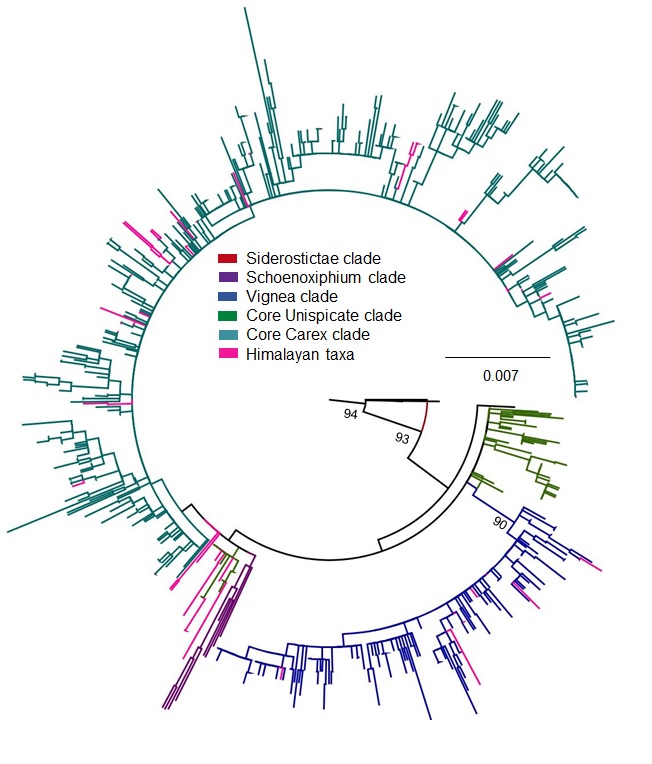

Supplement: File S2 — Here, color scheme is used for each major clade: Siderostictae (red), Schoenoxiphium (purple), Vignea (blue), core Unispicate (green) and core Carex (aqua), Himalayan taxa (pink) for differentiation and understanding, while outgroup shows in black color. Nodes of major clades show bootstrap values. [file peerj-07-6792-s015.jpg]

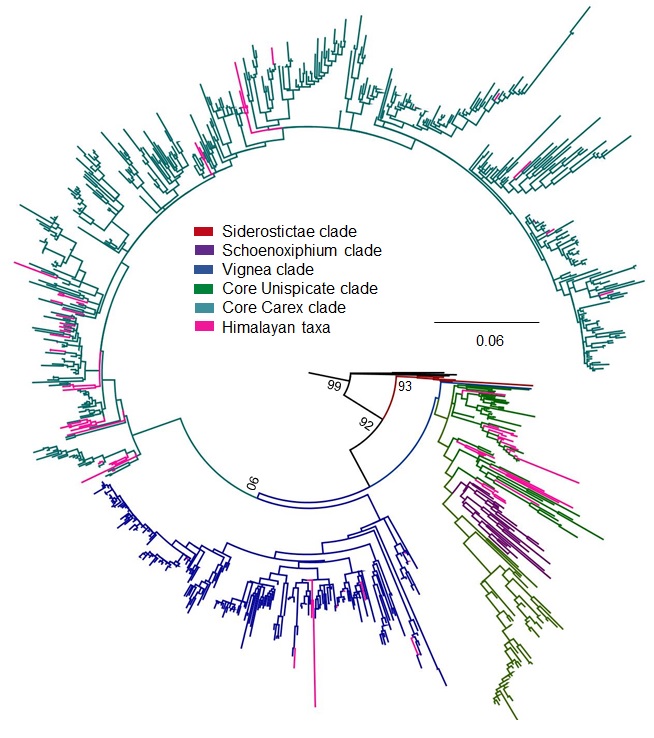

Supplement: File S4 — Here, color scheme is used for each major clade: Siderostictae (red), Schoenoxiphium (purple), Vignea (blue), core Unispicate (green) and core Carex (aqua), Himalayan taxa (pink) for differentiation and understanding, while outgroup shows in black color. Nodes of major clades show bootstrap values. [file peerj-07-6792-s017.jpg]

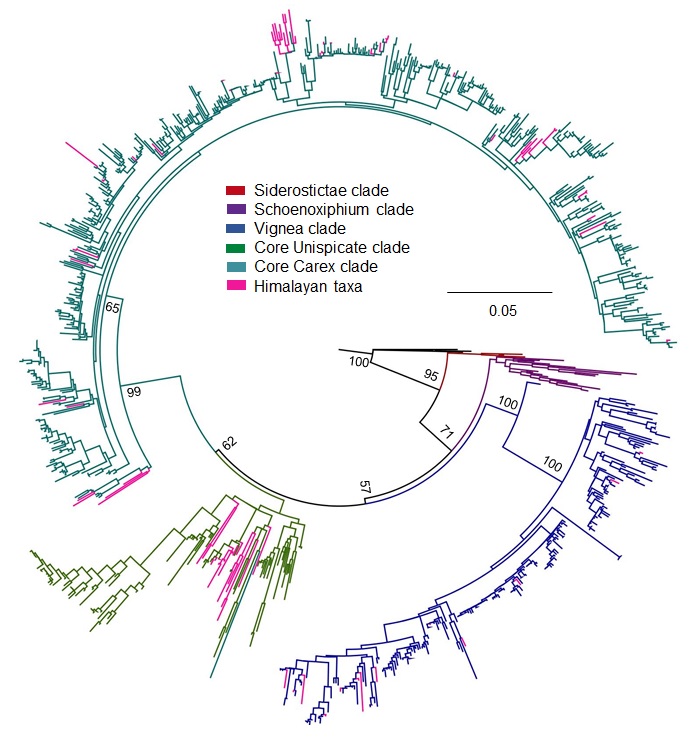

Supplement: File S6 — Here, color scheme is used for each major clade: Siderostictae (red), Schoenoxiphium (purple), Vignea (blue), core Unispicate (green) and core Carex (aqua), Himalayan taxa (pink) for differentiation and understanding, while outgroup shows in black color. Nodes of major clades show bootstrap values. [file peerj-07-6792-s019.jpg]

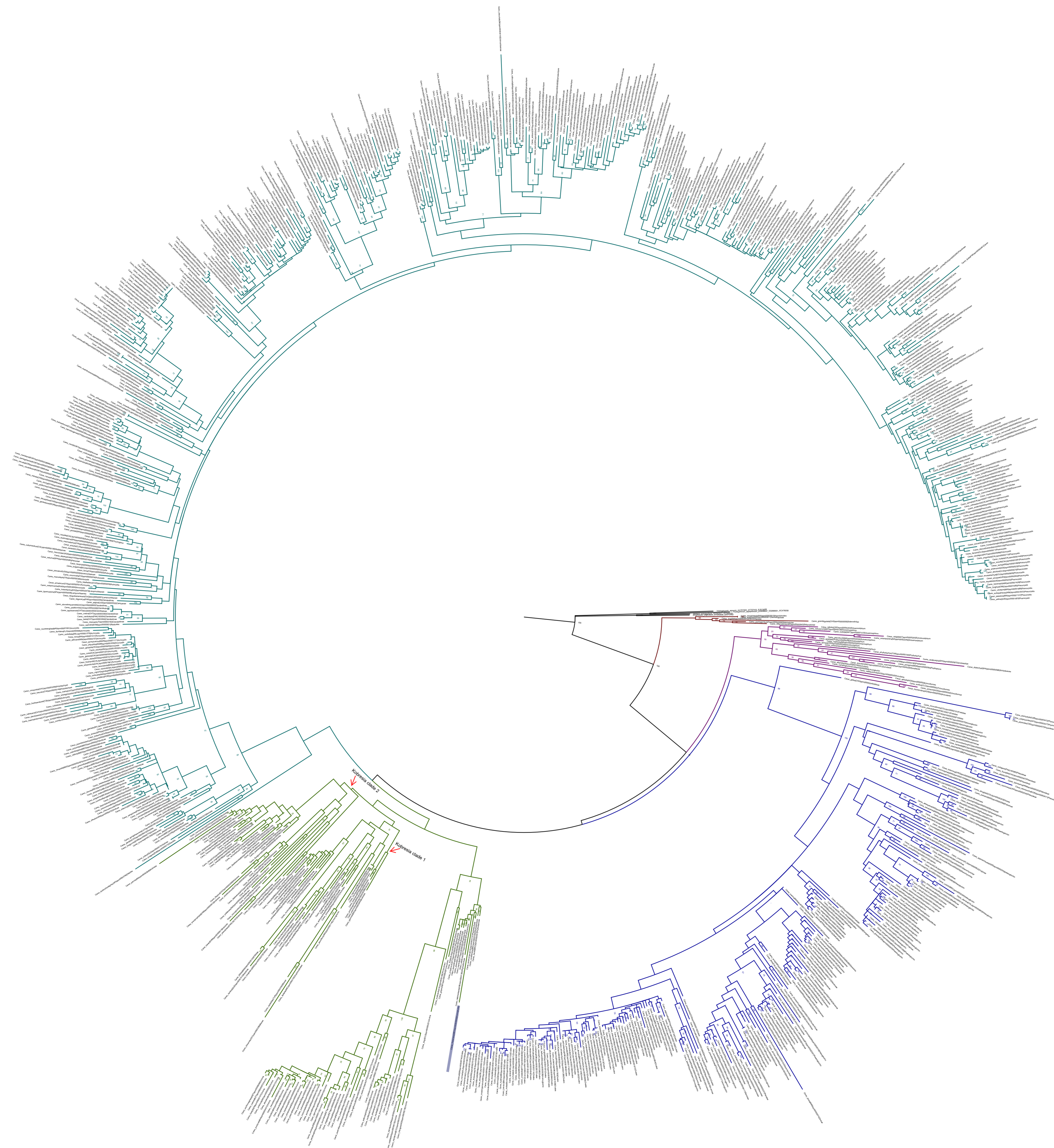

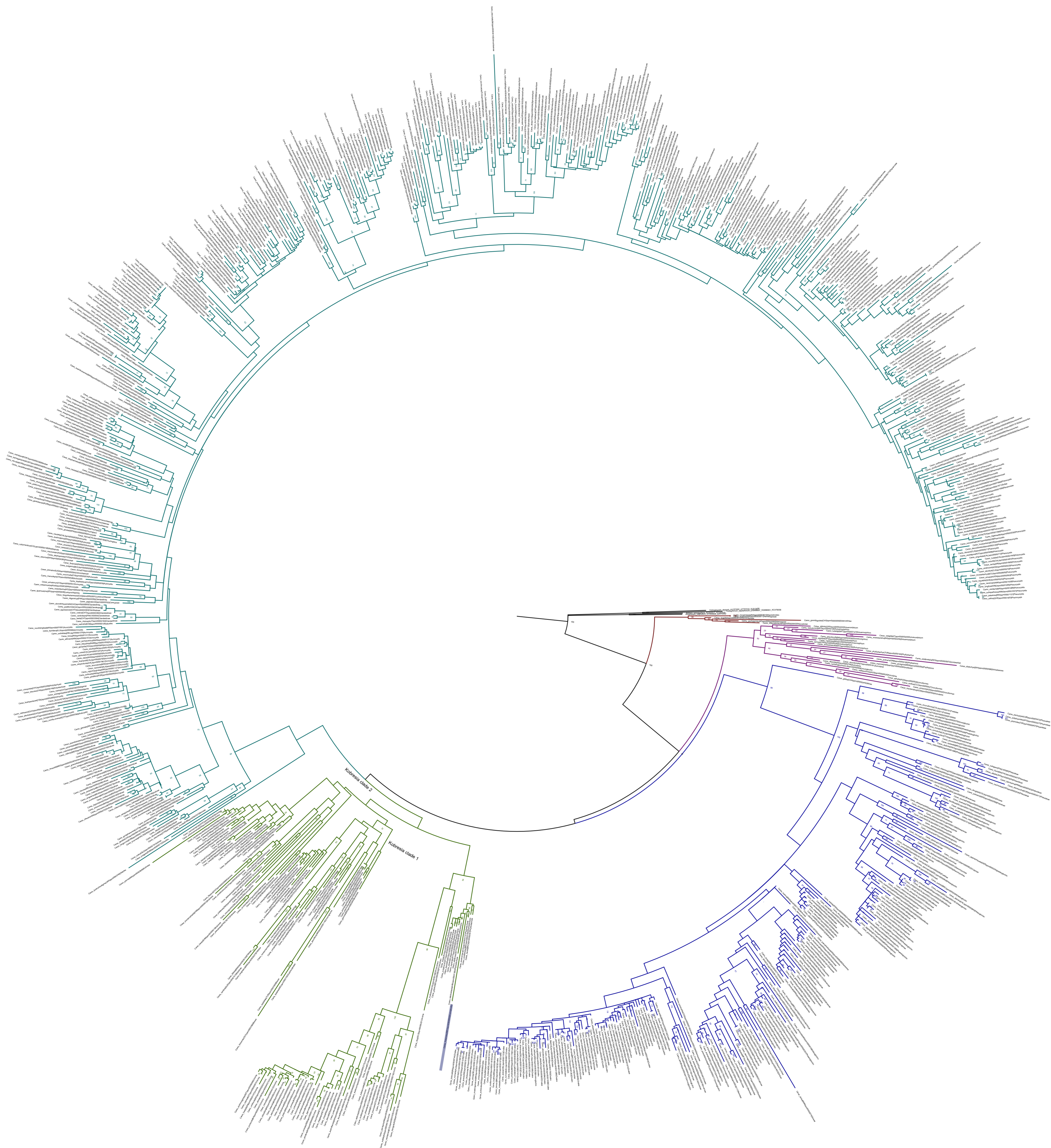

Supplement: File S8 — Every tip labels as species—country of collection—unique specimen number/accession number—traditional section. Country of specimen’s collection is coded according to three-letter TDWG geographical code. Here, color scheme is used for each major clade: Siderostictae (red), Schoenoxiphium (purple), Vignea (blue), core Unispicate (green) and core Carex (aqua) for differentiation and understanding, while outgroup shows in black color. Here two Kobresia clades: Kobresia clade 1 and Kobresia clade 2, are also mentioned [file peerj-07-6792-s021.pdf]

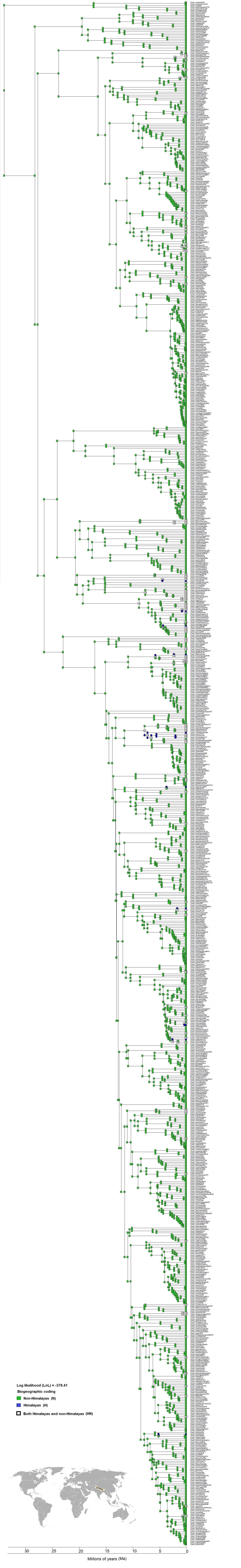

Supplement: File S9 — Here tip shows extant taxa with biogeographical region denoted as (H) if present in Himalayan, (N) if in non-Himalayan and (NH) if widely distributed to both Himalayan and non-Himalayan regions. All internal nodes represent ancestral range as: Himalayan (H), non-Himalayan (N) and both Himalayan and non-Himalayan (NH). The map represent the Himalayas region, while other than Himalayas is coded under non-Himalayas region. [file peerj-07-6792-s022.jpg]

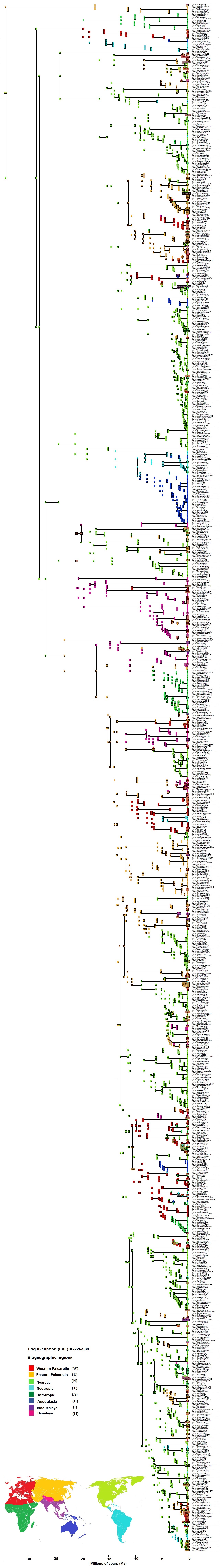

Supplement: File S10 — Here tip shows extant taxa with biogeographical ranges and all internal nodes represent ancestral range. The world map denotes all the eight ranges which are: W, Western Palearctic, E, Eastern Palearctic, N, Nearctic, A, Afrotropic, T, Neotropic, U = Australasia, I, Indo-Malaya, H, Himalaya. Each geographical range designates with different color code, indicates on the map. [file peerj-07-6792-s023.jpg]
