# Supplementary material for: Timing and ecological priority shaped the diversification of sedges in the Himalayas
Source: PeerJ. 2019 Jun 7;7:e6792. doi: 10.7717/peerj.6792 (PMC6557248; doi:10.7717/peerj.6792)
Supplement: Table S1A — *MA, Morton Arboretum, Lisle, USA *PMNH, Pakistan Museum of Natural History, Islamabad, Pakistan [file peerj-07-6792-s006.docx]

**Table S1a** Sampling information of specimens collected in this study

| **Species** | ***MA**  **Voucher** | ***PMNH**  **voucher** | **Sample collecting ID** | **Elevation**  **(meter)** | **Locality** | **Range** | **Sampling month & year** |
| --- | --- | --- | --- | --- | --- | --- | --- |
| *Carex atrofusca* | 183078 | 42672 | KJB-01 | 4724 | Khunjerab, Gilgit-Baltistan | Karakoram | Sept, 2015 |
| *Carex atrofusca* | 183077 | 42677 | KJB-05 | 4724 | Khunjerab, Gilgit-Baltistan | Karakoram | Sept, 2015 |
| *Carex brunnea* | 183095 | 42664 | BGH-02 | 1446 | Bagh, Kashmir | Himalaya | Sept, 2015 |
| *Carex brunnea* | 183093 | 42665 | BGH-04 | 1446 | Bagh, Kashmir | Himalaya | Sept., 2015 |
| *Carex brunnea* | 183087 | 42657 | HRG-01 | 1719 | Harighal, Kashmir | Himalaya | Sept.,2015 |
| *Carex brunnea* | 183089 | 42682 | MUR-02 | 1782 | Ghora gali, Murree | Himalaya | August, 2015 |
| *Carex brunnea* | 183090 | 42695 | RWT-09 | 1775 | Banjosa Lake, Rawalakot | Himalaya | July, 2015 |
| *Carex brunnea* | 183056 | 42660 | RWT-11 | 1722 | Piona, Kashmir | Himalaya | Sept.,2015 |
| *Carex brunnea* | 183094 | 42655 | RWT-12 | 1797 | Banjosa Lake, Rawalakot | Himalaya | Sept.,2015 |
| *Carex brunnea* | 183088 | 42661 | RWT-14 | 1722 | Piona, Kashmir | Himalaya | Sept.,2015 |
| *Carex brunnea* | 183096 | 42662 | RWT-15 | 1722 | Piona, Kashmir | Himalaya | Sept.,2015 |
| *Carex brunnea* | 183092 | 42658 | RWT-19 | 1722 | Piona, Kashmir | Himalaya | Sept.,2015 |
| *Carex brunnea* | 183091 | 42656 | RWT-20 | 1722 | Piona, Kashmir | Himalaya | Sept.,2015 |
| *Carex canescens* | 183104 | 42671 | FMS-05 | 3342 | Fairy Meadows, Gilgit-Baltistan | Himalaya | Sept., 2015 |
| *Carex canescens* | 183103 | 42674 | FMS-08 | 3312 | Fairy Meadows, Gilgit-Baltistan | Himalaya | Sept., 2015 |
| *Carex cardiolepis* | 183054 | 42694 | SAD-07 | 2500 | Miandam Swat | Himalaya | July, 2012 |
| *Carex filicina* | 183074 | 42663 | BGH-01 | 1446 | Bagh, Kashmir | Himalaya | Sept., 2015 |
| *Carex filicina* | 183073 | 42696 | RWT-08 | 1775 | Banjosa Lake, Rawalakot | Himalaya | July, 2015 |
| *Carex filicina* | 183065 | 42659 | RWT-17 | 1797 | Banjosa Lake, Rawalakot | Himalaya | Sept., 2015 |
| *Carex fedia* | 183064 | xxx | SAD-01 | 1200 | Matta Swat | Himalaya | April, 2014 |
| *Carex infuscata* | 183057 | 42675 | FMS-06 | 3342 | Fairy Meadows, Gilgit-Baltistan | Himalaya | Sept., 2015 |
| *Carex infuscata* | 183079 | 42673 | FMS-07 | 3312 | Fairy Meadows, Gilgit-Baltistan | Himalaya | Sept., 2015 |
| *Carex infuscata* | 183080 | 42693 | SAD-05 | 2543 | Naran | Himalaya | July, 2011 |
| *Carex infuscata* | 183058 | 42692 | SAD-10 | 2543 | Saiful Malook, Naran | Himalaya | July, 2011 |
| *Carex nubigena* | 183082 | 42687 | KSH-07 | 2380 | Arang Kel, Kashmir | Himalaya | June, 2015 |
| *Carex nubigena* | 183085 | 42667 | TLP-01 | 2600 | Tolipeer, Kashmir | Himalaya | Sept., 2015 |
| *Carex nubigena* | 183086 | 42669 | TLP-03 | 2600 | Tolipeer, Kashmir | Himalaya | Sept., 2015 |
| *Carex nubigena* | 183081 | 42668 | TLP-04 | 2600 | Tolipeer, Kashmir | Himalaya | Sept., 2015 |
| *Carex nubigena* | 183084 | 42666 | TLP-05 | 2600 | Tolipeer, Kashmir | Himalaya | Sept., 2015 |
| *Carex nubigena* | 183083 | 42670 | TLP-06 | 2600 | Tolipeer, Kashmir | Himalaya | Sept., 2015 |
| *Carex pamirica* | 183076 | 42691 | SAD-06 | 2,226 | Deosai, Skardu | Himalaya | June, 2011 |
| *Carex pseudofoetida* | 183071 | 42678 | KJB-04 | 4724 | Khunjerab, Gilgit-Baltistan | Karakoram | Sept., 2015 |
| *Carex pseudofoetida* | 183072 | 42676 | KJB-06 | 4724 | Khunjerab, Gilgit-Baltistan | Karakoram | Sept., 2015 |
| *Carex pseudolaxa* | 183109 | 42653 | KGN-01 | 2543 | Saif ul maluk, Naran | Himalaya | Sept.,2015 |
| *Carex pseudolaxa* | 183107 | 42654 | KGN-02 | 2543 | Saif ul maluk, Naran | Himalaya | Sept.,2015 |
| *Carex pseudolaxa* | 183106 | 42688 | KSH-01 | 2273 | Taobat, Kashmir | Himalaya | June, 2015 |
| *Carex pseudolaxa* | 183108 | 42689 | KSH-04 | 2263 | Taobat, Kashmir | Himalaya | June, 2015 |
| *Carex pseudolaxa* | 183112 | 42679 | RKP-01 | 2295 | Rakaposhi, Gilgit-Baltistan | Karakoram | Sept., 2015 |
| *Carex pseudolaxa* | 183111 | 42681 | RKP-04 | 2295 | Rakaposhi, Gilgit-Baltistan | Karakoram | Sept., 2015 |
| *Carex pseudolaxa* | 183110 | 42680 | RKP-05 | 2295 | Rakaposhi, Gilgit-Baltistan | Karakoram | Sept., 2015 |
| *Carex pseudolaxa* | 183105 | 42690 | SAD-04 | 2,226 | Deosai, Skardu | Himalaya | June,2011 |
| *Carex psychrophila* | 183055 | 42685 | SAD-12 | 2400 | Miandam Swat | Himalaya | May, 2013 |
| *Carex sanguinea* | 183075 | 42686 | SAD-08 | 2000 | Miandam Swat | Himalaya | May, 2011 |
| *Carex schlagintweitiana* | 183068 | 42683 | SAD-11 | 2500 | Malamjaba Swat | Himalaya | April, 2011 |
| *Carex wallichiana* | 183066 | xxx | RWT-03 | 1775 | Banjosa Lake, Rawalakot | Himalaya | July, 2015 |
| *Carex wallichiana* | 183067 | 42684 | SAD-14 | 2400 | Miandam Swat | Himalaya | May, 2013 |
| *Carex dimorpholepis* | 184272 | xxx | 8 | 2400 | Miandam Swat | Himalaya | May, 2013 |
| *Carex brunnea* | 184278 | xxx | D-2 | 2,500 | Dunga Gali, Galyat | Himalaya | April, 2016 |
| *Carex diandra* | 184275 | xxx | 4 | 2400 | Miandam Swat | Himalaya | June, 2015 |
| *Carex schlagintweitiana* | 184274; 184273 | xxx | Shogran-8 | 2,362 | Shogran, Kaghan | Himalaya | April, 2016 |
| *Carex cardiolepis* | 184271 | xxx | D-4 | 2,500 | Dunga Gali, Galyat | Himalaya | April, 2016 |
| *Carex pseudocyperus* | 184276 | xxx | 9 | 2,226 | Near Barapani bridge Deosai | Himalaya | June, 2011 |
| *Carex pamirica* | 184270 | xxx | 7 | 2,226 | Deosai, Skardu | Himalaya | June, 2011 |
| *Carex simpliciuscula* | 183069 | xxx | KJB-02 | 4724 | Khunjerab, Gilgit-Baltistan | Karakoram | Sept., 2015 |
| *Carex simpliciuscula* | 183070 | xxx | KJB-03 | 4724 | Khunjerab, Gilgit-Baltistan | Karakoram | Sept., 2015 |

*MA = Morton Arboretum, Lisle, USA

*PMNH= Pakistan Museum of Natural History, Islamabad, Pakistan
