# Supplementary material for: Timing and ecological priority shaped the diversification of sedges in the Himalayas
Source: PeerJ. 2019 Jun 7;7:e6792. doi: 10.7717/peerj.6792 (PMC6557248; doi:10.7717/peerj.6792)
Supplement: Table S1B — *Herbaria: MO: Missouri Botanical Garden Herbarium, MSB: Botanishche Staatssammiung München, E: Royal Botanic Garden Edinburgh [file peerj-07-6792-s007.docx]

**Table S1b** Herbarium vouchers’ information for the species used in this study

| **Species** | **Herbarium vouchers** | **Herbaria*** | **Locality** | **Ranges** |
| --- | --- | --- | --- | --- |
| *Carex breviprophylla* | E00184752 | E | Nepal: Mustang: Lower Jhuwa Khola N Samar | Himalaya |
| *Carex daltonii* | E00047630 | E | India: Sikkim: North District: Bop, between Chungthang and Maltin | Himalaya |
| *Carex finitima* | E00269484 | E | China: Yunnan: Gaoligong Shan Region: Lushui Xian, Luzhang Zheng. Area above Yaojiaping forest station, Sanjiang River, E side of Gaoligong Shan | Hengduan Mountain |
| *Carex fissiglumis* | E00693544 | E | India: Sikkim: West District: Choktsering Chhu valley, between Bikbari and Chaunrikhiang | Himalaya |
| *Carex fucata* | 5778634 | MO | China: Yunnan: Gongshan: Cikai Zheng: E side of Gaoligong Shan, in the vicinity of the Daxue Cao waterfall, along the Danzhu He on the road from the Nu Jiang at Danzhu to the Myanmar border. | Hengduan Mountain |
| *Carex fusiformis* | E00666305 | E | Bhutan: W side of Ura La, SE of Byakar | Himalaya |
| *Carex gentilis* | 5306057 | MO | China: Yunnan: Nujiang Lisu Aut. Pref, Gongshan Co., Gongshan Suburbs, Salween River Banks | Hengduan Mountain |
| *Carex harae* | E00048401 | E | India: Sikkim: North District: Yumthang, Lachung Chhu | Himalaya |
| *Carex harae* | E00048402 | E | India: Sikkim: North District: Phune, Lachung Chhu | Himalaya |
| *Carex jackiana* | E00666333 | E | NE Nepal: Kathmandu: East slope of Tinjure Danda ridge between Door Pani and Chauki. | Himalaya |
| *Carex kumaonensis* | E00656734 | E | Nepal: Western Development Region: Gandaki Zone: Manang District: Dharapani to Tal | Himalaya |
| *Carex longicruris* | E00693600 | E | Nepal: Ila, Bheri River | Himalaya |
| *Carex longicruris* | E00693633 | E | Bhutan: Chukka District: Between Bunakha and Chimakothi | Himalaya |
| *Carex longipes* | E00693601 | E | NE Nepal: Kathmandu: Between Chauki and Tinjure | Himalaya |
| *Carex longipes* | E00003298 | E | China: Yunnan: Lijiang Prefecture: Xin Zhu forest, between Judian and Litiping Plateau | Hengduan Mountain |
| *Carex obovatosquamata* | E00666339 | E | China: Yunnan: Diqing Prefecture, Deqin County: W side of Deqin Valley | Hengduan Mountain |
| *Carex obscuriceps* | E00693599 | E | Bhutan: 6 km N of Thimphu Dzong | Himalaya |
| *Carex ovatispiculata* | MSB-140867 | MSB | China: Xizang, S. Tibet: Tibetan Himalaya, Everest E, Kama Chu, opposite Makalu E. Glacier tongue | Tibetan Himalaya |
| *Carex prainii* | E00256786 | E | Nepal: Eastern Development Region: Sagarmatha: Solu Khumbu: Namche Bazar. Along the trail to Phurte | Himalaya |
| *Carex pruinosa* | E00263657 | E | China: Yunnan: Gaoligong Shan Region: Tengchong Xian, Wutai Xiang. Km 24.7 along highway S 317, Xiaodifang village and vicinity. W side of Gaoligonig Shan. E facing 0-10° slope | Hengduan Mountains |
| *Carex pruinosa* | E00264126 | E | China: Yunnan: Gaoligong Shan Region: Tengchong Xian, Wutai Xiang. Km 24.7 along highway S 317, Xiaodifang village and vicinity. W side of Gaoligonig Shan. E facing 0-10° slope | Hengduan Mountains |
| *Carex pulchra* | E00693608 | E | NE Nepal: Simbua Khola, between Tseram & Tarangdi | Himalaya |
| *Carex radicalis* | E00693596 | E | Bhutan: Thimphu District: Hill above Hospital, Thimphu | Himalaya |
| *Carex rufulistolon* | E00424592 | E | Nepal: Central Nepal. Mustang. Annapurna Conservation area. Trekking route Jomosom-Ghorepani. Between Larjung and Kalopani vill. Kali Ghandaki river | Himalaya |
| *Carex rufulistolon* | E00270126 | E | Nepal: Eastern Development Region: Sagarmatha: Solu Khumbu: Namche Bazar. Hillside above Namche on the route Kenjoma | Himalaya |
| *Carex rufulistolon* | E00238910 | E | Nepal: Dhawalagiri Zone: Mustang Distr.: Between Chaile (Chele) and Kyuten, N. side of Ghyakar Khola | Himalaya |
| *Carex yadongensis* | E00177570 | E | Tibet: Changthang, Upper Targo Tsangpo basin | Tibetan Himalaya |

*Herbaria:

MO: Missouri Botanical Garden Herbarium

MSB: Botanishche Staatssammiung München

E: Royal Botanic Garden Edinburgh
