# Supplementary material for: Timing and ecological priority shaped the diversification of sedges in the Himalayas
Source: PeerJ. 2019 Jun 7;7:e6792. doi: 10.7717/peerj.6792 (PMC6557248; doi:10.7717/peerj.6792)
Supplement: Table S2 — All the species included in the study with original taxa labelling as available on dryad dataset (http://dx.doi.org/10.5061/dryad.k05qb), while new sequences labelled as species name —region (three-letter TDWG geographical code) —herbarium voucher number. [file peerj-07-6792-s008.docx]

**Table S2 The presence of species in Himalayan, non-Himalayan and both regions and binary coding for narrow and broad sense analyses are mentioned.** All the species included in the study with original taxa labelling as available on dryad dataset (<http://dx.doi.org/10.5061/dryad.k05qb>), while new sequences labelled as species name | region (three-letter TDWG geographical code) | herbarium voucher number.

| **Original labelling of Taxa** | **Regions** | **Narrow Sense** | | **Broad Sense** | | |  |
| --- | --- | --- | --- | --- | --- | --- | --- |
|  |  | **H** | **N** | | **H** | **N** | |
| *Carex aboriginum\|STARR\|B1231\|IDA\|spm00001838* | Non-Himalayan | 0 | 1 | | 0 | 1 | |
| *Carex abrupta\|STARR\|B520\|BRC\|spm00001126* | Non-Himalayan | 0 | 1 | | 0 | 1 | |
| *Carex abscondita\|STARR\|B057\|MRY\|spm00000676* | Non-Himalayan | 0 | 1 | | 0 | 1 | |
| *Carex acaulis\|LUCENO\|NA\|CLS\|spm00000518* | Non-Himalayan | 0 | 1 | | 0 | 1 | |
| *Carex accrescens\|HIPP\|NA\|KOR\|spm00000018* | Non-Himalayan | 0 | 1 | | 0 | 1 | |
| *Carex acicularis\|STARR_FORD\|STA_85\|NZ?\|spm00005161* | Non-Himalayan | 0 | 1 | | 0 | 1 | |
| *Carex acidicola\|STARR\|B039\|ALA\|spm00000658* | Non-Himalayan | 0 | 1 | | 0 | 1 | |
| *Carex acuta\|LUCENO\|\|Italy\|spm00003084* | Non-Himalayan | 0 | 1 | | 0 | 1 | |
| *Carex acutiformis\|STARR\|B1516\|TVL\|spm00002122* | Both | 1 | 1 | | 1 | 1 | |
| *Carex adelostoma\|STARR\|B1012\|ARK\|spm00001619* | Non-Himalayan | 0 | 1 | | 0 | 1 | |
| *Carex adusta\|STARR\|B1466\|ABT\|spm00002072* | Non-Himalayan | 0 | 1 | | 0 | 1 | |
| *Carex aematorrhyncha\|LUCENO\|NA\|CLS\|spm00000532* | Non-Himalayan | 0 | 1 | | 0 | 1 | |
| *Carex aequialta\|SANGTAE\|E0001\|KOR?\|spm00000320* | Non-Himalayan | 0 | 1 | | 0 | 1 | |
| *Carex aestivalis\|STARR\|B062\|PEN\|spm00000681* | Non-Himalayan | 0 | 1 | | 0 | 1 | |
| *Carex aethiopica\|JIMENEZMEJIAS\|\|CPP\|spm00004365* | Non-Himalayan | 0 | 1 | | 0 | 1 | |
| *Carex aggregata\|STARR\|B066\|KTY\|spm00000685* | Non-Himalayan | 0 | 1 | | 0 | 1 | |
| *Carex agrostoides\|REZNICEK\|NA\|MXE\|spm00000263* | Non-Himalayan | 0 | 1 | | 0 | 1 | |
| *Carex alajica\|JIMENEZMEJIAS\|\|CHX\|spm00003353* | Non-Himalayan | 0 | 1 | | 0 | 1 | |
| *Carex alata\|STARR\|B068\|ALA\|spm00000687* | Non-Himalayan | 0 | 1 | | 0 | 1 | |
| *Carex alatauensis\|JIMENEZMEJIAS\|\|AFG\|spm00003401* | Both | 1 | 1 | | 1 | 1 | |
| *Carex alba\|LUCENO\|NA\|SPA\|spm00000584* | Non-Himalayan | 0 | 1 | | 0 | 1 | |
| *Carex albata\|STARR_FORD\|STA_773\|JAP\|spm00005254* | Non-Himalayan | 0 | 1 | | 0 | 1 | |
| *Carex albicans\|STARR_FORD\|STA_187\|ARK\|spm00005157* | Non-Himalayan | 0 | 1 | | 0 | 1 | |
| *Carex albida\|JIMENEZMEJIAS\|\|CAL\|spm00003602* | Non-Himalayan | 0 | 1 | | 0 | 1 | |
| *Carex albolutescens\|STARR\|B075\|OKL\|spm00000694* | Non-Himalayan | 0 | 1 | | 0 | 1 | |
| *Carex albonigra\|STARR\|B1014\|ARK\|spm00001621* | Non-Himalayan | 0 | 1 | | 0 | 1 | |
| *Carex albursina\|STARR\|B780\|KTY\|spm00001384* | Non-Himalayan | 0 | 1 | | 0 | 1 | |
| *Carex allanii\|K_FORD\|NA\|NZS\|spm00002185* | Non-Himalayan | 0 | 1 | | 0 | 1 | |
| *Carex alma\|STARR\|B085\|CAL\|spm00000703* | Non-Himalayan | 0 | 1 | | 0 | 1 | |
| *Carex alopecuroides\|SANGTAE\|D0055\|KOR?\|spm00000321* | Both | 1 | 1 | | 1 | 1 | |
| *Carex alta\|HIPP\|alta_0436063\|CH?\|spm00002237* | Both | 1 | 1 | | 1 | 1 | |
| *Carex amphibola\|STARR\|B035\|ALA\|spm00000654* | Non-Himalayan | 0 | 1 | | 0 | 1 | |
| *Carex amplifolia\|STARR\|B097\|NEV\|spm00000715* | Non-Himalayan | 0 | 1 | | 0 | 1 | |
| *Carex andersonii\|LUCENO\|NA\|AGS\|spm00000522* | Non-Himalayan | 0 | 1 | | 0 | 1 | |
| *Carex andina\|LUCENO\|NA\|CLC\|spm00000527* | Non-Himalayan | 0 | 1 | | 0 | 1 | |
| *Carex andringitrensis\|GEHRKE\|NA\|MDG\|spm00000169* | Non-Himalayan | 0 | 1 | | 0 | 1 | |
| *Carex angolensis\|GEHRKE\|NA\|MLW\|spm00000170* | Non-Himalayan | 0 | 1 | | 0 | 1 | |
| *Carex angustata\|STARR\|B095\|CAL\|spm00000713* | Non-Himalayan | 0 | 1 | | 0 | 1 | |
| *Carex angustilepis\|REZNICEK\|NA\|MXE\|spm00000356* | Non-Himalayan | 0 | 1 | | 0 | 1 | |
| *Carex angustispica\|REZNICEK\|NA\|MXS\|spm00000262* | Non-Himalayan | 0 | 1 | | 0 | 1 | |
| *Carex anisostachys\|REZNICEK\|NA\|MXT\|spm00000258* | Non-Himalayan | 0 | 1 | | 0 | 1 | |
| *Carex annectens\|STARR\|B112\|ALA\|spm00000730* | Non-Himalayan | 0 | 1 | | 0 | 1 | |
| *Carex anthoxanthea\|STARR\|B961\|WAS\|spm00001568* | Non-Himalayan | 0 | 1 | | 0 | 1 | |
| *Carex antoniensis\|MESTERHAZY\|NA\|CVI\|spm00002181* | Non-Himalayan | 0 | 1 | | 0 | 1 | |
| *Carex aperta\|STARR\|B1235\|BRC\|spm00001842* | Non-Himalayan | 0 | 1 | | 0 | 1 | |
| *Carex aphylla\|LUCENO\|NA\|AGS\|spm00000526* | Non-Himalayan | 0 | 1 | | 0 | 1 | |
| *Carex appalachica\|STARR\|B103\|MAS\|spm00000721* | Non-Himalayan | 0 | 1 | | 0 | 1 | |
| *Carex appendiculata\|LUCENO\|\|Russia\|spm00003267* | Non-Himalayan | 0 | 1 | | 0 | 1 | |
| *Carex appressa\|HIPP\|NA\|NSW\|spm00002189* | Non-Himalayan | 0 | 1 | | 0 | 1 | |
| *Carex appropinquata\|HIPP\|appropinquata_3358\|GER\|spm00002248* | Non-Himalayan | 0 | 1 | | 0 | 1 | |
| *Carex aquatilis\|STARR_FORD\|\|QUE\|spm00005232* | Non-Himalayan | 0 | 1 | | 0 | 1 | |
| *Carex arapahoensis\|STARR\|B1118\|COL\|spm00001725* | Non-Himalayan | 0 | 1 | | 0 | 1 | |
| *Carex arcta\|STARR\|B092\|ASK\|spm00000710* | Non-Himalayan | 0 | 1 | | 0 | 1 | |
| *Carex arctata\|STARR\|B113\|WIS\|spm00000731* | Non-Himalayan | 0 | 1 | | 0 | 1 | |
| *Carex arctiformis\|STARR\|B1244\|BRC\|spm00001851* | Non-Himalayan | 0 | 1 | | 0 | 1 | |
| *Carex arenaria\|STARR\|B115\|DEL\|spm00000733* | Non-Himalayan | 0 | 1 | | 0 | 1 | |
| *Carex argyrantha\|STARR\|B116\|MRY\|spm00000734* | Non-Himalayan | 0 | 1 | | 0 | 1 | |
| *Carex arkansana\|STARR\|B119\|ILL\|spm00000737* | Non-Himalayan | 0 | 1 | | 0 | 1 | |
| *Carex arnellii\|LUCENO\|3E4MMR13\|SWE\|spm00003783* | Non-Himalayan | 0 | 1 | | 0 | 1 | |
| *Carex assiniboinensis\|STARR\|B104\|MAN\|spm00000722* | Non-Himalayan | 0 | 1 | | 0 | 1 | |
| *Carex astricta\|JIMENEZMEJIAS\|\|\|spm00004420* | Non-Himalayan | 0 | 1 | | 0 | 1 | |
| *Carex asturica\|JIMENEZMEJIAS\|\|SPA\|spm00004385* | Non-Himalayan | 0 | 1 | | 0 | 1 | |
| *Carex asynchrona\|REZNICEK\|NA\|MXE\|spm00000257* | Non-Himalayan | 0 | 1 | | 0 | 1 | |
| *Carex atherodes\|STARR\|B1242\|BRC\|spm00001849* | Non-Himalayan | 0 | 1 | | 0 | 1 | |
| *Carex athrostachya\|STARR\|B109\|CAL\|spm00000727* | Non-Himalayan | 0 | 1 | | 0 | 1 | |
| *Carex atlantica\|STARR_FORD\|\|PEN\|spm00005432* | Non-Himalayan | 0 | 1 | | 0 | 1 | |
| *Carex atractodes\|REZNICEK\|NA\|MXE\|spm00000256* | Non-Himalayan | 0 | 1 | | 0 | 1 | |
| *Carex atrata\|MESTERHAZY\|NA\|AUT\|spm00002172* | Both | 1 | 1 | | 1 | 1 | |
| *Carex atratiformis\|STARR\|B1144\|ABT\|spm00001751* | Non-Himalayan | 0 | 1 | | 0 | 1 | |
| *Carex atrofusca\|PAK\|183078* | Both | 1 | 1 | | 1 | 1 | |
| *Carex atropicta\|LUCENO\|NA\|AGS\|spm00000519* | Non-Himalayan | 0 | 1 | | 0 | 1 | |
| *Carex atrosquama\|STARR\|B1113\|MNT\|spm00001720* | Non-Himalayan | 0 | 1 | | 0 | 1 | |
| *Carex aucklandica\|JIMENEZMEJIAS\|\|\|spm00004410* | Non-Himalayan | 0 | 1 | | 0 | 1 | |
| *Carex augustinowiczii\|SANGTAE\|D0521\|KOR?\|spm00000322* | Non-Himalayan | 0 | 1 | | 0 | 1 | |
| *Carex aurea\|STARR\|B149\|\|spm00000767* | Non-Himalayan | 0 | 1 | | 0 | 1 | |
| *Carex aureolensis\|STARR\|B152\|MSI\|spm00000770* | Non-Himalayan | 0 | 1 | | 0 | 1 | |
| *Carex auriculata\|SANGTAE\|D0527\|KOR?\|spm00000345* | Non-Himalayan | 0 | 1 | | 0 | 1 | |
| *Carex austrina\|STARR\|B172\|MRY\|spm00000790* | Non-Himalayan | 0 | 1 | | 0 | 1 | |
| *Carex austroalpina\|LUCENO\|NA\|SPA\|spm00000535* | Non-Himalayan | 0 | 1 | | 0 | 1 | |
| *Carex austrocaroliniana\|STARR\|B1122\|NCA\|spm00001729* | Non-Himalayan | 0 | 1 | | 0 | 1 | |
| *Carex austrocompacta\|STARR_FORD\|STA_4\|TAS\|spm00005217* | Non-Himalayan | 0 | 1 | | 0 | 1 | |
| *Carex austroflaccida\|STARR_FORD\|STA_21\|\|spm00005091* | Non-Himalayan | 0 | 1 | | 0 | 1 | |
| *Carex austromexicana\|REZNICEK\|NA\|MXS\|spm00000358* | Non-Himalayan | 0 | 1 | | 0 | 1 | |
| *Carex aztecica\|REZNICEK\|NA\|MXS\|spm00000254* | Non-Himalayan | 0 | 1 | | 0 | 1 | |
| *Carex azuayae\|JIMENEZMEJIAS\|NA\|VEN\|spm00000593* | Non-Himalayan | 0 | 1 | | 0 | 1 | |
| *Carex baccans\|STARR_FORD\|STA_208\|TAI\|spm00005149* | Both | 1 | 1 | | 1 | 1 | |
| *Carex backii\|STARR\|B1400\|BRC\|spm00002007* | Non-Himalayan | 0 | 1 | | 0 | 1 | |
| *Carex baileyi\|STARR\|B133\|KTY\|spm00000751* | Non-Himalayan | 0 | 1 | | 0 | 1 | |
| *Carex baimaensis\|XIAOFENG\|NA\|CHS\|spm00000027* | Non-Himalayan | 0 | 1 | | 0 | 1 | |
| *Carex baldensis\|STARR_FORD\|\|SWI\|spm00005242* | Non-Himalayan | 0 | 1 | | 0 | 1 | |
| *Carex balfourii\|LUCENO\|NA\|REU\|spm00000528* | Non-Himalayan | 0 | 1 | | 0 | 1 | |
| *Carex baltzellii\|STARR\|B1307\|FLA\|spm00001914* | Non-Himalayan | 0 | 1 | | 0 | 1 | |
| *Carex banksiana\|STARR_FORD\|STA_68\|NZ?\|spm00005085* | Non-Himalayan | 0 | 1 | | 0 | 1 | |
| *Carex banksii\|LUCENO\|NA\|CLS\|spm00000545* | Non-Himalayan | 0 | 1 | | 0 | 1 | |
| *Carex barbarae\|STARR\|B846\|CAL\|spm00001449* | Non-Himalayan | 0 | 1 | | 0 | 1 | |
| *Carex barrattii\|STARR\|B130\|ALA\|spm00000748* | Non-Himalayan | 0 | 1 | | 0 | 1 | |
| *Carex basiantha\|STARR\|B017\|SCA\|spm00000637* | Non-Himalayan | 0 | 1 | | 0 | 1 | |
| *Carex basutorum\|GEHRKE\|NA\|NAT\|spm00000146* | Non-Himalayan | 0 | 1 | | 0 | 1 | |
| *Carex bathiei\|HIPP\|NA\|MDG\|spm00002195* | Non-Himalayan | 0 | 1 | | 0 | 1 | |
| *Carex bebbii\|STARR\|B127\|MAN\|spm00000745* | Non-Himalayan | 0 | 1 | | 0 | 1 | |
| *Carex bella\|STARR\|B1114\|COL\|spm00001721* | Non-Himalayan | 0 | 1 | | 0 | 1 | |
| *Carex bequaertii\|GEHRKE\|NA\|KEN\|spm00000176* | Non-Himalayan | 0 | 1 | | 0 | 1 | |
| *Carex bhutanensis\|JIMENEZMEJIAS\|\|CHT\|spm00003407* | Both | 1 | 1 | | 1 | 0 | |
| *Carex bicknellii\|STARR\|B126\|INI\|spm00000744* | Non-Himalayan | 0 | 1 | | 0 | 1 | |
| *Carex bicolor\|STARR\|B1015\|ARK\|spm00001622* | Non-Himalayan | 0 | 1 | | 0 | 1 | |
| *Carex bigelowii\|LUCENO\|\|\|spm00003230* | Both | 1 | 1 | | 1 | 1 | |
| *Carex bijiangensis\|JIMENEZMEJIAS\|\|CHT\|spm00003354* | Non-Himalayan | 0 | 1 | | 1 | 0 | |
| *Carex biltmoreana\|STARR\|B1462\|NCA\|spm00002068* | Non-Himalayan | 0 | 1 | | 0 | 1 | |
| *Carex binervis\|JIMENEZMEJIAS\|\|FRA\|spm00004357* | Non-Himalayan | 0 | 1 | | 0 | 1 | |
| *Carex bistaminata\|JIMENEZMEJIAS\|\|CHT\|spm00003405* | Non-Himalayan | 0 | 1 | | 1 | 1 | |
| *Carex blakei\|JIMENEZMEJIAS\|\|NSW\|spm00004358* | Non-Himalayan | 0 | 1 | | 0 | 1 | |
| *Carex blanda\|STARR\|B1212\|TEN\|spm00001819* | Non-Himalayan | 0 | 1 | | 0 | 1 | |
| *Carex blepharicarpa\|HIPP\|NA\|KOR\|spm00000012* | Non-Himalayan | 0 | 1 | | 0 | 1 | |
| *Carex bohemica\|HIPP\|bohemica_8650\|FIN\|spm00002276* | Non-Himalayan | 0 | 1 | | 0 | 1 | |
| *Carex bolanderi\|STARR\|B811\|CAL\|spm00001414* | Non-Himalayan | 0 | 1 | | 0 | 1 | |
| *Carex boliviensis\|REZNICEK\|NA\|MXC\|spm00000255* | Non-Himalayan | 0 | 1 | | 0 | 1 | |
| *Carex bonanzensis\|STARR\|B1213\|ARK\|spm00001820* | Non-Himalayan | 0 | 1 | | 0 | 1 | |
| *Carex bonariensis\|STARR_FORD\|STA_783\|BOL\|spm00005361* | Non-Himalayan | 0 | 1 | | 0 | 1 | |
| *Carex borbonica\|JIMENEZMEJIAS\|\|REU\|spm00004353* | Non-Himalayan | 0 | 1 | | 0 | 1 | |
| *Carex borealipolaris\|STARR\|B1224\|MNT\|spm00001831* | Non-Himalayan | 0 | 1 | | 0 | 1 | |
| *Carex borii\|JIMENEZMEJIAS\|\|WHM\|spm00003355* | Both | 1 | 1 | | 1 | 0 | |
| *Carex boryana\|JIMENEZMEJIAS\|\|REU\|spm00004377* | Non-Himalayan | 0 | 1 | | 0 | 1 | |
| *Carex bostrychostigma\|LUCENO\|4E4MMR13\|JAP\|spm00003784* | Non-Himalayan | 0 | 1 | | 0 | 1 | |
| *Carex brachycalama\|REZNICEK\|NA\|BOL\|spm00000368* | Non-Himalayan | 0 | 1 | | 0 | 1 | |
| *Carex brainerdii\|STARR\|B142\|CAL\|spm00000760* | Non-Himalayan | 0 | 1 | | 0 | 1 | |
| *Carex brasiliensis\|REZNICEK\|NA\|BZS\|spm00000366* | Non-Himalayan | 0 | 1 | | 0 | 1 | |
| *Carex breedlovei\|REZNICEK\|NA\|MXT\|spm00000249* | Non-Himalayan | 0 | 1 | | 0 | 1 | |
| *Carex breviaristata\|XIAOFENG\|NA\|CHS\|spm00000029* | Non-Himalayan | 0 | 1 | | 0 | 1 | |
| *Carex brevicaulis\|STARR_FORD\|STA_249\|TDC\|spm00005216* | Non-Himalayan | 0 | 1 | | 0 | 1 | |
| *Carex brevicollis\|JIMENEZMEJIAS\|NA\|TCS\|spm00003478* | Non-Himalayan | 0 | 1 | | 0 | 1 | |
| *Carex brevior\|STARR\|B1216\|BRC\|spm00001823* | Non-Himalayan | 0 | 1 | | 0 | 1 | |
| *Carex breviprophylla\|EHM\|E00184752* | Both | 1 | 1 | | 1 | 0 | |
| *Carex breweri\|STARR\|B969\|WAS\|spm00001576* | Non-Himalayan | 0 | 1 | | 0 | 1 | |
| *Carex brizoides\|STARR_FORD\|STA_784\|GER\|spm00005239* | Non-Himalayan | 0 | 1 | | 0 | 1 | |
| *Carex bromoides\|HIPP\|bromoides_3157\|WIS\|spm00002284* | Non-Himalayan | 0 | 1 | | 0 | 1 | |
| *Carex brunnea\|PAK\|183093* | Both | 1 | 1 | | 1 | 1 | |
| *Carex brunnescens\|WATERWAY\|\|\|spm00005976* | Non-Himalayan | 0 | 1 | | 0 | 1 | |
| *Carex brysonii\|STARR\|B036\|ALA\|spm00000655* | Non-Himalayan | 0 | 1 | | 0 | 1 | |
| *Carex buekii\|JIMENEZMEJIAS\|NA\|CZE\|spm00000558* | Non-Himalayan | 0 | 1 | | 0 | 1 | |
| *Carex bulbostylis\|STARR\|B029\|MSI\|spm00000648* | Non-Himalayan | 0 | 1 | | 0 | 1 | |
| *Carex bulgarica\|LUCENO\|NA\|BUL\|spm00000555* | Non-Himalayan | 0 | 1 | | 0 | 1 | |
| *Carex bullata\|STARR\|B157\|MAS\|spm00000775* | Non-Himalayan | 0 | 1 | | 0 | 1 | |
| *Carex burchelliana\|JIMENEZMEJIAS\|\|CPP\|spm00004368* | Non-Himalayan | 0 | 1 | | 0 | 1 | |
| *Carex bushii\|STARR\|B159\|DEL\|spm00000777* | Non-Himalayan | 0 | 1 | | 0 | 1 | |
| *Carex buxbaumii\|STARR\|B147\|ASK\|spm00000765* | Non-Himalayan | 0 | 1 | | 0 | 1 | |
| *Carex calcifugens\|STARR\|B049\|FLA\|spm00000668* | Non-Himalayan | 0 | 1 | | 0 | 1 | |
| *Carex calcis\|K_FORD\|NA\|NZS\|spm00002186* | Non-Himalayan | 0 | 1 | | 0 | 1 | |
| *Carex californica\|STARR\|B1078\|CAL\|spm00001685* | Non-Himalayan | 0 | 1 | | 0 | 1 | |
| *Carex caligena\|REZNICEK\|NA\|MXE\|spm00000264* | Non-Himalayan | 0 | 1 | | 0 | 1 | |
| *Carex camposii\|JIMENEZMEJIAS\|\|SPA\|spm00004363* | Non-Himalayan | 0 | 1 | | 0 | 1 | |
| *Carex camptoglochin\|LUCENO\|NA\|CLS\|spm00000589* | Non-Himalayan | 0 | 1 | | 0 | 1 | |
| *Carex canariensis\|HIPP\|canariensis_83917\|SPA\|spm00002291* | Non-Himalayan | 0 | 1 | | 0 | 1 | |
| *Carex candolleana\|SANGTAE\|E0109\|KOR?\|spm00000327* | Both | 1 | 1 | | 1 | 1 | |
| *Carex canescens\|PAK\|183104* | Both | 1 | 1 | | 1 | 1 | |
| *Carex capensis\|STARR_FORD\|STA_74\|\|spm00005202* | Non-Himalayan | 0 | 1 | | 0 | 1 | |
| *Carex capillaris\|STARR\|B1488\|NWT\|spm00002094* | Both | 1 | 1 | | 1 | 1 | |
| *Carex capillifolia\|STARR_FORD\|\|\|spm00005195* | Both | 1 | 1 | | 1 | 1 | |
| *Carex capitata\|STARR\|B1429\|KRA\|spm00002036* | Non-Himalayan | 0 | 1 | | 0 | 1 | |
| *Carex capitellata\|JIMENEZMEJIAS\|NA\|TUR\|spm00003459* | Non-Himalayan | 0 | 1 | | 0 | 1 | |
| *Carex cardiolepis\|PAK\|183054* | Both | 1 | 1 | | 1 | 0 | |
| *Carex careyana\|STARR\|B798\|KTY\|spm00001401* | Non-Himalayan | 0 | 1 | | 0 | 1 | |
| *Carex caroliniana\|STARR\|B197\|ALA\|spm00000815* | Non-Himalayan | 0 | 1 | | 0 | 1 | |
| *Carex caryophyllea\|STARR\|B1457\|SPA\|spm00002063* | Non-Himalayan | 0 | 1 | | 0 | 1 | |
| *Carex castanea\|STARR\|B876\|NFL\|spm00001479* | Non-Himalayan | 0 | 1 | | 0 | 1 | |
| *Carex castroviejoi\|*Carex_WSU_55*\|*FASTA.LABEL.NO.SPECIMEN.MATCH | Non-Himalayan | 0 | 1 | | 0 | 1 | |
| *Carex catharinensis\|JIMENEZMEJIAS\|\|BZS\|spm00004375* | Non-Himalayan | 0 | 1 | | 0 | 1 | |
| *Carex caucasica\|JIMENEZMEJIAS\|\|IRN\|spm00003593* | Non-Himalayan | 0 | 1 | | 0 | 1 | |
| *Carex caudata\|LUCENO\|NA\|CAL\|spm00000529* | Non-Himalayan | 0 | 1 | | 0 | 1 | |
| *Carex cephaloidea\|STARR\|B186\|MAI\|spm00000804* | Non-Himalayan | 0 | 1 | | 0 | 1 | |
| *Carex cephalophora\|STARR\|B210\|MRY\|spm00000828* | Non-Himalayan | 0 | 1 | | 0 | 1 | |
| *Carex cercostachys\|JIMENEZMEJIAS\|\|CHC\|spm00003397* | Both | 1 | 1 | | 1 | 0 | |
| *Carex chalciolepis\|STARR\|B175\|TAI\|spm00000793* | Non-Himalayan | 0 | 1 | | 0 | 1 | |
| *Carex chaofangii\|XIAOFENG\|NA\|CHS\|spm00000093* | Non-Himalayan | 0 | 1 | | 0 | 1 | |
| *Carex chapmanii\|STARR\|B214\|FLA\|spm00000832* | Non-Himalayan | 0 | 1 | | 0 | 1 | |
| *Carex cherokeensis\|STARR\|B212\|FLA\|spm00000830* | Non-Himalayan | 0 | 1 | | 0 | 1 | |
| *Carex chiapensis\|REZNICEK\|NA\|MXS\|spm00000267* | Non-Himalayan | 0 | 1 | | 0 | 1 | |
| *Carex chihuahuensis\|STARR\|B1205\|ARI\|spm00001812* | Non-Himalayan | 0 | 1 | | 0 | 1 | |
| *Carex chlorosaccus\|GEHRKE\|NA\|KEN\|spm00000180* | Non-Himalayan | 0 | 1 | | 0 | 1 | |
| *Carex chordalis\|REZNICEK\|NA\|MXC\|spm00000251* | Non-Himalayan | 0 | 1 | | 0 | 1 | |
| *Carex chordorrhiza\|STARR\|B1318\|BRC\|spm00001925* | Non-Himalayan | 0 | 1 | | 0 | 1 | |
| *Carex chungii\|XIAOFENG\|NA\|CHS\|spm00000022* | Non-Himalayan | 0 | 1 | | 0 | 1 | |
| *Carex circinata\|STARR\|B1370\|ARK\|spm00001977* | Non-Himalayan | 0 | 1 | | 0 | 1 | |
| *Carex clavata\|JIMENEZMEJIAS\|\|CPP\|spm00004348* | Non-Himalayan | 0 | 1 | | 0 | 1 | |
| *Carex cognata\|GEHRKE\|NA\|NAT\|spm00000182* | Non-Himalayan | 0 | 1 | | 0 | 1 | |
| *Carex colchica\|MESTERHAZY\|NA\|UKR\|spm00002173* | Non-Himalayan | 0 | 1 | | 0 | 1 | |
| *Carex collinsii\|STARR\|B209\|DEL\|spm00000827* | Non-Himalayan | 0 | 1 | | 0 | 1 | |
| *Carex collumanthus\|STARR_FORD\|STA_11\|COL\|spm00005156* | Non-Himalayan | 0 | 1 | | 0 | 1 | |
| *Carex comosa\|STARR\|B208\|CAL\|spm00000826* | Non-Himalayan | 0 | 1 | | 0 | 1 | |
| *Carex complanata\|STARR\|B164\|TEX\|spm00000782* | Non-Himalayan | 0 | 1 | | 0 | 1 | |
| *Carex complexa\|REZNICEK\|NA\|MXE\|spm00000357* | Non-Himalayan | 0 | 1 | | 0 | 1 | |
| *Carex composita\|JIMENEZMEJIAS\|\|CHT\|spm00003356* | Both | 1 | 1 | | 1 | 1 | |
| *Carex concinnoides\|STARR\|B1311\|CAL\|spm00001918* | Non-Himalayan | 0 | 1 | | 0 | 1 | |
| *Carex conferta\|GEHRKE\|NA\|ETH\|spm00000184* | Non-Himalayan | 0 | 1 | | 0 | 1 | |
| *Carex congdonii\|STARR\|B1079\|CAL\|spm00001686* | Non-Himalayan | 0 | 1 | | 0 | 1 | |
| *Carex congolensis\|GEHRKE\|NA\|MLW\|spm00000181* | Non-Himalayan | 0 | 1 | | 0 | 1 | |
| *Carex coninux\|SHUREN\|K_pusilla_ZSL\|\|spm00003655* | Both | 1 | 1 | | 1 | 1 | |
| *Carex conjuncta\|STARR\|B166\|DEL\|spm00000784* | Non-Himalayan | 0 | 1 | | 0 | 1 | |
| *Carex conoidea\|STARR\|B825\|DEL\|spm00001428* | Non-Himalayan | 0 | 1 | | 0 | 1 | |
| *Carex cordillerana\|STARR\|B943\|BRC\|spm00001544* | Non-Himalayan | 0 | 1 | | 0 | 1 | |
| *Carex corrugata\|STARR\|B051\|ALA\|spm00000670* | Non-Himalayan | 0 | 1 | | 0 | 1 | |
| *Carex corynoidea\|STARR_FORD\|STA_139\|NZ?\|spm00005087* | Non-Himalayan | 0 | 1 | | 0 | 1 | |
| *Carex coulteri\|REZNICEK\|NA\|MXC\|spm00000268* | Non-Himalayan | 0 | 1 | | 0 | 1 | |
| *Carex crawei\|STARR\|B868\|NFL\|spm00001471* | Non-Himalayan | 0 | 1 | | 0 | 1 | |
| *Carex crawfordii\|HIPP\|NA\|ILL\|spm00002161* | Non-Himalayan | 0 | 1 | | 0 | 1 | |
| *Carex crebra\|JIMENEZMEJIAS\|\|CHT\|spm00003392* | Non-Himalayan | 0 | 1 | | 1 | 1 | |
| *Carex crebriflora\|STARR\|B425\|\|spm00001043* | Non-Himalayan | 0 | 1 | | 0 | 1 | |
| *Carex cretica\|JIMENEZMEJIAS\|\|KRI\|spm00004382* | Non-Himalayan | 0 | 1 | | 0 | 1 | |
| *Carex crinalis\|JIMENEZMEJIAS\|NA\|ECU\|spm00000602* | Non-Himalayan | 0 | 1 | | 0 | 1 | |
| *Carex crinita\|STARR_FORD\|\|QUE\|spm00005233* | Non-Himalayan | 0 | 1 | | 0 | 1 | |
| *Carex crispa\|JIMENEZMEJIAS\|\|\|spm00004422* | Non-Himalayan | 0 | 1 | | 0 | 1 | |
| *Carex cristatella\|STARR\|B182\|KTY\|spm00000800* | Non-Himalayan | 0 | 1 | | 0 | 1 | |
| *Carex cruenta\|JIMENEZMEJIAS\|\|EHM\|spm00003357* | Both | 1 | 1 | | 1 | 0 | |
| *Carex cruscorvi\|STARR\|B204\|FLA\|spm00000822* | Non-Himalayan | 0 | 1 | | 0 | 1 | |
| *Carex cryptolepis\|STARR\|B1123\|ILL\|spm00001730* | Non-Himalayan | 0 | 1 | | 0 | 1 | |
| *Carex cumberlandensis\|STARR\|B199\|ARK\|spm00000817* | Non-Himalayan | 0 | 1 | | 0 | 1 | |
| *Carex cumulata\|STARR\|B253\|ILL\|spm00000871* | Non-Himalayan | 0 | 1 | | 0 | 1 | |
| *Carex curaica\|HIPP\|curaica_sn\|TVA\|spm00002340* | Both | 1 | 1 | | 1 | 1 | |
| *Carex curatorum\|STARR\|B1409\|\|spm00002016* | Non-Himalayan | 0 | 1 | | 0 | 1 | |
| *Carex curticeps\|STARR_FORD\|STA_2\|EHM\|spm00005191* | Both | 1 | 1 | | 1 | 0 | |
| *Carex curvula\|STARR_FORD\|STA_100\|FRA\|spm00005184* | Non-Himalayan | 0 | 1 | | 0 | 1 | |
| *Carex cusickii\|STARR\|B1397\|BRC\|spm00002004* | Non-Himalayan | 0 | 1 | | 0 | 1 | |
| *Carex cyanea\|STARR_FORD\|STA_56\|NZ?\|spm00005097* | Non-Himalayan | 0 | 1 | | 0 | 1 | |
| *Carex dacica\|LUCENO\|\|Iceland\|spm00003155* | Non-Himalayan | 0 | 1 | | 0 | 1 | |
| *Carex daltonii\|EHM\|E00047630* | Himalayan | 1 | 0 | | 1 | 0 | |
| *Carex dapanshanica\|XIAOFENG\|NA\|CHS\|spm00000039* | Non-Himalayan | 0 | 1 | | 0 | 1 | |
| *Carex darwinii\|LUCENO\|NA\|CLS\|spm00000571* | Non-Himalayan | 0 | 1 | | 0 | 1 | |
| *Carex dasycarpa\|STARR\|B255\|GEO\|spm00000873* | Non-Himalayan | 0 | 1 | | 0 | 1 | |
| *Carex davalliana\|WATERWAY\|davaliana_2416\|AUT\|spm00002350* | Non-Himalayan | 0 | 1 | | 0 | 1 | |
| *Carex davidii\|XIAOFENG\|NA\|CHN\|spm00000040* | Non-Himalayan | 0 | 1 | | 0 | 1 | |
| *Carex davisii\|STARR\|B240\|DEL\|spm00000858* | Non-Himalayan | 0 | 1 | | 0 | 1 | |
| *Carex davyi\|STARR\|B856\|CAL\|spm00001459* | Non-Himalayan | 0 | 1 | | 0 | 1 | |
| *Carex deasyi\|STARR_FORD\|STA_7\|EHM\|spm00005198* | Both | 1 | 1 | | 1 | 1 | |
| *Carex debilis\|STARR\|B245\|OKL\|spm00000863* | Non-Himalayan | 0 | 1 | | 0 | 1 | |
| *Carex decidua\|LUCENO\|NA\|CLS\|spm00000570* | Non-Himalayan | 0 | 1 | | 0 | 1 | |
| *Carex decomposita\|STARR\|B218\|DEL\|spm00000836* | Non-Himalayan | 0 | 1 | | 0 | 1 | |
| *Carex decurtata\|JIMENEZMEJIAS\|\|\|spm00004414* | Non-Himalayan | 0 | 1 | | 0 | 1 | |
| *Carex delicata\|JIMENEZMEJIAS\|\|TZK\|spm00003358* | Non-Himalayan | 0 | 1 | | 0 | 1 | |
| *Carex demissa\|STARR\|B1341\|NSC\|spm00001948* | Both | 1 | 1 | | 1 | 1 | |
| *Carex densa\|STARR\|B1062\|ORE\|spm00001669* | Non-Himalayan | 0 | 1 | | 0 | 1 | |
| *Carex densipilosa\|XIAOFENG\|NA\|CHS\|spm00000042* | Non-Himalayan | 0 | 1 | | 0 | 1 | |
| *Carex depauperata\|LUCENO\|NA\|YUG\|spm00000574* | Non-Himalayan | 0 | 1 | | 0 | 1 | |
| *Carex derelicta\|JIMENEZMEJIAS\|\|CZE\|spm00003350* | Non-Himalayan | 0 | 1 | | 0 | 1 | |
| *Carex deweyana\|HIPP\|deweyana_3162\|WIS\|spm00002359* | Non-Himalayan | 0 | 1 | | 0 | 1 | |
| *Carex diandra\|STARR\|B222\|CAL\|spm00000840* | Both | 1 | 1 | | 1 | 1 | |
| *Carex diastena\|MAGUILLA\|\|\|spm00006010* | Non-Himalayan | 0 | 1 | | 0 | 1 | |
| *Carex digitata\|LUCENO\|NA\|ITA\|spm00000569* | Non-Himalayan | 0 | 1 | | 0 | 1 | |
| *Carex diluta\|JIMENEZMEJIAS\|\|TCS\|spm00004364* | Both | 1 | 1 | | 1 | 1 | |
| *Carex dioica\|STARR_FORD\|STA_107\|GRB\|spm00005178* | Non-Himalayan | 0 | 1 | | 0 | 1 | |
| *Carex disperma\|STARR\|B1045\|ARK\|spm00001652* | Non-Himalayan | 0 | 1 | | 0 | 1 | |
| *Carex distachya\|LUCENO\|NA\|SPA\|spm00000588* | Non-Himalayan | 0 | 1 | | 0 | 1 | |
| *Carex distans\|STARR\|B226\|GRC\|spm00000844* | Non-Himalayan | 0 | 1 | | 0 | 1 | |
| *Carex disticha\|STARR_FORD\|STA_805\|ONT\|spm00005395* | Non-Himalayan | 0 | 1 | | 0 | 1 | |
| *Carex divisa\|STARR_FORD\|\|MAN\|spm00005374* | Both | 1 | 1 | | 1 | 1 | |
| *Carex divulsa\|JIMENEZMEJIAS\|\|FRA\|spm00004390* | Non-Himalayan | 0 | 1 | | 0 | 1 | |
| *Carex doniana\|XIAOFENG\|NA\|CHS\|spm00000047* | Both | 1 | 1 | | 1 | 1 | |
| *Carex douglasii\|STARR_FORD\|STA_807\|COL\|spm00005243* | Non-Himalayan | 0 | 1 | | 0 | 1 | |
| *Carex drucei\|JIMENEZMEJIAS\|\|\|spm00004418* | Non-Himalayan | 0 | 1 | | 0 | 1 | |
| *Carex durangensis\|REZNICEK\|NA\|MXE\|spm00000272* | Non-Himalayan | 0 | 1 | | 0 | 1 | |
| *Carex durieui\|JIMENEZMEJIAS\|\|SPA\|spm00004386* | Non-Himalayan | 0 | 1 | | 0 | 1 | |
| *Carex duriuscula\|STARR\|B1046\|ARK\|spm00001653* | Non-Himalayan | 0 | 1 | | 0 | 1 | |
| *Carex ebenea\|STARR\|B1263\|ARI\|spm00001870* | Non-Himalayan | 0 | 1 | | 0 | 1 | |
| *Carex eburnea\|STARR\|B224\|MIC\|spm00000842* | Non-Himalayan | 0 | 1 | | 0 | 1 | |
| *Carex echinata\|STARR_FORD\|STA_110\|GRB\|spm00005186* | Both | 1 | 1 | | 1 | 1 | |
| *Carex echinochloe\|GEHRKE\|Carex MOR_908\|\|spm00004701* | Non-Himalayan | 0 | 1 | | 0 | 1 | |
| *Carex echinodes\|HIPP\|ten.echinodes_3158\|WIS\|spm00002699* | Non-Himalayan | 0 | 1 | | 0 | 1 | |
| *Carex ecklonii\|JIMENEZMEJIAS\|\|CPP\|spm00004378* | Non-Himalayan | 0 | 1 | | 0 | 1 | |
| *Carex ecuadorensis\|STARR_FORD\|STA_148\|ECU\|spm00005088* | Non-Himalayan | 0 | 1 | | 0 | 1 | |
| *Carex edura\|K_FORD\|NA\|NZS\|spm00002184* | Non-Himalayan | 0 | 1 | | 0 | 1 | |
| *Carex edwardsiana\|STARR\|B1536\|TEX\|spm00002142* | Non-Himalayan | 0 | 1 | | 0 | 1 | |
| *Carex egena\|SANGTAE\|D0265\|KOR?\|spm00000329* | Non-Himalayan | 0 | 1 | | 0 | 1 | |
| *Carex egglestonii\|STARR\|B1095\|CAL\|spm00001702* | Non-Himalayan | 0 | 1 | | 0 | 1 | |
| *Carex egmontiana\|JIMENEZMEJIAS\|\|\|spm00004416* | Non-Himalayan | 0 | 1 | | 0 | 1 | |
| *Carex ehrenbergiana\|REZNICEK\|NA\|MXC\|spm00000298* | Non-Himalayan | 0 | 1 | | 0 | 1 | |
| *Carex eleusinoides\|STARR\|B1016\|ARK\|spm00001623* | Non-Himalayan | 0 | 1 | | 0 | 1 | |
| *Carex elgonensis\|GEHRKE\|NA\|UGA\|spm00000189* | Non-Himalayan | 0 | 1 | | 0 | 1 | |
| *Carex elliottii\|STARR\|B1309\|FLA\|spm00001916* | Non-Himalayan | 0 | 1 | | 0 | 1 | |
| *Carex elongata\|MAGUILLA\|\|\|spm00005946* | Non-Himalayan | 0 | 1 | | 0 | 1 | |
| *Carex emoryi\|STARR\|B267\|DEL\|spm00000885* | Non-Himalayan | 0 | 1 | | 0 | 1 | |
| *Carex endlichii\|STARR\|B1489\|ARI\|spm00002095* | Non-Himalayan | 0 | 1 | | 0 | 1 | |
| *Carex engelmannii\|STARR\|B1525\|BRC\|spm00002131* | Non-Himalayan | 0 | 1 | | 0 | 1 | |
| *Carex enneastachya\|JIMENEZMEJIAS\|NA\|ECU\|spm00000600* | Non-Himalayan | 0 | 1 | | 0 | 1 | |
| *Carex erebus\|JIMENEZMEJIAS\|\|\|spm00004424* | Non-Himalayan | 0 | 1 | | 0 | 1 | |
| *Carex ericetorum\|LUCENO\|NA\|ITA\|spm00000551* | Non-Himalayan | 0 | 1 | | 0 | 1 | |
| *Carex erinacea\|STARR_FORD\|STA_142\|CLC\|spm00005214* | Non-Himalayan | 0 | 1 | | 0 | 1 | |
| *Carex eriophylla\|JIMENEZMEJIAS\|NA\|CHM\|spm00003460* | Non-Himalayan | 0 | 1 | | 0 | 1 | |
| *Carex erythrorrhiza\|GEHRKE\|NA\|ETH\|spm00000191* | Non-Himalayan | 0 | 1 | | 0 | 1 | |
| *Carex erythrovaginata\|STARR_FORD\|STA_55\|NZ?\|spm00005096* | Non-Himalayan | 0 | 1 | | 0 | 1 | |
| *Carex esenbeckii\|STARR_FORD\|STA_5\|EHM\|spm00005193* | Both | 1 | 1 | | 1 | 1 | |
| *Carex euryphylla\|HIPP\|NA\|MDG\|spm00002218* | Non-Himalayan | 0 | 1 | | 0 | 1 | |
| *Carex exilis\|STARR\|B269\|DEL\|spm00000887* | Non-Himalayan | 0 | 1 | | 0 | 1 | |
| *Carex exsiccata\|STARR\|B1369\|BRC\|spm00001976* | Non-Himalayan | 0 | 1 | | 0 | 1 | |
| *Carex extensa\|STARR\|B1246\|VRG\|spm00001853* | Non-Himalayan | 0 | 1 | | 0 | 1 | |
| *Carex fecunda\|STARR_FORD\|STA_814\|BOL\|spm00005222* | Non-Himalayan | 0 | 1 | | 0 | 1 | |
| *Carex fedia\|JIMENEZMEJIAS\|NA\|PAK\|spm00003461* | Both | 1 | 1 | | 1 | 1 | |
| *Carex festucacea\|STARR\|B274\|TEX\|spm00000892* | Non-Himalayan | 0 | 1 | | 0 | 1 | |
| *Carex feta\|STARR\|B275\|CAL\|spm00000893* | Non-Himalayan | 0 | 1 | | 0 | 1 | |
| *Carex filicina\|PAK\|183073* | Both | 1 | 1 | | 1 | 1 | |
| *Carex filifolia\|STARR_FORD\|\|MAN\|spm00005150* | Non-Himalayan | 0 | 1 | | 0 | 1 | |
| *Carex filipes\|JIMENEZMEJIAS\|NA\|CHM\|spm00003462* | Non-Himalayan | 0 | 1 | | 0 | 1 | |
| *Carex filispica\|JIMENEZMEJIAS\|\|CHC\|spm00003398* | Both | 1 | 1 | | 1 | 0 | |
| *Carex fimbriata\|LUCENO\|NA\|SWI\|spm00000552* | Non-Himalayan | 0 | 1 | | 0 | 1 | |
| *Carex finitima\|CHC\|E00269484* | Both | 1 | 1 | | 1 | 1 | |
| *Carex firma\|LUCENO\|NA\|ITA\|spm00000520* | Non-Himalayan | 0 | 1 | | 0 | 1 | |
| *Carex firmula\|STARR_FORD\|STA_17\|ECU\|spm00005111* | Non-Himalayan | 0 | 1 | | 0 | 1 | |
| *Carex fischeri\|JIMENEZMEJIAS\|\|ETH\|spm00004352* | Non-Himalayan | 0 | 1 | | 0 | 1 | |
| *Carex fissa\|STARR_FORD\|STA_815\|FLA\|spm00005400* | Non-Himalayan | 0 | 1 | | 0 | 1 | |
| *Carex fissiglumis\|EHM\|E00693544* | Both | 1 | 1 | | 1 | 0 | |
| *Carex fissirostris\|JIMENEZMEJIAS\|\|MOR\|spm00004379* | Non-Himalayan | 0 | 1 | | 0 | 1 | |
| *Carex fissuricola\|STARR\|B1511\|IDA\|spm00002117* | Non-Himalayan | 0 | 1 | | 0 | 1 | |
| *Carex flacca\|STARR\|B1202\|CAL\|spm00001809* | Non-Himalayan | 0 | 1 | | 1 | 1 | |
| *Carex flaccosperma\|STARR\|B052\|LOU\|spm00000671* | Non-Himalayan | 0 | 1 | | 0 | 1 | |
| *Carex flava\|STARR\|B881\|NFL\|spm00001483* | Non-Himalayan | 0 | 1 | | 0 | 1 | |
| *Carex floridana\|STARR\|B288\|FLA\|spm00000906* | Non-Himalayan | 0 | 1 | | 0 | 1 | |
| *Carex fluviatilis\|HIPP\|fluviatilis_321\|CHC\|spm00002407* | Non-Himalayan | 0 | 1 | | 1 | 1 | |
| *Carex foenea\|STARR\|B1393\|BRC\|spm00002000* | Non-Himalayan | 0 | 1 | | 0 | 1 | |
| *Carex foetida\|LUCENO\|NA\|SWI\|spm00000565* | Non-Himalayan | 0 | 1 | | 0 | 1 | |
| *Carex folliculata\|STARR\|B293\|DEL\|spm00000911* | Non-Himalayan | 0 | 1 | | 0 | 1 | |
| *Carex foraminata\|XIAOFENG\|NA\|CHS\|spm00000052* | Non-Himalayan | 0 | 1 | | 0 | 1 | |
| *Carex formosa\|STARR\|B1125\|ILL\|spm00001732* | Non-Himalayan | 0 | 1 | | 0 | 1 | |
| *Carex forsteri\|K_FORD\|NA\|NZS\|spm00002183* | Non-Himalayan | 0 | 1 | | 0 | 1 | |
| *Carex fracta\|STARR\|B278\|CAL\|spm00000896* | Non-Himalayan | 0 | 1 | | 0 | 1 | |
| *Carex frankii\|STARR\|B321\|ALA\|spm00000939* | Non-Himalayan | 0 | 1 | | 0 | 1 | |
| *Carex fraseriana\|STARR\|B334\|MRY\|spm00000952* | Non-Himalayan | 0 | 1 | | 0 | 1 | |
| *Carex frigida\|LUCENO\|NA\|SPA\|spm00000521* | Non-Himalayan | 0 | 1 | | 0 | 1 | |
| *Carex fritschii\|MESTERHAZY\|NA\|HUN\|spm00002174* | Non-Himalayan | 0 | 1 | | 0 | 1 | |
| *Carex fucata\|CHC\|5778634* | Himalayan | 1 | 0 | | 1 | 0 | |
| *Carex fuliginosa\|STARR\|B1020\|ARK\|spm00001627* | Non-Himalayan | 0 | 1 | | 0 | 1 | |
| *Carex furva\|MAGUILLA\|\|\|spm00006011* | Non-Himalayan | 0 | 1 | | 0 | 1 | |
| *Carex fuscula\|JIMENEZMEJIAS\|\|CLC\|spm00004370* | Non-Himalayan | 0 | 1 | | 0 | 1 | |
| *Carex fusiformis\|EHM\|E00666305* | Himalayan | 1 | 0 | | 1 | 0 | |
| *Carex gammiei\|SHUREN\|K_gammiei_LJQ\|\|spm00003640* | Both | 1 | 1 | | 1 | 0 | |
| *Carex garberi\|STARR\|B1017\|ARK\|spm00001624* | Non-Himalayan | 0 | 1 | | 0 | 1 | |
| *Carex gayana\|LUCENO\|NA\|CLS\|spm00000573* | Non-Himalayan | 0 | 1 | | 0 | 1 | |
| *Carex genkaiensis\|HIPP\|NA\|KOR\|spm00000011* | Non-Himalayan | 0 | 1 | | 0 | 1 | |
| *Carex gentilis\|CHC\|5306057* | Non-Himalayan | 0 | 1 | | 1 | 1 | |
| *Carex geophila\|STARR\|B322\|TEX\|spm00000940* | Non-Himalayan | 0 | 1 | | 0 | 1 | |
| *Carex geyeri\|STARR\|B324\|ABT\|spm00000942* | Non-Himalayan | 0 | 1 | | 0 | 1 | |
| *Carex gholsonii\|STARR\|B335\|ALA\|spm00000953* | Non-Himalayan | 0 | 1 | | 0 | 1 | |
| *Carex gibba\|STARR_FORD\|STA_816\|CHH\|spm00005244* | Non-Himalayan | 0 | 1 | | 0 | 1 | |
| *Carex gifuensis\|SANGTAE\|E0180\|KOR?\|spm00000331* | Non-Himalayan | 0 | 1 | | 0 | 1 | |
| *Carex gigantea\|STARR\|B299\|FLA\|spm00000917* | Non-Himalayan | 0 | 1 | | 0 | 1 | |
| *Carex giraldiana\|XIAOFENG\|NA\|CHN\|spm00000054* | Non-Himalayan | 0 | 1 | | 0 | 1 | |
| *Carex glabrescens\|SANGTAE\|D0449\|KOR?\|spm00000332* | Non-Himalayan | 0 | 1 | | 0 | 1 | |
| *Carex glacialis\|STARR\|B887\|NFL\|spm00001488* | Non-Himalayan | 0 | 1 | | 0 | 1 | |
| *Carex glareosa\|MAGUILLA\|\|\|spm00004628* | Non-Himalayan | 0 | 1 | | 0 | 1 | |
| *Carex glaucescens\|STARR\|B308\|MSI\|spm00000926* | Non-Himalayan | 0 | 1 | | 0 | 1 | |
| *Carex glaucodea\|STARR\|B924\|INI\|spm00001525* | Non-Himalayan | 0 | 1 | | 0 | 1 | |
| *Carex globosa\|STARR\|B302\|CAL\|spm00000920* | Non-Himalayan | 0 | 1 | | 0 | 1 | |
| *Carex globularis\|JIMENEZMEJIAS\|\|YAK\|spm00004387* | Non-Himalayan | 0 | 1 | | 0 | 1 | |
| *Carex glomerabilis\|GEHRKE\|NA\|CPP\|spm00000193* | Non-Himalayan | 0 | 1 | | 0 | 1 | |
| *Carex gmelinii\|STARR\|B1022\|ARK\|spm00001629* | Non-Himalayan | 0 | 1 | | 0 | 1 | |
| *Carex godfreyi\|STARR\|B1180\|ALA\|spm00001787* | Non-Himalayan | 0 | 1 | | 0 | 1 | |
| *Carex gongshanensis\|JIMENEZMEJIAS\|\|CHT\|spm00003361* | Non-Himalayan | 0 | 1 | | 1 | 0 | |
| *Carex gotoi\|HIPP\|NA\|KOR\|spm00000020* | Non-Himalayan | 0 | 1 | | 0 | 1 | |
| *Carex gracilior\|STARR\|B1085\|CAL\|spm00001692* | Non-Himalayan | 0 | 1 | | 0 | 1 | |
| *Carex gracillima\|HIPP\|gracillima_505\|WIS\|spm00002423* | Non-Himalayan | 0 | 1 | | 0 | 1 | |
| *Carex graminifolia\|GEHRKE\|NA\|MDG\|spm00000195* | Non-Himalayan | 0 | 1 | | 0 | 1 | |
| *Carex grandiligulata\|XIAOFENG\|NA\|CHS\|spm00000056* | Non-Himalayan | 0 | 1 | | 0 | 1 | |
| *Carex granularis\|STARR\|B804\|MAN\|spm00001407* | Non-Himalayan | 0 | 1 | | 0 | 1 | |
| *Carex gravida\|STARR\|B352\|TEN\|spm00000970* | Non-Himalayan | 0 | 1 | | 0 | 1 | |
| *Carex grayi\|STARR\|B306\|TEN\|spm00000924* | Non-Himalayan | 0 | 1 | | 0 | 1 | |
| *Carex greenwayi\|JIMENEZMEJIAS\|\|TAN\|spm00004344* | Non-Himalayan | 0 | 1 | | 0 | 1 | |
| *Carex grioletii\|LUCENO\|NA\|SPA\|spm00000585* | Non-Himalayan | 0 | 1 | | 0 | 1 | |
| *Carex grisea\|STARR\|B037\|KTY\|spm00000656* | Non-Himalayan | 0 | 1 | | 0 | 1 | |
| *Carex gunniana\|JIMENEZMEJIAS\|\|TAS\|spm00004360* | Non-Himalayan | 0 | 1 | | 0 | 1 | |
| *Carex gynandra\|STARR\|B346\|DEL\|spm00000964* | Non-Himalayan | 0 | 1 | | 0 | 1 | |
| *Carex gynocrates\|STARR\|B1023\|ARK\|spm00001630* | Non-Himalayan | 0 | 1 | | 0 | 1 | |
| *Carex gynodynama\|STARR\|B1081\|CAL\|spm00001688* | Non-Himalayan | 0 | 1 | | 0 | 1 | |
| *Carex gypsophila\|REZNICEK\|NA\|MXE\|spm00000361* | Non-Himalayan | 0 | 1 | | 0 | 1 | |
| *Carex haematostoma\|JIMENEZMEJIAS\|\|CHT\|spm00003383* | Both | 1 | 1 | | 1 | 1 | |
| *Carex halleriana\|LUCENO\|NA\|SPA\|spm00000557* | Non-Himalayan | 0 | 1 | | 1 | 1 | |
| *Carex halliana\|STARR\|B1082\|CAL\|spm00001689* | Non-Himalayan | 0 | 1 | | 0 | 1 | |
| *Carex hallii\|STARR\|B1147\|MAN\|spm00001754* | Non-Himalayan | 0 | 1 | | 0 | 1 | |
| *Carex hamata\|STARR_FORD\|STA_145\|ECU\|spm00005093* | Non-Himalayan | 0 | 1 | | 0 | 1 | |
| *Carex hancockiana\|JIMENEZMEJIAS\|NA\|CHM\|spm00003463* | Non-Himalayan | 0 | 1 | | 0 | 1 | |
| *Carex handelmazzettii\|SHUREN\|K_handcl_mazzettii_LJQ\|\|spm00003629* | Both | 1 | 1 | | 1 | 0 | |
| *Carex harae\|EHM\|E00048402* | Himalayan | 1 | 0 | | 1 | 0 | |
| *Carex harfordii\|STARR\|B347\|CAL\|spm00000965* | Non-Himalayan | 0 | 1 | | 0 | 1 | |
| *Carex hartmanii\|GEBAUER\|\|GER\|spm00006155* | Non-Himalayan | 0 | 1 | | 0 | 1 | |
| *Carex hassei\|STARR\|B1260\|CAL\|spm00001867* | Non-Himalayan | 0 | 1 | | 0 | 1 | |
| *Carex hastata\|XIAOFENG\|NA\|CHS\|spm00000058* | Non-Himalayan | 0 | 1 | | 0 | 1 | |
| *Carex haydeniana\|HIPP\|haydeniana_140.2\|UTA\|spm00002432* | Non-Himalayan | 0 | 1 | | 0 | 1 | |
| *Carex haydenii\|HIPP\|haydenii_501\|WIS\|spm00002433* | Non-Himalayan | 0 | 1 | | 0 | 1 | |
| *Carex healyi\|STARR_FORD\|STA_137\|NZN\|spm00005107* | Non-Himalayan | 0 | 1 | | 0 | 1 | |
| *Carex hebes\|WATERWAY\|hebes_2329\|NSW\|spm00002434* | Non-Himalayan | 0 | 1 | | 0 | 1 | |
| *Carex heleonastes\|STARR\|B1347\|ABT\|spm00001954* | Non-Himalayan | 0 | 1 | | 0 | 1 | |
| *Carex helleri\|STARR\|B861\|CAL\|spm00001464* | Non-Himalayan | 0 | 1 | | 0 | 1 | |
| *Carex helodes\|JIMENEZMEJIAS\|\|MOR\|spm00004359* | Non-Himalayan | 0 | 1 | | 0 | 1 | |
| *Carex hendersonii\|STARR\|B1340\|BRC\|spm00001947* | Non-Himalayan | 0 | 1 | | 0 | 1 | |
| *Carex heterodoxa\|HIPP\|NA\|MDG\|spm00002213* | Non-Himalayan | 0 | 1 | | 0 | 1 | |
| *Carex heteroneura\|STARR\|B307\|NEV\|spm00000925* | Non-Himalayan | 0 | 1 | | 0 | 1 | |
| *Carex heterostachya\|JIMENEZMEJIAS\|NA\|CHN\|spm00003464* | Non-Himalayan | 0 | 1 | | 0 | 1 | |
| *Carex hirsutella\|STARR\|B317\|DEL\|spm00000935* | Non-Himalayan | 0 | 1 | | 0 | 1 | |
| *Carex hirta\|STARR\|B329\|PEN\|spm00000947* | Non-Himalayan | 0 | 1 | | 0 | 1 | |
| *Carex hirtifolia\|STARR\|B872\|QUE\|spm00001475* | Non-Himalayan | 0 | 1 | | 0 | 1 | |
| *Carex hirtigluma\|GEHRKE\|NA\|MDG\|spm00000196* | Non-Himalayan | 0 | 1 | | 0 | 1 | |
| *Carex hirtissima\|STARR\|B1087\|CAL\|spm00001694* | Non-Himalayan | 0 | 1 | | 0 | 1 | |
| *Carex hispida\|LUCENO\|NA\|KRI\|spm00000549* | Non-Himalayan | 0 | 1 | | 0 | 1 | |
| *Carex hitchcockiana\|STARR\|B038\|KTY\|spm00000657* | Non-Himalayan | 0 | 1 | | 0 | 1 | |
| *Carex hochstetteriana\|ESCUDERO\|\|POR\|spm00002856* | Non-Himalayan | 0 | 1 | | 0 | 1 | |
| *Carex holostoma\|STARR\|B1024\|ARK\|spm00001631* | Non-Himalayan | 0 | 1 | | 0 | 1 | |
| *Carex hoodii\|STARR\|B1323\|CAL\|spm00001930* | Non-Himalayan | 0 | 1 | | 0 | 1 | |
| *Carex hookeriana\|STARR_FORD\|STA_819\|MAN\|spm00005409* | Non-Himalayan | 0 | 1 | | 0 | 1 | |
| *Carex horizontalis\|STARR_FORD\|STA_73\|NZS\|spm00005106* | Non-Himalayan | 0 | 1 | | 0 | 1 | |
| *Carex hormathodes\|STARR\|B1508\|NSC\|spm00002114* | Non-Himalayan | 0 | 1 | | 0 | 1 | |
| *Carex hostiana\|LUCENO\|\|Germany\|spm00003273* | Non-Himalayan | 0 | 1 | | 0 | 1 | |
| *Carex houghtoniana\|STARR\|B843\|MAN\|spm00001446* | Non-Himalayan | 0 | 1 | | 0 | 1 | |
| *Carex hughii\|SHUREN\|K_graminifolia_F_ZSL\|\|spm00003650* | Both | 1 | 1 | | 1 | 1 | |
| *Carex hultenii\|REZNICEK\|NA\|MXE\|spm00000273* | Non-Himalayan | 0 | 1 | | 0 | 1 | |
| *Carex humbertiana\|HIPP\|NA\|KOR\|spm00000006* | Non-Himalayan | 0 | 1 | | 0 | 1 | |
| *Carex humboldtiana\|REZNICEK\|NA\|MXC\|spm00000271* | Non-Himalayan | 0 | 1 | | 0 | 1 | |
| *Carex hyalina\|STARR\|B294\|MSI\|spm00000912* | Non-Himalayan | 0 | 1 | | 0 | 1 | |
| *Carex hyalinolepis\|STARR\|B338\|TEX\|spm00000956* | Non-Himalayan | 0 | 1 | | 0 | 1 | |
| *Carex hypochlora\|XIAOFENG\|NA\|CHN\|spm00000060* | Non-Himalayan | 0 | 1 | | 0 | 1 | |
| *Carex hystericina\|STARR\|B475\|ARK\|spm00001081* | Non-Himalayan | 0 | 1 | | 0 | 1 | |
| *Carex idaea\|JIMENEZMEJIAS\|\|KRI\|spm00004380* | Non-Himalayan | 0 | 1 | | 0 | 1 | |
| *Carex idahoa\|STARR\|B956\|MNT\|spm00001563* | Non-Himalayan | 0 | 1 | | 0 | 1 | |
| *Carex iljinii\|HIPP\|iljinii_1964\|BRY\|spm00002443* | Non-Himalayan | 0 | 1 | | 0 | 1 | |
| *Carex illota\|STARR\|B343\|CAL\|spm00000961* | Non-Himalayan | 0 | 1 | | 0 | 1 | |
| *Carex imbecilla\|JIMENEZMEJIAS\|\|\|spm00004419* | Non-Himalayan | 0 | 1 | | 0 | 1 | |
| *Carex impressinervia\|STARR\|B1177\|MSI\|spm00001784* | Non-Himalayan | 0 | 1 | | 0 | 1 | |
| *Carex inanis\|JIMENEZMEJIAS\|\|CHT\|spm00003363* | Both | 1 | 1 | | 1 | 0 | |
| *Carex incomitata\|WATERWAY\|incomitata_1789\|NSW\|spm00002446* | Non-Himalayan | 0 | 1 | | 0 | 1 | |
| *Carex incurviformis\|STARR\|B1495\|COL\|spm00002101* | Non-Himalayan | 0 | 1 | | 0 | 1 | |
| *Carex indica\|HIPP\|NA\|QLD\|spm00002191* | Both | 1 | 1 | | 1 | 1 | |
| *Carex infirminervia\|STARR\|B795\|CAL\|spm00001398* | Non-Himalayan | 0 | 1 | | 0 | 1 | |
| *Carex infuscata\|PAK\|183079* | Both | 1 | 1 | | 1 | 0 | |
| *Carex integra\|STARR\|B359\|CAL\|spm00000977* | Non-Himalayan | 0 | 1 | | 0 | 1 | |
| *Carex interior\|STARR\|B354\|MIC\|spm00000972* | Non-Himalayan | 0 | 1 | | 0 | 1 | |
| *Carex interjecta\|HIPP\|interjecta_15398\|MXC\|spm00002454* | Non-Himalayan | 0 | 1 | | 0 | 1 | |
| *Carex interrupta\|STARR\|B1059\|ORE\|spm00001666* | Non-Himalayan | 0 | 1 | | 0 | 1 | |
| *Carex intumescens\|STARR\|B357\|MAI\|spm00000975* | Non-Himalayan | 0 | 1 | | 0 | 1 | |
| *Carex ischnostachya\|XIAOFENG\|NA\|CHS\|spm00000062* | Non-Himalayan | 0 | 1 | | 0 | 1 | |
| *Carex ivanoviae\|JIMENEZMEJIAS\|\|CHQ\|spm00003371* | Non-Himalayan | 0 | 1 | | 1 | 1 | |
| *Carex jackiana\|EHM\|E00666333* | Both | 1 | 1 | | 1 | 1 | |
| *Carex jamesii\|STARR\|B010\|ALA\|spm00000630* | Non-Himalayan | 0 | 1 | | 0 | 1 | |
| *Carex jamesonii\|JIMENEZMEJIAS\|NA\|VEN\|spm00000517* | Non-Himalayan | 0 | 1 | | 0 | 1 | |
| *Carex jiuhuaensis\|XIAOFENG\|NA\|CHS\|spm00000063* | Non-Himalayan | 0 | 1 | | 0 | 1 | |
| *Carex johnstonii\|GEHRKE\|NA\|UGA\|spm00000200* | Non-Himalayan | 0 | 1 | | 0 | 1 | |
| *Carex jonesii\|STARR\|B1003\|WAS\|spm00001610* | Non-Himalayan | 0 | 1 | | 0 | 1 | |
| *Carex joorii\|STARR\|B367\|FLA\|spm00000985* | Non-Himalayan | 0 | 1 | | 0 | 1 | |
| *Carex juniperorum\|STARR\|B011\|KTY\|spm00000631* | Non-Himalayan | 0 | 1 | | 0 | 1 | |
| *Carex kamagariensis\|SANGTAE\|D0218\|KOR?\|spm00000336* | Non-Himalayan | 0 | 1 | | 0 | 1 | |
| *Carex kanaii\|SHUREN\|K_kanai_LJQ\|\|spm00003637* | Himalayan | 1 | 0 | | 1 | 0 | |
| *Carex kansuensis\|JIMENEZMEJIAS\|\|CHC\|spm00003365* | Non-Himalayan | 0 | 1 | | 1 | 1 | |
| *Carex killickii\|STARR_FORD\|STA_47\|CPP\|spm00005203* | Non-Himalayan | 0 | 1 | | 0 | 1 | |
| *Carex kingii\|STARR_FORD\|STA_80\|CLS\|spm00005221* | Non-Himalayan | 0 | 1 | | 0 | 1 | |
| *Carex kirinensis\|JIMENEZMEJIAS\|\|CHT\|spm00003366* | Non-Himalayan | 0 | 1 | | 0 | 1 | |
| *Carex kirkii\|JIMENEZMEJIAS\|\|\|spm00004417* | Non-Himalayan | 0 | 1 | | 0 | 1 | |
| *Carex kitaibeliana\|LUCENO\|NA\|YUG\|spm00000539* | Non-Himalayan | 0 | 1 | | 0 | 1 | |
| *Carex klamathensis\|STARR\|B1463\|ORE\|spm00002069* | Non-Himalayan | 0 | 1 | | 0 | 1 | |
| *Carex kobomugi\|STARR\|B361\|VRG\|spm00000979* | Non-Himalayan | 0 | 1 | | 0 | 1 | |
| *Carex kobresiformis\|XIAOFENG\|NA\|CHS\|spm00000067* | Non-Himalayan | 0 | 1 | | 0 | 1 | |
| *Carex kokanica\|SHUREN\|K_minshanica_ZSR_K\|\|spm00003613* | Both | 1 | 1 | | 1 | 1 | |
| *Carex koshewnikowii\|JIMENEZMEJIAS\|NA\|TZK\|spm00003465* | Both | 1 | 1 | | 1 | 1 | |
| *Carex kraliana\|STARR\|B363\|ARK\|spm00000981* | Non-Himalayan | 0 | 1 | | 0 | 1 | |
| *Carex krausei\|STARR\|B1032\|ARK\|spm00001639* | Non-Himalayan | 0 | 1 | | 0 | 1 | |
| *Carex kumaonensis\|EHM\|E00656734* | Both | 1 | 1 | | 1 | 0 | |
| *Carex kurdica\|LUCENO\|\|Iran\|spm00003158* | Non-Himalayan | 0 | 1 | | 0 | 1 | |
| *Carex lachenalii\|STARR\|B1033\|ARK\|spm00001640* | Non-Himalayan | 0 | 1 | | 0 | 1 | |
| *Carex lacustris\|STARR\|B373\|MAN\|spm00000991* | Non-Himalayan | 0 | 1 | | 0 | 1 | |
| *Carex laeta\|JIMENEZMEJIAS\|\|CHT\|spm00003394* | Both | 1 | 1 | | 1 | 0 | |
| *Carex laeviconica\|STARR\|B1280\|TEN\|spm00001887* | Non-Himalayan | 0 | 1 | | 0 | 1 | |
| *Carex laeviculmis\|STARR\|B794\|CAL\|spm00001397* | Non-Himalayan | 0 | 1 | | 0 | 1 | |
| *Carex laevigata\|JIMENEZMEJIAS\|\|FRA\|spm00004373* | Non-Himalayan | 0 | 1 | | 0 | 1 | |
| *Carex laevissima\|STARR_FORD\|STA_828\|PRM\|spm00005425* | Non-Himalayan | 0 | 1 | | 0 | 1 | |
| *Carex laevivaginata\|STARR\|B375\|ALA\|spm00000993* | Non-Himalayan | 0 | 1 | | 0 | 1 | |
| *Carex lagunensis\|HIPP\|lagunensis_4482\|MXE\|spm00002467* | Non-Himalayan | 0 | 1 | | 0 | 1 | |
| *Carex lainzii\|JIMENEZMEJIAS\|\|SPA\|spm00004371* | Non-Himalayan | 0 | 1 | | 0 | 1 | |
| *Carex lancea\|STARR_FORD\|STA_48\|CPP\|spm00005204* | Non-Himalayan | 0 | 1 | | 0 | 1 | |
| *Carex lancifolia\|XIAOFENG\|NA\|CHN\|spm00000070* | Non-Himalayan | 0 | 1 | | 0 | 1 | |
| *Carex lapponica\|STARR\|B1034\|ARK\|spm00001641* | Non-Himalayan | 0 | 1 | | 0 | 1 | |
| *Carex larensis\|JIMENEZMEJIAS\|NA\|VEN\|spm00000601* | Non-Himalayan | 0 | 1 | | 0 | 1 | |
| *Carex lasiocarpa\|STARR\|B379\|ARK\|spm00000997* | Non-Himalayan | 0 | 1 | | 0 | 1 | |
| *Carex latebracteata\|STARR\|B779\|ARK\|spm00001383* | Non-Himalayan | 0 | 1 | | 0 | 1 | |
| *Carex lativena\|STARR\|B1222\|NWM\|spm00001829* | Non-Himalayan | 0 | 1 | | 0 | 1 | |
| *Carex laxa\|STARR\|B1036\|ARK\|spm00001643* | Non-Himalayan | 0 | 1 | | 0 | 1 | |
| *Carex laxiflora\|STARR\|B405\|MIC\|spm00001023* | Non-Himalayan | 0 | 1 | | 0 | 1 | |
| *Carex leavenworthii\|STARR_FORD\|STA_830\|KTY\|spm00005410* | Non-Himalayan | 0 | 1 | | 0 | 1 | |
| *Carex ledebouriana\|JIMENEZMEJIAS\|\|MAG\|spm00003592* | Non-Himalayan | 0 | 1 | | 0 | 1 | |
| *Carex leersii\|JIMENEZMEJIAS\|\|SPA\|spm00004393* | Both | 1 | 1 | | 1 | 1 | |
| *Carex lehmannii\|JIMENEZMEJIAS\|\|CHC\|spm00003367* | Both | 1 | 1 | | 1 | 1 | |
| *Carex leiorhyncha\|HIPP\|leiorhyncha_2001\|KOR\|spm00002471* | Non-Himalayan | 0 | 1 | | 0 | 1 | |
| *Carex lemmonii\|STARR\|B411\|CAL\|spm00001029* | Non-Himalayan | 0 | 1 | | 0 | 1 | |
| *Carex leporina\|STARR\|B1173\|WIS\|spm00001780* | Both | 1 | 1 | | 1 | 1 | |
| *Carex leporinella\|STARR\|B1006\|WAS\|spm00001613* | Non-Himalayan | 0 | 1 | | 0 | 1 | |
| *Carex leptalea\|STARR\|B414\|MIC\|spm00001032* | Non-Himalayan | 0 | 1 | | 0 | 1 | |
| *Carex leptocladus\|MESTERHAZY\|NA\|GGI\|spm00002180* | Non-Himalayan | 0 | 1 | | 0 | 1 | |
| *Carex leptonervia\|STARR\|B790\|MAN\|spm00001394* | Non-Himalayan | 0 | 1 | | 0 | 1 | |
| *Carex leptopoda\|STARR\|B796\|BRC\|spm00001399* | Non-Himalayan | 0 | 1 | | 0 | 1 | |
| *Carex leptosaccus\|GEHRKE\|NA\|UGA\|spm00000201* | Non-Himalayan | 0 | 1 | | 0 | 1 | |
| *Carex leucodonta\|REZNICEK\|NA\|MXE\|spm00000269* | Non-Himalayan | 0 | 1 | | 0 | 1 | |
| *Carex lianchengensis\|XIAOFENG\|NA\|CHS\|spm00000073* | Non-Himalayan | 0 | 1 | | 0 | 1 | |
| *Carex limosa\|STARR\|B417\|ARK\|spm00001035* | Non-Himalayan | 0 | 1 | | 0 | 1 | |
| *Carex lingii\|SHUREN\|C_lingii_BHY\|\|spm00003612* | Non-Himalayan | 0 | 1 | | 0 | 1 | |
| *Carex liparocarpos\|LUCENO\|NA\|SPA\|spm00000556* | Non-Himalayan | 0 | 1 | | 0 | 1 | |
| *Carex littledalei\|JIMENEZMEJIAS\|\|CHT\|spm00003404* | Non-Himalayan | 0 | 1 | | 1 | 0 | |
| *Carex litvinovii\|JIMENEZMEJIAS\|NA\|TZK\|spm00003467* | Non-Himalayan | 0 | 1 | | 0 | 1 | |
| *Carex livida\|STARR\|B807\|NWJ\|spm00001410* | Non-Himalayan | 0 | 1 | | 0 | 1 | |
| *Carex loliacea\|STARR\|B1043\|ARK\|spm00001650* | Non-Himalayan | 0 | 1 | | 0 | 1 | |
| *Carex lonchocarpa\|STARR\|B381\|FLA\|spm00000999* | Non-Himalayan | 0 | 1 | | 0 | 1 | |
| *Carex longicaulis\|REZNICEK\|NA\|MXG\|spm00000274* | Non-Himalayan | 0 | 1 | | 0 | 1 | |
| *Carex longicruris\|EHM\|E00693600* | Both | 1 | 1 | | 1 | 1 | |
| *Carex longifructus\|JIMENEZMEJIAS\|\|\|spm00004426* | Non-Himalayan | 0 | 1 | | 0 | 1 | |
| *Carex longii\|STARR\|B383\|ARK\|spm00001001* | Non-Himalayan | 0 | 1 | | 0 | 1 | |
| *Carex longipedunculata\|GEHRKE\|NA\|KEN\|spm00000167* | Non-Himalayan | 0 | 1 | | 0 | 1 | |
| *Carex longipes\|EHM\|E00693601* | Both | 1 | 1 | | 1 | 1 | |
| *Carex longirostrata\|XIAOFENG\|NA\|CHS\|spm00000077* | Non-Himalayan | 0 | 1 | | 0 | 1 | |
| *Carex longissima\|REZNICEK\|NA\|MXE\|spm00000275* | Non-Himalayan | 0 | 1 | | 0 | 1 | |
| *Carex louisianica\|STARR\|B390\|FLA\|spm00001008* | Non-Himalayan | 0 | 1 | | 0 | 1 | |
| *Carex lowei\|ESCUDERO\|\|MDR\|spm00002858* | Non-Himalayan | 0 | 1 | | 0 | 1 | |
| *Carex ludwigii\|GEHRKE\|NA\|NAT\|spm00000147* | Non-Himalayan | 0 | 1 | | 0 | 1 | |
| *Carex lupuliformis\|STARR\|B393\|FLA\|spm00001011* | Non-Himalayan | 0 | 1 | | 0 | 1 | |
| *Carex lupulina\|STARR\|B472\|MRY\|spm00001078* | Non-Himalayan | 0 | 1 | | 0 | 1 | |
| *Carex lurida\|STARR\|B476\|FLA\|spm00001082* | Non-Himalayan | 0 | 1 | | 0 | 1 | |
| *Carex lutea\|STARR\|B1158\|NCA\|spm00001765* | Non-Himalayan | 0 | 1 | | 0 | 1 | |
| *Carex luzulifolia\|STARR\|B410\|CAL\|spm00001028* | Non-Himalayan | 0 | 1 | | 0 | 1 | |
| *Carex lycurus\|GEHRKE\|NA\|MLW\|spm00000206* | Non-Himalayan | 0 | 1 | | 0 | 1 | |
| *Carex lyngbyei\|STARR\|B479\|ORE\|spm00001085* | Non-Himalayan | 0 | 1 | | 0 | 1 | |
| *Carex maackii\|HIPP\|maackii_8031\|JAP\|spm00002491* | Non-Himalayan | 0 | 1 | | 0 | 1 | |
| *Carex mabilliana\|JIMENEZMEJIAS\|NA\|COR\|spm00000594* | Non-Himalayan | 0 | 1 | | 0 | 1 | |
| *Carex mackenziei\|STARR\|B1050\|ARK\|spm00001657* | Non-Himalayan | 0 | 1 | | 0 | 1 | |
| *Carex macloviana\|HIPP\|macloviana_1008\|COL\|spm00002492* | Non-Himalayan | 0 | 1 | | 0 | 1 | |
| *Carex macrocephala\|STARR\|B831\|ARK\|spm00001434* | Non-Himalayan | 0 | 1 | | 0 | 1 | |
| *Carex macrochaeta\|STARR\|B1064\|ORE\|spm00001671* | Non-Himalayan | 0 | 1 | | 0 | 1 | |
| *Carex macrolepis\|LUCENO\|NA\|ITA\|spm00000536* | Non-Himalayan | 0 | 1 | | 0 | 1 | |
| *Carex macrophyllidion\|GEHRKE\|Carex MOR_913\|\|spm00004706* | Non-Himalayan | 0 | 1 | | 0 | 1 | |
| *Carex macroprophylla\|JIMENEZMEJIAS\|\|CHC\|spm00003400* | Non-Himalayan | 0 | 1 | | 0 | 1 | |
| *Carex macrosolen\|LUCENO\|NA\|AGS\|spm00000553* | Non-Himalayan | 0 | 1 | | 0 | 1 | |
| *Carex macrostachys\|LUCENO\|NA\|ITA\|spm00000523* | Non-Himalayan | 0 | 1 | | 0 | 1 | |
| *Carex macrostyla\|LUCENO\|\|Spain\|spm00003284* | Non-Himalayan | 0 | 1 | | 0 | 1 | |
| *Carex madagascariensis\|HIPP\|NA\|MDG\|spm00002210* | Non-Himalayan | 0 | 1 | | 0 | 1 | |
| *Carex madida\|STARR_FORD\|STA_14\|ECU\|spm00005095* | Non-Himalayan | 0 | 1 | | 0 | 1 | |
| *Carex madrensis\|REZNICEK\|NA\|MXS\|spm00000277* | Non-Himalayan | 0 | 1 | | 0 | 1 | |
| *Carex magacis\|JIMENEZMEJIAS\|\|SPA\|spm00004395* | Non-Himalayan | 0 | 1 | | 0 | 1 | |
| *Carex magellanica\|LUCENO\|\|Finland\|spm00003017* | Non-Himalayan | 0 | 1 | | 0 | 1 | |
| *Carex mairei\|JIMENEZMEJIAS\|\|SPA\|spm00004367* | Non-Himalayan | 0 | 1 | | 0 | 1 | |
| *Carex manhartii\|STARR\|B1154\|GEO\|spm00001761* | Non-Himalayan | 0 | 1 | | 0 | 1 | |
| *Carex mannii\|GEHRKE\|NA\|KEN\|spm00000210* | Non-Himalayan | 0 | 1 | | 0 | 1 | |
| *Carex marianensis\|REZNICEK\|NA\|MXE\|spm00000280* | Non-Himalayan | 0 | 1 | | 0 | 1 | |
| *Carex marina\|STARR\|B1139\|MAN\|spm00001746* | Non-Himalayan | 0 | 1 | | 0 | 1 | |
| *Carex mariposana\|STARR\|B1277\|ARI\|spm00001884* | Non-Himalayan | 0 | 1 | | 0 | 1 | |
| *Carex maritima\|WATERWAY\|maritima_1048\|YUK\|spm00002500* | Both | 1 | 1 | | 1 | 1 | |
| *Carex meadii\|STARR\|B1135\|MAN\|spm00001742* | Non-Himalayan | 0 | 1 | | 0 | 1 | |
| *Carex media\|STARR\|B1165\|NBR\|spm00001772* | Non-Himalayan | 0 | 1 | | 0 | 1 | |
| *Carex megalepis\|STARR_FORD\|STA_69\|NZ?\|spm00005089* | Non-Himalayan | 0 | 1 | | 0 | 1 | |
| *Carex melanantha\|JIMENEZMEJIAS\|\|KAZ\|spm00003368* | Both | 1 | 1 | | 1 | 1 | |
| *Carex melanocarpa\|JIMENEZMEJIAS\|\|KHA\|spm00003594* | Non-Himalayan | 0 | 1 | | 0 | 1 | |
| *Carex melanocephala\|GEBAUER\|\|ALT\|spm00006156* | Non-Himalayan | 0 | 1 | | 0 | 1 | |
| *Carex melanostachya\|STARR\|B947b\|\|spm00001550* | Non-Himalayan | 0 | 1 | | 0 | 1 | |
| *Carex membranacea\|STARR\|B442\|ASK\|spm00001050* | Non-Himalayan | 0 | 1 | | 0 | 1 | |
| *Carex mendocinensis\|STARR\|B1066\|ORE\|spm00001673* | Non-Himalayan | 0 | 1 | | 0 | 1 | |
| *Carex meridensis\|STARR_FORD\|STA_151\|ECU\|spm00005100* | Non-Himalayan | 0 | 1 | | 0 | 1 | |
| *Carex meridiana\|SANGTAE\|D0660\|KOR?\|spm00000338* | Non-Himalayan | 0 | 1 | | 0 | 1 | |
| *Carex merrittfernaldii\|STARR\|B443\|MAN\|spm00001051* | Non-Himalayan | 0 | 1 | | 0 | 1 | |
| *Carex mertensii\|STARR\|B448\|ASK\|spm00001056* | Non-Himalayan | 0 | 1 | | 0 | 1 | |
| *Carex mesochorea\|STARR\|B491\|ALA\|spm00001097* | Non-Himalayan | 0 | 1 | | 0 | 1 | |
| *Carex mesophila\|REZNICEK\|NA\|MXE\|spm00000364* | Non-Himalayan | 0 | 1 | | 0 | 1 | |
| *Carex meyeriana\|GEBAUER\|\|MON\|spm00006158* | Non-Himalayan | 0 | 1 | | 0 | 1 | |
| *Carex michauxiana\|STARR\|B1141\|MAN\|spm00001748* | Non-Himalayan | 0 | 1 | | 0 | 1 | |
| *Carex michelii\|MESTERHAZY\|NA\|HUN\|spm00002175* | Non-Himalayan | 0 | 1 | | 0 | 1 | |
| *Carex micrantha\|SANGTAE\|E0333\|KOR?\|spm00000339* | Non-Himalayan | 0 | 1 | | 0 | 1 | |
| *Carex microcarpa\|LUCENO\|18E1MMR11\|COR\|spm00003759* | Non-Himalayan | 0 | 1 | | 0 | 1 | |
| *Carex microdonta\|STARR\|B817\|MSI\|spm00001420* | Non-Himalayan | 0 | 1 | | 0 | 1 | |
| *Carex microglochin\|LUCENO\|NA\|GNL\|spm00000590* | Both | 1 | 1 | | 1 | 1 | |
| *Carex micropoda\|STARR\|B1025\|ARK\|spm00001632* | Non-Himalayan | 0 | 1 | | 0 | 1 | |
| *Carex microptera\|STARR\|B316\|NEV\|spm00000934* | Non-Himalayan | 0 | 1 | | 0 | 1 | |
| *Carex mildbraediana\|JIMENEZMEJIAS\|\|BUR\|spm00004361* | Non-Himalayan | 0 | 1 | | 0 | 1 | |
| *Carex minor\|JIMENEZMEJIAS\|\|\|spm00004412* | Non-Himalayan | 0 | 1 | | 0 | 1 | |
| *Carex misera\|STARR\|B1157\|TEN\|spm00001764* | Non-Himalayan | 0 | 1 | | 0 | 1 | |
| *Carex missouriensis\|STARR\|B460\|ILL\|spm00001067* | Non-Himalayan | 0 | 1 | | 0 | 1 | |
| *Carex mitchelliana\|STARR\|B495\|ALA\|spm00001101* | Non-Himalayan | 0 | 1 | | 0 | 1 | |
| *Carex mitrata\|XIAOFENG\|NA\|CHS\|spm00000085* | Non-Himalayan | 0 | 1 | | 0 | 1 | |
| *Carex molesta\|STARR\|B463\|DEL\|spm00001070* | Non-Himalayan | 0 | 1 | | 0 | 1 | |
| *Carex molestiformis\|STARR\|B466\|ARK\|spm00001073* | Non-Himalayan | 0 | 1 | | 0 | 1 | |
| *Carex monostachya\|GEHRKE\|NA\|KEN\|spm00000211* | Non-Himalayan | 0 | 1 | | 0 | 1 | |
| *Carex monotropa\|GEHRKE\|NA\|NAT\|spm00000215* | Non-Himalayan | 0 | 1 | | 0 | 1 | |
| *Carex montana\|LUCENO\|NA\|YUG\|spm00000543* | Non-Himalayan | 0 | 1 | | 0 | 1 | |
| *Carex montis-everesti\|JIMENEZMEJIAS\|\|CHT\|spm00003369* | Both | 1 | 1 | | 1 | 0 | |
| *Carex moorcroftii\|JIMENEZMEJIAS\|\|CHQ\|spm00003370* | Both | 1 | 1 | | 1 | 0 | |
| *Carex morii\|SANGTAE\|F0014\|KOR?\|spm00000340* | Non-Himalayan | 0 | 1 | | 0 | 1 | |
| *Carex mossii\|LUCENO\|27E1MMR11\|NAT\|spm00003760* | Non-Himalayan | 0 | 1 | | 0 | 1 | |
| *Carex moupinensis\|SHUREN\|C_moupinensis_BHY\|\|spm00003610* | Non-Himalayan | 0 | 1 | | 0 | 1 | |
| *Carex mucronata\|LUCENO\|NA\|ITA\|spm00000534* | Non-Himalayan | 0 | 1 | | 0 | 1 | |
| *Carex muehlenbergii\|HIPP\|muehlenbergii_3143\|WIS\|spm00002520* | Non-Himalayan | 0 | 1 | | 0 | 1 | |
| *Carex multicaulis\|STARR\|B486\|CAL\|spm00001092* | Non-Himalayan | 0 | 1 | | 0 | 1 | |
| *Carex multicostata\|STARR\|B487\|CAL\|spm00001093* | Non-Himalayan | 0 | 1 | | 0 | 1 | |
| *Carex multifaria\|STARR_FORD\|\|CLC\|spm00005101* | Non-Himalayan | 0 | 1 | | 0 | 1 | |
| *Carex munda\|JIMENEZMEJIAS\|\|EHM\|spm00003372* | Both | 1 | 1 | | 1 | 1 | |
| *Carex muriculata\|STARR\|B1203\|NWM\|spm00001810* | Non-Himalayan | 0 | 1 | | 0 | 1 | |
| *Carex muskingumensis\|STARR\|B509\|MIC\|spm00001115* | Non-Himalayan | 0 | 1 | | 0 | 1 | |
| *Carex myosuroides\|STARR_FORD\|STA_101\|FRA\|spm00005196* | Non-Himalayan | 0 | 1 | | 0 | 1 | |
| *Carex nakaoana\|JIMENEZMEJIAS\|\|CHT\|spm00003374* | Both | 1 | 1 | | 1 | 0 | |
| *Carex nakasimae\|SANGTAE\|E0483\|KOR?\|spm00000341* | Non-Himalayan | 0 | 1 | | 0 | 1 | |
| *Carex nardina\|STARR\|B909\|QUE\|spm00001510* | Non-Himalayan | 0 | 1 | | 0 | 1 | |
| *Carex nebraskensis\|STARR\|B510\|CAL\|spm00001116* | Non-Himalayan | 0 | 1 | | 0 | 1 | |
| *Carex neesii\|STARR_FORD\|STA_6\|EHM\|spm00005194* | Both | 1 | 1 | | 1 | 0 | |
| *Carex negrii\|JIMENEZMEJIAS\|\|SOM\|spm00003349* | Non-Himalayan | 0 | 1 | | 0 | 1 | |
| *Carex nelsonii\|STARR\|B1225\|MNT\|spm00001832* | Non-Himalayan | 0 | 1 | | 0 | 1 | |
| *Carex nemostachys\|XIAOFENG\|NA\|CHS\|spm00000088* | Non-Himalayan | 0 | 1 | | 0 | 1 | |
| *Carex nemurensis\|WATERWAY\|C.nemurensis_1963\|\|spm00004619* | Non-Himalayan | 0 | 1 | | 0 | 1 | |
| *Carex neochevalieri\|MESTERHAZY\|NA\|SIE\|spm00002179* | Non-Himalayan | 0 | 1 | | 0 | 1 | |
| *Carex nervata\|HIPP\|NA\|KOR\|spm00000010* | Non-Himalayan | 0 | 1 | | 0 | 1 | |
| *Carex nervina\|STARR\|B1233\|CAL\|spm00001840* | Non-Himalayan | 0 | 1 | | 0 | 1 | |
| *Carex neurocarpa\|HIPP\|neurocarpa_2736\|JAP\|spm00002534* | Non-Himalayan | 0 | 1 | | 0 | 1 | |
| *Carex neurophora\|STARR\|B1008\|WAS\|spm00001615* | Non-Himalayan | 0 | 1 | | 0 | 1 | |
| *Carex nigerrima\|JIMENEZMEJIAS\|NA\|PAK\|spm00003469* | Himalayan | 1 | 0 | | 1 | 0 | |
| *Carex nigra\|STARR\|B894\|NFL\|spm00001495* | Both | 1 | 1 | | 1 | 1 | |
| *Carex nigricans\|STARR\|B1031\|ARK\|spm00001638* | Non-Himalayan | 0 | 1 | | 0 | 1 | |
| *Carex nigromarginata\|STARR\|B515\|ARK\|spm00001121* | Non-Himalayan | 0 | 1 | | 0 | 1 | |
| *Carex nivalis\|JIMENEZMEJIAS\|\|AFG\|spm00003375* | Both | 1 | 1 | | 1 | 0 | |
| *Carex nodaeana\|JIMENEZMEJIAS\|NA\|CHM\|spm00003470* | Non-Himalayan | 0 | 1 | | 0 | 1 | |
| *Carex normalis\|STARR\|B451\|ALA\|spm00001059* | Non-Himalayan | 0 | 1 | | 0 | 1 | |
| *Carex norvegica\|STARR\|B1009\|ORE\|spm00001616* | Both | 1 | 1 | | 1 | 1 | |
| *Carex nova\|STARR\|B081\|COL\|spm00000700* | Non-Himalayan | 0 | 1 | | 0 | 1 | |
| *Carex novaeangliae\|STARR\|B453\|MAI\|spm00001061* | Non-Himalayan | 0 | 1 | | 0 | 1 | |
| *Carex nubigena\|PAK\|183085* | Both | 1 | 1 | | 1 | 1 | |
| *Carex nudata\|STARR\|B837\|CAL\|spm00001440* | Non-Himalayan | 0 | 1 | | 0 | 1 | |
| *Carex obispoensis\|STARR\|B1190\|CAL\|spm00001797* | Non-Himalayan | 0 | 1 | | 0 | 1 | |
| *Carex obliquicarpa\|XIAOFENG\|NA\|CHS\|spm00000089* | Non-Himalayan | 0 | 1 | | 0 | 1 | |
| *Carex obnupta\|STARR\|B499\|CAL\|spm00001105* | Non-Himalayan | 0 | 1 | | 0 | 1 | |
| *Carex obovatosquamata\|CHC\|E00666339* | Non-Himalayan | 0 | 1 | | 1 | 0 | |
| *Carex obscuriceps\|EHM\|E00693599* | Both | 1 | 1 | | 1 | 0 | |
| *Carex obtusata\|STARR\|B501\|MAN\|spm00001107* | Non-Himalayan | 0 | 1 | | 0 | 1 | |
| *Carex obtusifolia\|JIMENEZMEJIAS\|\|\|spm00004428* | Non-Himalayan | 0 | 1 | | 0 | 1 | |
| *Carex occidentalis\|REZNICEK\|NA\|MXC\|spm00000282* | Non-Himalayan | 0 | 1 | | 0 | 1 | |
| *Carex oedipostyla\|LUCENO\|NA\|SPA\|spm00000587* | Non-Himalayan | 0 | 1 | | 0 | 1 | |
| *Carex oklahomensis\|STARR\|B505\|ARK\|spm00001111* | Non-Himalayan | 0 | 1 | | 0 | 1 | |
| *Carex olbiensis\|LUCENO\|NA\|SPA\|spm00000586* | Non-Himalayan | 0 | 1 | | 0 | 1 | |
| *Carex oligocarpa\|STARR\|B931\|ARK\|spm00001532* | Non-Himalayan | 0 | 1 | | 0 | 1 | |
| *Carex oligocarya\|JIMENEZMEJIAS\|\|PAK\|spm00003395* | Himalayan | 1 | 0 | | 1 | 0 | |
| *Carex oligosperma\|STARR\|B506\|NWH\|spm00001112* | Non-Himalayan | 0 | 1 | | 0 | 1 | |
| *Carex opaca\|STARR\|B527\|OKL\|spm00001133* | Non-Himalayan | 0 | 1 | | 0 | 1 | |
| *Carex oreophila\|JIMENEZMEJIAS\|NA\|TCS\|spm00003471* | Non-Himalayan | 0 | 1 | | 0 | 1 | |
| *Carex orizabae\|REZNICEK\|NA\|MXC\|spm00000283* | Non-Himalayan | 0 | 1 | | 0 | 1 | |
| *Carex ormostachya\|STARR\|B528\|MIC\|spm00001134* | Non-Himalayan | 0 | 1 | | 0 | 1 | |
| *Carex ornithopoda\|LUCENO\|NA\|YUG\|spm00000560* | Non-Himalayan | 0 | 1 | | 0 | 1 | |
| *Carex oronensis\|STARR\|B1291\|MAI\|spm00001898* | Non-Himalayan | 0 | 1 | | 0 | 1 | |
| *Carex otrubae\|JIMENEZMEJIAS\|NA\|SPA\|spm00000559* | Non-Himalayan | 0 | 1 | | 1 | 1 | |
| *Carex ouachitana\|STARR\|B937\|WAS\|spm00001538* | Non-Himalayan | 0 | 1 | | 0 | 1 | |
| *Carex ovatispiculata\|CHT\|MSB-140867* | Non-Himalayan | 0 | 1 | | 1 | 1 | |
| *Carex ovoidispica\|JIMENEZMEJIAS\|\|PAK\|spm00003406* | Himalayan | 1 | 0 | | 1 | 0 | |
| *Carex oxylepis\|STARR\|B531\|KTY\|spm00001137* | Non-Himalayan | 0 | 1 | | 0 | 1 | |
| *Carex ozarkana\|HIPP\|ozarkana_9357\|ARK\|spm00002557* | Non-Himalayan | 0 | 1 | | 0 | 1 | |
| *Carex pachygyna\|WATERWAY\|pachygyna_1828\|JAP\|spm00002558* | Non-Himalayan | 0 | 1 | | 0 | 1 | |
| *Carex pachystachya\|STARR\|B1257\|ARK\|spm00001864* | Non-Himalayan | 0 | 1 | | 0 | 1 | |
| *Carex pachystylis\|JIMENEZMEJIAS\|NA\|UZB\|spm00003472* | Non-Himalayan | 0 | 1 | | 0 | 1 | |
| *Carex paeninsulae\|STARR\|B842\|FLA\|spm00001445* | Non-Himalayan | 0 | 1 | | 0 | 1 | |
| *Carex pairae\|JIMENEZMEJIAS\|\|SPA\|spm00004401* | Non-Himalayan | 0 | 1 | | 0 | 1 | |
| *Carex paleacea\|STARR\|B521\|MAI\|spm00001127* | Non-Himalayan | 0 | 1 | | 0 | 1 | |
| *Carex pallescens\|STARR\|B1401\|BRC\|spm00002008* | Non-Himalayan | 0 | 1 | | 0 | 1 | |
| *Carex pallidula\|JIMENEZMEJIAS\|\|FIN\|spm00004388* | Non-Himalayan | 0 | 1 | | 0 | 1 | |
| *Carex paneroi\|REZNICEK\|NA\|MXS\|spm00000363* | Non-Himalayan | 0 | 1 | | 0 | 1 | |
| *Carex panicea\|STARR\|B1299\|MAI\|spm00001906* | Non-Himalayan | 0 | 1 | | 0 | 1 | |
| *Carex paniculata\|MAGUILLA\|\|\|spm00006108* | Non-Himalayan | 0 | 1 | | 0 | 1 | |
| *Carex panormitana\|LUCENO\|22E2SMB13\|SAR\|spm00003779* | Non-Himalayan | 0 | 1 | | 0 | 1 | |
| *Carex pansa\|STARR\|B1357\|CAL\|spm00001964* | Non-Himalayan | 0 | 1 | | 0 | 1 | |
| *Carex papulosa\|HIPP\|NA\|KOR\|spm00000016* | Non-Himalayan | 0 | 1 | | 0 | 1 | |
| *Carex parallela\|JIMENEZMEJIAS\|\|FIN\|spm00003596* | Non-Himalayan | 0 | 1 | | 0 | 1 | |
| *Carex parryana\|STARR\|B1027\|ARK\|spm00001634* | Non-Himalayan | 0 | 1 | | 0 | 1 | |
| *Carex parva\|STARR_FORD\|\|CHC\|spm00005212* | Both | 1 | 1 | | 1 | 1 | |
| *Carex parviflora\|LUCENO\|NA\|SWI\|spm00000544* | Non-Himalayan | 0 | 1 | | 0 | 1 | |
| *Carex parvispica\|JIMENEZMEJIAS\|\|\|spm00004427* | Non-Himalayan | 0 | 1 | | 0 | 1 | |
| *Carex parvula\|SHUREN\|K_pygmaea_ZSR_K\|\|spm00003646* | Both | 1 | 1 | | 1 | 1 | |
| *Carex pauciflora\|STARR\|B536\|ARK\|spm00001142* | Non-Himalayan | 0 | 1 | | 0 | 1 | |
| *Carex paulovargasii\|JIMENEZMEJIAS\|\|MOR\|spm00004349* | Non-Himalayan | 0 | 1 | | 0 | 1 | |
| *Carex paysonis\|JIMENEZMEJIAS\|\|ORE\|spm00004437* | Non-Himalayan | 0 | 1 | | 0 | 1 | |
| *Carex peckii\|STARR\|B1018\|ARK\|spm00001625* | Non-Himalayan | 0 | 1 | | 0 | 1 | |
| *Carex pediformis\|JIMENEZMEJIAS\|\|CHC\|spm00003390* | Non-Himalayan | 0 | 1 | | 0 | 1 | |
| *Carex pedunculata\|STARR\|B541\|MAS\|spm00001147* | Non-Himalayan | 0 | 1 | | 0 | 1 | |
| *Carex peichuniana\|SHUREN\|K_inflata_LJQ\|\|spm00003638* | Both | 1 | 1 | | 1 | 0 | |
| *Carex pellita\|STARR\|B542\|CAL\|spm00001148* | Non-Himalayan | 0 | 1 | | 0 | 1 | |
| *Carex pelocarpa\|STARR\|B589\|NEV\|spm00001195* | Non-Himalayan | 0 | 1 | | 0 | 1 | |
| *Carex pendula\|STARR\|B591\|AUT\|spm00001197* | Non-Himalayan | 0 | 1 | | 0 | 1 | |
| *Carex pensylvanica\|STARR\|B565\|MAN\|spm00001171* | Non-Himalayan | 0 | 1 | | 0 | 1 | |
| *Carex perdentata\|STARR\|B585\|TEX\|spm00001191* | Non-Himalayan | 0 | 1 | | 0 | 1 | |
| *Carex peregrina\|LUCENO\|NA\|MDR\|spm00000564* | Non-Himalayan | 0 | 1 | | 0 | 1 | |
| *Carex perglobosa\|STARR\|B1196\|COL\|spm00001803* | Non-Himalayan | 0 | 1 | | 0 | 1 | |
| *Carex perplexa\|JIMENEZMEJIAS\|\|\|spm00004409* | Non-Himalayan | 0 | 1 | | 0 | 1 | |
| *Carex perraudieriana\|JIMENEZMEJIAS\|\|CNY\|spm00004346* | Non-Himalayan | 0 | 1 | | 0 | 1 | |
| *Carex perstricta\|REZNICEK\|NA\|MXS\|spm00000359* | Non-Himalayan | 0 | 1 | | 0 | 1 | |
| *Carex petasata\|STARR\|B586\|UTA\|spm00001192* | Non-Himalayan | 0 | 1 | | 0 | 1 | |
| *Carex petitiana\|GEHRKE\|NA\|MLW\|spm00000219* | Non-Himalayan | 0 | 1 | | 0 | 1 | |
| *Carex peucophila\|JIMENEZMEJIAS\|NA\|COS\|spm00000516* | Non-Himalayan | 0 | 1 | | 0 | 1 | |
| *Carex phaeocephala\|STARR\|B587\|NEV\|spm00001193* | Non-Himalayan | 0 | 1 | | 0 | 1 | |
| *Carex phleoides\|STARR_FORD\|\|CLC\|spm00005103* | Non-Himalayan | 0 | 1 | | 0 | 1 | |
| *Carex phragmitoides\|GEHRKE\|NA\|ETH\|spm00000221* | Non-Himalayan | 0 | 1 | | 0 | 1 | |
| *Carex phyllostachys\|STARR_FORD\|\|TUR\|spm00005164* | Non-Himalayan | 0 | 1 | | 0 | 1 | |
| *Carex physodes\|STARR_FORD\|STA_849\|TZK\|spm00005427* | Non-Himalayan | 0 | 1 | | 1 | 1 | |
| *Carex pichinchensis\|LUCENO\|NA\|ECU\|spm00000538* | Non-Himalayan | 0 | 1 | | 0 | 1 | |
| *Carex picta\|STARR\|B553\|GEO\|spm00001159* | Non-Himalayan | 0 | 1 | | 0 | 1 | |
| *Carex pigra\|STARR\|B1178\|MSI\|spm00001785* | Non-Himalayan | 0 | 1 | | 0 | 1 | |
| *Carex pilosa\|LUCENO\|NA\|YUG\|spm00000563* | Non-Himalayan | 0 | 1 | | 0 | 1 | |
| *Carex pilulifera\|LUCENO\|NA\|POR\|spm00000583* | Non-Himalayan | 0 | 1 | | 0 | 1 | |
| *Carex pinophila\|REZNICEK\|NA\|MXE\|spm00000284* | Non-Himalayan | 0 | 1 | | 0 | 1 | |
| *Carex pisiformis\|XIAOFENG\|NA\|CHS\|spm00000095* | Non-Himalayan | 0 | 1 | | 0 | 1 | |
| *Carex pityophila\|STARR\|B1223\|NWM\|spm00001830* | Non-Himalayan | 0 | 1 | | 0 | 1 | |
| *Carex planata\|STARR_FORD\|STA_850\|JAP\|spm00005429* | Non-Himalayan | 0 | 1 | | 0 | 1 | |
| *Carex planilomina\|REZNICEK\|NA\|MXE\|spm00000365* | Non-Himalayan | 0 | 1 | | 0 | 1 | |
| *Carex planispicata\|STARR\|B047\|MRY\|spm00000666* | Non-Himalayan | 0 | 1 | | 0 | 1 | |
| *Carex planostachys\|STARR\|B544\|TEX\|spm00001150* | Non-Himalayan | 0 | 1 | | 0 | 1 | |
| *Carex plantaginea\|STARR\|B1286\|TEN\|spm00001893* | Non-Himalayan | 0 | 1 | | 0 | 1 | |
| *Carex platyphylla\|STARR\|B546\|MRY\|spm00001152* | Non-Himalayan | 0 | 1 | | 0 | 1 | |
| *Carex plectobasis\|JIMENEZMEJIAS\|\|CHT\|spm00003378* | Both | 1 | 1 | | 1 | 0 | |
| *Carex pleiostachys\|JIMENEZMEJIAS\|\|\|spm00004408* | Non-Himalayan | 0 | 1 | | 0 | 1 | |
| *Carex pluriflora\|STARR\|B1349\|ARK\|spm00001956* | Non-Himalayan | 0 | 1 | | 0 | 1 | |
| *Carex podocarpa\|STARR\|B1325\|BRC\|spm00001932* | Non-Himalayan | 0 | 1 | | 0 | 1 | |
| *Carex polymorpha\|STARR\|B1300\|MAI\|spm00001907* | Non-Himalayan | 0 | 1 | | 0 | 1 | |
| *Carex polyschoenoides\|XIAOFENG\|NA\|CHN\|spm00000096* | Non-Himalayan | 0 | 1 | | 0 | 1 | |
| *Carex polystachya\|STARR_FORD\|STA_210\|BLZ\|spm00005148* | Non-Himalayan | 0 | 1 | | 0 | 1 | |
| *Carex polysticha\|REZNICEK\|NA\|DOM\|spm00000292* | Non-Himalayan | 0 | 1 | | 0 | 1 | |
| *Carex pomiensis\|JIMENEZMEJIAS\|\|CHT\|spm00003352* | Both | 1 | 1 | | 1 | 0 | |
| *Carex potens\|JIMENEZMEJIAS\|\|\|spm00004430* | Non-Himalayan | 0 | 1 | | 0 | 1 | |
| *Carex potosina\|HIPP\|potosina_sn\|MXE\|spm00002588* | Non-Himalayan | 0 | 1 | | 0 | 1 | |
| *Carex praeceptorium\|STARR\|B1345\|CAL\|spm00001952* | Non-Himalayan | 0 | 1 | | 0 | 1 | |
| *Carex praeclara\|JIMENEZMEJIAS\|\|CHT\|spm00003379* | Himalayan | 1 | 0 | | 1 | 0 | |
| *Carex praecox\|MESTERHAZY\|NA\|HUN\|spm00002176* | Non-Himalayan | 0 | 1 | | 0 | 1 | |
| *Carex praegracilis\|STARR\|B551\|CAL\|spm00001157* | Non-Himalayan | 0 | 1 | | 0 | 1 | |
| *Carex prainii\|EHM\|E00256786* | Both | 1 | 1 | | 1 | 0 | |
| *Carex prairea\|STARR\|B1344\|BRC\|spm00001951* | Non-Himalayan | 0 | 1 | | 0 | 1 | |
| *Carex prasina\|STARR\|B560\|GEO\|spm00001166* | Non-Himalayan | 0 | 1 | | 0 | 1 | |
| *Carex praticola\|STARR\|B557\|COL\|spm00001163* | Non-Himalayan | 0 | 1 | | 0 | 1 | |
| *Carex preslii\|STARR\|B568\|ORE\|spm00001174* | Non-Himalayan | 0 | 1 | | 0 | 1 | |
| *Carex pringlei\|REZNICEK\|NA\|MXE\|spm00000293* | Non-Himalayan | 0 | 1 | | 0 | 1 | |
| *Carex projecta\|STARR\|B570\|MAN\|spm00001176* | Non-Himalayan | 0 | 1 | | 0 | 1 | |
| *Carex proposita\|STARR\|B1334\|CAL\|spm00001941* | Non-Himalayan | 0 | 1 | | 0 | 1 | |
| *Carex proxima\|GEHRKE\|NA\|MDG\|spm00000222* | Non-Himalayan | 0 | 1 | | 0 | 1 | |
| *Carex pruinosa\|CHC\|E00263657* | Both | 1 | 1 | | 1 | 1 | |
| *Carex pseudobicolor\|JIMENEZMEJIAS\|\|PAK\|spm00003380* | Both | 1 | 1 | | 1 | 0 | |
| *Carex pseudobrizoides\|MESTERHAZY\|NA\|POL\|spm00002177* | Non-Himalayan | 0 | 1 | | 0 | 1 | |
| *Carex pseudochinensis\|SANGTAE\|E0479\|KOR?\|spm00000346* | Non-Himalayan | 0 | 1 | | 0 | 1 | |
| *Carex pseudocuraica\|HIPP\|pseudocuraica_668\|CH?\|spm00002605* | Non-Himalayan | 0 | 1 | | 0 | 1 | |
| *Carex pseudocyperus\|STARR\|B573\|MIC\|spm00001179* | Both | 1 | 1 | | 1 | 1 | |
| *Carex pseudofoetida\|PAK\|183071* | Both | 1 | 1 | | 1 | 1 | |
| *Carex pseudogammiei\|SHUREN\|K_loliacea_LJQ\|\|spm00003639* | Both | 1 | 1 | | 1 | 0 | |
| *Carex pseudolaxa\|PAK\|183107* | Both | 1 | 1 | | 1 | 0 | |
| *Carex pseudololiacea\|MAGUILLA\|\|\|spm00006080* | Non-Himalayan | 0 | 1 | | 0 | 1 | |
| *Carex pseudorufa\|LUCENO\|\|\|spm00003250* | Non-Himalayan | 0 | 1 | | 0 | 1 | |
| *Carex pseudotristachya\|XIAOFENG\|NA\|CHS\|spm00000098* | Non-Himalayan | 0 | 1 | | 0 | 1 | |
| *Carex pseuduncinoides\|SHUREN\|K_kansuensis_ZSR_K\|\|spm00003619* | Both | 1 | 1 | | 1 | 1 | |
| *Carex pterocarpa\|JIMENEZMEJIAS\|\|\|spm00004425* | Non-Himalayan | 0 | 1 | | 0 | 1 | |
| *Carex pulchra\|EHM\|E00693608* | Both | 1 | 1 | | 1 | 0 | |
| *Carex pulicaris\|STARR_FORD\|\|GRB\|spm00005180* | Non-Himalayan | 0 | 1 | | 0 | 1 | |
| *Carex pumila\|STARR\|B1461\|NCA\|spm00002067* | Non-Himalayan | 0 | 1 | | 0 | 1 | |
| *Carex punctata\|JIMENEZMEJIAS\|\|MOR\|spm00004350* | Non-Himalayan | 0 | 1 | | 0 | 1 | |
| *Carex punicea\|STARR_FORD\|STA_72\|NZS\|spm00005105* | Non-Himalayan | 0 | 1 | | 0 | 1 | |
| *Carex purpurata\|JIMENEZMEJIAS\|\|\|spm00004431* | Non-Himalayan | 0 | 1 | | 0 | 1 | |
| *Carex purpurifera\|STARR\|B867\|KTY\|spm00001470* | Non-Himalayan | 0 | 1 | | 0 | 1 | |
| *Carex pycnostachys\|JIMENEZMEJIAS\|\|AFG\|spm00003389* | Non-Himalayan | 0 | 1 | | 0 | 1 | |
| *Carex pyramidalis\|GEHRKE\|NA\|MDG\|spm00000223* | Non-Himalayan | 0 | 1 | | 0 | 1 | |
| *Carex pyrenaica\|LUCENO\|NA\|SPA\|spm00000582* | Non-Himalayan | 0 | 1 | | 0 | 1 | |
| *Carex qiyunensis\|XIAOFENG\|NA\|CHS\|spm00000102* | Non-Himalayan | 0 | 1 | | 0 | 1 | |
| *Carex quadriflora\|SANGTAE\|E0482\|KOR?\|spm00000347* | Non-Himalayan | 0 | 1 | | 0 | 1 | |
| *Carex queretarensis\|REZNICEK\|NA\|MXE\|spm00000294* | Non-Himalayan | 0 | 1 | | 0 | 1 | |
| *Carex raddei\|HIPP\|NA\|KOR\|spm00000007* | Non-Himalayan | 0 | 1 | | 0 | 1 | |
| *Carex radfordii\|STARR\|B835\|SCA\|spm00001438* | Non-Himalayan | 0 | 1 | | 0 | 1 | |
| *Carex radiata\|STARR\|B592\|MIC\|spm00001198* | Non-Himalayan | 0 | 1 | | 0 | 1 | |
| *Carex radicalis\|EHM\|E00693596* | Both | 1 | 1 | | 1 | 0 | |
| *Carex rainbowii\|LUCENO\|\|NAT\|spm00003255* | Non-Himalayan | 0 | 1 | | 0 | 1 | |
| *Carex ramenskii\|STARR\|B836\|ARK\|spm00001439* | Non-Himalayan | 0 | 1 | | 0 | 1 | |
| *Carex ramosa\|HIPP\|NA\|REU\|spm00002208* | Non-Himalayan | 0 | 1 | | 0 | 1 | |
| *Carex randalpina\|LUCENO\|\|AUT\|spm00003098* | Non-Himalayan | 0 | 1 | | 0 | 1 | |
| *Carex rariflora\|STARR\|B1392\|YUK\|spm00001999* | Non-Himalayan | 0 | 1 | | 0 | 1 | |
| *Carex raynoldsii\|STARR\|B578\|CAL\|spm00001184* | Non-Himalayan | 0 | 1 | | 0 | 1 | |
| *Carex remota\|MAGUILLA\|\|\|spm00006117* | Both | 1 | 1 | | 1 | 1 | |
| *Carex remotiuscula\|HIPP\|remotiuscula_431\|CHC\|spm00002615* | Non-Himalayan | 0 | 1 | | 1 | 1 | |
| *Carex renauldii\|GEHRKE\|NA\|MDG\|spm00000224* | Non-Himalayan | 0 | 1 | | 0 | 1 | |
| *Carex reniformis\|STARR\|B581\|GEO\|spm00001187* | Non-Himalayan | 0 | 1 | | 0 | 1 | |
| *Carex renschiana\|HIPP\|NA\|MDG\|spm00002207* | Non-Himalayan | 0 | 1 | | 0 | 1 | |
| *Carex resectans\|WATERWAY\|resectans_1644\|NZN\|spm00002618* | Non-Himalayan | 0 | 1 | | 0 | 1 | |
| *Carex retroflexa\|STARR\|B584\|DEL\|spm00001190* | Non-Himalayan | 0 | 1 | | 0 | 1 | |
| *Carex retrorsa\|STARR\|B603\|MIC\|spm00001210* | Non-Himalayan | 0 | 1 | | 0 | 1 | |
| *Carex reznicekii\|STARR\|B605\|ALA\|spm00001212* | Non-Himalayan | 0 | 1 | | 0 | 1 | |
| *Carex rhizina\|MESTERHAZY\|NA\|AUT\|spm00002182* | Non-Himalayan | 0 | 1 | | 0 | 1 | |
| *Carex rhodesiaca\|GEHRKE\|Carex MOR_318\|\|spm00004696* | Non-Himalayan | 0 | 1 | | 0 | 1 | |
| *Carex rhynchoperigynium\|REZNICEK\|NA\|MXE\|spm00000295* | Non-Himalayan | 0 | 1 | | 0 | 1 | |
| *Carex richardsonii\|STARR\|B613\|MRY\|spm00001220* | Non-Himalayan | 0 | 1 | | 0 | 1 | |
| *Carex riloensis\|JIMENEZMEJIAS\|\|BUL\|spm00003599* | Non-Himalayan | 0 | 1 | | 0 | 1 | |
| *Carex rivulorum\|SHUREN\|C_hangzhouensis_BHY\|\|spm00003604* | Non-Himalayan | 0 | 1 | | 0 | 1 | |
| *Carex roanensis\|STARR\|B617\|NCA\|spm00001224* | Non-Himalayan | 0 | 1 | | 0 | 1 | |
| *Carex rochebrunii\|STARR_FORD\|\|CHS\|spm00005431* | Both | 1 | 1 | | 1 | 1 | |
| *Carex rorulenta\|JIMENEZMEJIAS\|NA\|BAL\|spm00003474* | Non-Himalayan | 0 | 1 | | 0 | 1 | |
| *Carex rosea\|STARR\|B607\|MAI\|spm00001214* | Non-Himalayan | 0 | 1 | | 0 | 1 | |
| *Carex rossii\|STARR\|B595\|CAL\|spm00001201* | Non-Himalayan | 0 | 1 | | 0 | 1 | |
| *Carex rostrata\|STARR\|B993\|WAS\|spm00001600* | Both | 1 | 1 | | 1 | 1 | |
| *Carex rotundata\|STARR\|B1142\|MAN\|spm00001749* | Non-Himalayan | 0 | 1 | | 0 | 1 | |
| *Carex rubicunda\|JIMENEZMEJIAS\|\|\|spm00004423* | Non-Himalayan | 0 | 1 | | 0 | 1 | |
| *Carex rufulistolon\|EHM\|E00424592* | Himalayan | 1 | 0 | | 1 | 0 | |
| *Carex rugulosa\|SANGTAE\|E0485\|KOR?\|spm00000348* | Non-Himalayan | 0 | 1 | | 0 | 1 | |
| *Carex runssoroensis\|GEHRKE\|NA\|UGA\|spm00000227* | Non-Himalayan | 0 | 1 | | 0 | 1 | |
| *Carex rupestris\|STARR_FORD\|STA_102\|FRA\|spm00005213* | Non-Himalayan | 0 | 1 | | 0 | 1 | |
| *Carex rutenbergiana\|GEHRKE\|NA\|MDG\|spm00000228* | Non-Himalayan | 0 | 1 | | 0 | 1 | |
| *Carex ruthii\|STARR\|B600a\|NCA\|spm00001206* | Non-Himalayan | 0 | 1 | | 0 | 1 | |
| *Carex sabulosa\|STARR\|B1039\|YUK\|spm00001646* | Non-Himalayan | 0 | 1 | | 0 | 1 | |
| *Carex sagei\|LUCENO\|NA\|AGS\|spm00000548* | Non-Himalayan | 0 | 1 | | 0 | 1 | |
| *Carex salina\|STARR\|B1346\|QUE\|spm00001953* | Non-Himalayan | 0 | 1 | | 0 | 1 | |
| *Carex salticola\|LUCENO\|NA\|AGS\|spm00000581* | Non-Himalayan | 0 | 1 | | 0 | 1 | |
| *Carex sanguinea\|PAK\|183075* | Both | 1 | 1 | | 1 | 0 | |
| *Carex sargentiana\|JIMENEZMEJIAS\|\|CHT\|spm00003408* | Non-Himalayan | 0 | 1 | | 1 | 1 | |
| *Carex sartwelliana\|STARR\|B1191\|CAL\|spm00001798* | Non-Himalayan | 0 | 1 | | 0 | 1 | |
| *Carex sartwellii\|STARR\|B1375\|BRC\|spm00001982* | Non-Himalayan | 0 | 1 | | 0 | 1 | |
| *Carex saxatilis\|STARR\|B616\|UTA\|spm00001223* | Non-Himalayan | 0 | 1 | | 0 | 1 | |
| *Carex saximontana\|STARR\|B1120\|COL\|spm00001727* | Non-Himalayan | 0 | 1 | | 0 | 1 | |
| *Carex scabrata\|STARR\|B636\|GEO\|spm00001243* | Non-Himalayan | 0 | 1 | | 0 | 1 | |
| *Carex scabriuscula\|STARR\|B639\|CAL\|spm00001246* | Non-Himalayan | 0 | 1 | | 0 | 1 | |
| *Carex scaposa\|SHUREN\|C_scaposa_BHY\|\|spm00003611* | Non-Himalayan | 0 | 1 | | 0 | 1 | |
| *Carex schiedeana\|REZNICEK\|NA\|MXG\|spm00000354* | Non-Himalayan | 0 | 1 | | 0 | 1 | |
| *Carex schimperiana\|LUCENO\|\|\|spm00003249* | Non-Himalayan | 0 | 1 | | 0 | 1 | |
| *Carex schlagintweitiana\|PAK\|183068* | Both | 1 | 1 | | 1 | 0 | |
| *Carex schottii\|STARR\|B1074\|CAL\|spm00001681* | Non-Himalayan | 0 | 1 | | 0 | 1 | |
| *Carex schweickerdtii\|GEHRKE\|NA\|NAT\|spm00000160* | Non-Himalayan | 0 | 1 | | 0 | 1 | |
| *Carex schweinitzii\|STARR\|B649\|MIC\|spm00001256* | Non-Himalayan | 0 | 1 | | 0 | 1 | |
| *Carex scirpoidea\|STARR_FORD\|STA_180\|ABT\|spm00005153* | Non-Himalayan | 0 | 1 | | 0 | 1 | |
| *Carex senanensis\|STARR_FORD\|STA_863\|MAN\|spm00005371* | Non-Himalayan | 0 | 1 | | 0 | 1 | |
| *Carex senta\|STARR\|B1278\|ARI\|spm00001885* | Non-Himalayan | 0 | 1 | | 0 | 1 | |
| *Carex seorsa\|STARR\|B635\|DEL\|spm00001242* | Non-Himalayan | 0 | 1 | | 0 | 1 | |
| *Carex serpenticola\|STARR\|B1354\|CAL\|spm00001961* | Non-Himalayan | 0 | 1 | | 0 | 1 | |
| *Carex serratodens\|STARR\|B619\|CAL\|spm00001226* | Non-Himalayan | 0 | 1 | | 0 | 1 | |
| *Carex setschwanensis\|SHUREN\|K_setschwanensis_ZSR_K\|\|spm00003620* | Non-Himalayan | 0 | 1 | | 1 | 1 | |
| *Carex shangchengensis\|XIAOFENG\|NA\|CHS\|spm00000071* | Non-Himalayan | 0 | 1 | | 0 | 1 | |
| *Carex sheldonii\|STARR\|B1086\|CAL\|spm00001693* | Non-Himalayan | 0 | 1 | | 0 | 1 | |
| *Carex shinnersii\|STARR\|B618\|TEN\|spm00001225* | Non-Himalayan | 0 | 1 | | 0 | 1 | |
| *Carex shortiana\|STARR\|B622\|KTY\|spm00001229* | Non-Himalayan | 0 | 1 | | 0 | 1 | |
| *Carex siccata\|STARR\|B1464\|ONT\|spm00002070* | Non-Himalayan | 0 | 1 | | 0 | 1 | |
| *Carex siderosticta\|WATERWAY\|siderosticta_1837\|JAP\|spm00002643* | Non-Himalayan | 0 | 1 | | 0 | 1 | |
| *Carex silicea\|STARR\|B625\|DEL\|spm00001232* | Non-Himalayan | 0 | 1 | | 0 | 1 | |
| *Carex silvestris\|JIMENEZMEJIAS\|\|\|spm00004421* | Non-Himalayan | 0 | 1 | | 0 | 1 | |
| *Carex simensis\|JIMENEZMEJIAS\|\|KEN\|spm00004374* | Non-Himalayan | 0 | 1 | | 0 | 1 | |
| *Carex simpliciuscula\|JIMENEZMEJIAS\|\|CHT\|spm00003402* | Non-Himalayan | 0 | 1 | | 1 | 1 | |
| *Carex simulans\|SHUREN\|C_simulans_BHY\|\|spm00003605* | Non-Himalayan | 0 | 1 | | 0 | 1 | |
| *Carex simulata\|STARR\|B678\|CAL\|spm00001284* | Non-Himalayan | 0 | 1 | | 0 | 1 | |
| *Carex socialis\|STARR\|B682\|ALA\|spm00001288* | Non-Himalayan | 0 | 1 | | 0 | 1 | |
| *Carex songorica\|JIMENEZMEJIAS\|\|TZK\|spm00003384* | Both | 1 | 1 | | 1 | 1 | |
| *Carex sorianoi\|LUCENO\|NA\|AGS\|spm00000542* | Non-Himalayan | 0 | 1 | | 0 | 1 | |
| *Carex sororia\|STARR_FORD\|STA_868\|PAR\|spm00005362* | Non-Himalayan | 0 | 1 | | 0 | 1 | |
| *Carex sparganioides\|STARR\|B683\|DEL\|spm00001289* | Non-Himalayan | 0 | 1 | | 0 | 1 | |
| *Carex spartea\|STARR_FORD\|STA_50\|OFS\|spm00005206* | Non-Himalayan | 0 | 1 | | 0 | 1 | |
| *Carex specifica\|STARR\|B686\|CAL\|spm00001292* | Non-Himalayan | 0 | 1 | | 0 | 1 | |
| *Carex spectabilis\|STARR\|B1372\|BRC\|spm00001979* | Non-Himalayan | 0 | 1 | | 0 | 1 | |
| *Carex specuicola\|STARR\|B1207\|ARI\|spm00001814* | Non-Himalayan | 0 | 1 | | 0 | 1 | |
| *Carex sphaerogyna\|GEHRKE\|NA\|MDG\|spm00000237* | Non-Himalayan | 0 | 1 | | 0 | 1 | |
| *Carex spicata\|STARR\|B666\|BUL\|spm00001272* | Non-Himalayan | 0 | 1 | | 0 | 1 | |
| *Carex spissa\|REZNICEK\|NA\|MXE\|spm00000296* | Non-Himalayan | 0 | 1 | | 0 | 1 | |
| *Carex sprengelii\|STARR\|B1159\|NBR\|spm00001766* | Non-Himalayan | 0 | 1 | | 0 | 1 | |
| *Carex squarrosa\|STARR\|B675\|ARK\|spm00001281* | Non-Himalayan | 0 | 1 | | 0 | 1 | |
| *Carex stellata\|REZNICEK\|NA\|MXE\|spm00000360* | Non-Himalayan | 0 | 1 | | 0 | 1 | |
| *Carex stenocarpa\|JIMENEZMEJIAS\|\|CHC\|spm00003385* | Both | 1 | 1 | | 1 | 1 | |
| *Carex stenophylla\|JIMENEZMEJIAS\|\|TCS\|spm00003603* | Both | 1 | 1 | | 1 | 1 | |
| *Carex stenoptila\|STARR\|B676\|UTA\|spm00001282* | Non-Himalayan | 0 | 1 | | 0 | 1 | |
| *Carex sterilis\|STARR\|B1136\|MAN\|spm00001743* | Non-Himalayan | 0 | 1 | | 0 | 1 | |
| *Carex steudneri\|GEHRKE\|Carex MOR_909\|\|spm00004702* | Non-Himalayan | 0 | 1 | | 0 | 1 | |
| *Carex stevenii\|STARR\|B1103\|COL\|spm00001710* | Non-Himalayan | 0 | 1 | | 0 | 1 | |
| *Carex stipata\|STARR_FORD\|STA_873\|MRY\|spm00005444* | Non-Himalayan | 0 | 1 | | 0 | 1 | |
| *Carex straminea\|STARR\|B645\|DEL\|spm00001252* | Non-Himalayan | 0 | 1 | | 0 | 1 | |
| *Carex straminiformis\|STARR\|B660\|UTA\|spm00001266* | Non-Himalayan | 0 | 1 | | 0 | 1 | |
| *Carex striata\|STARR\|B668\|FLA\|spm00001274* | Non-Himalayan | 0 | 1 | | 0 | 1 | |
| *Carex striatula\|STARR\|B658\|DEL\|spm00001264* | Non-Himalayan | 0 | 1 | | 0 | 1 | |
| *Carex stricta\|STARR\|B690\|TEX\|spm00001296* | Non-Himalayan | 0 | 1 | | 0 | 1 | |
| *Carex strictissima\|JIMENEZMEJIAS\|\|NZS\|spm00004413* | Non-Himalayan | 0 | 1 | | 0 | 1 | |
| *Carex strigosa\|LUCENO\|\|\|spm00003302* | Non-Himalayan | 0 | 1 | | 0 | 1 | |
| *Carex styloflexa\|STARR\|B693\|GEO\|spm00001299* | Non-Himalayan | 0 | 1 | | 0 | 1 | |
| *Carex stylosa\|STARR\|B889\|NFL\|spm00001490* | Non-Himalayan | 0 | 1 | | 0 | 1 | |
| *Carex subbracteata\|STARR\|B1348\|CAL\|spm00001955* | Non-Himalayan | 0 | 1 | | 0 | 1 | |
| *Carex subebracteata\|XIAOFENG\|NA\|CHI\|spm00000121* | Non-Himalayan | 0 | 1 | | 0 | 1 | |
| *Carex suberecta\|STARR\|B953\|ARK\|spm00001559* | Non-Himalayan | 0 | 1 | | 0 | 1 | |
| *Carex subfusca\|HIPP\|NA\|ARI\|spm00000307* | Non-Himalayan | 0 | 1 | | 0 | 1 | |
| *Carex subnigricans\|STARR\|B1067\|ORE\|spm00001674* | Non-Himalayan | 0 | 1 | | 0 | 1 | |
| *Carex subphysodes\|JIMENEZMEJIAS\|NA\|AFG\|spm00003477* | Non-Himalayan | 0 | 1 | | 0 | 1 | |
| *Carex subsacculata\|STARR_FORD\|STA_147\|ECU\|spm00005108* | Non-Himalayan | 0 | 1 | | 0 | 1 | |
| *Carex subspathacea\|STARR\|B1040\|ARK\|spm00001647* | Non-Himalayan | 0 | 1 | | 0 | 1 | |
| *Carex subtilis\|JIMENEZMEJIAS\|\|\|spm00004415* | Non-Himalayan | 0 | 1 | | 0 | 1 | |
| *Carex subviridis\|JIMENEZMEJIAS\|\|\|spm00004429* | Non-Himalayan | 0 | 1 | | 0 | 1 | |
| *Carex suifunensis\|SANGTAE\|E0525\|KOR?\|spm00000350* | Non-Himalayan | 0 | 1 | | 0 | 1 | |
| *Carex superata\|STARR\|B012\|KTY\|spm00000632* | Non-Himalayan | 0 | 1 | | 0 | 1 | |
| *Carex swanii\|STARR\|B699\|ARK\|spm00001304* | Non-Himalayan | 0 | 1 | | 0 | 1 | |
| *Carex sychnocephala\|STARR\|B1293\|ONT\|spm00001900* | Non-Himalayan | 0 | 1 | | 0 | 1 | |
| *Carex sylvatica\|STARR\|B983\|WAS\|spm00001590* | Non-Himalayan | 0 | 1 | | 0 | 1 | |
| *Carex tahoensis\|STARR\|B1068\|ORE\|spm00001675* | Non-Himalayan | 0 | 1 | | 0 | 1 | |
| *Carex tangulashanensis\|JIMENEZMEJIAS\|\|CHQ\|spm00003591* | Non-Himalayan | 0 | 1 | | 1 | 1 | |
| *Carex tapintzensis\|JIMENEZMEJIAS\|\|CHT\|spm00003393* | Non-Himalayan | 0 | 1 | | 1 | 0 | |
| *Carex tasmanica\|JIMENEZMEJIAS\|\|TAS\|spm00004362* | Non-Himalayan | 0 | 1 | | 0 | 1 | |
| *Carex tenax\|STARR\|B703\|LOU\|spm00001308* | Non-Himalayan | 0 | 1 | | 0 | 1 | |
| *Carex tenera\|HIPP\|tenera_3124\|WIS\|spm00002701* | Non-Himalayan | 0 | 1 | | 0 | 1 | |
| *Carex teneriformis\|HIPP\|teneraeformis_716\|CAL\|spm00002704* | Non-Himalayan | 0 | 1 | | 0 | 1 | |
| *Carex tenuiflora\|STARR\|B708\|MAN\|spm00001313* | Non-Himalayan | 0 | 1 | | 0 | 1 | |
| *Carex tetanica\|STARR\|B709\|MAN\|spm00001314* | Non-Himalayan | 0 | 1 | | 0 | 1 | |
| *Carex tetrastachya\|STARR\|B663\|TEX\|spm00001269* | Non-Himalayan | 0 | 1 | | 0 | 1 | |
| *Carex texensis\|STARR_FORD\|\|TEN\|spm00005419* | Non-Himalayan | 0 | 1 | | 0 | 1 | |
| *Carex thornei\|STARR\|B040\|FLA\|spm00000659* | Non-Himalayan | 0 | 1 | | 0 | 1 | |
| *Carex thurberi\|STARR\|B1209\|ARI\|spm00001816* | Non-Himalayan | 0 | 1 | | 0 | 1 | |
| *Carex tibetikobresia\|SHUREN\|K_capilifolia_ZSR_K\|\|spm00003617* | Non-Himalayan | 0 | 1 | | 1 | 1 | |
| *Carex timida\|STARR\|B005\|ALA\|spm00000625* | Non-Himalayan | 0 | 1 | | 0 | 1 | |
| *Carex tincta\|STARR\|B737\|MAI\|spm00001342* | Non-Himalayan | 0 | 1 | | 0 | 1 | |
| *Carex tomentosa\|LUCENO\|NA\|SPA\|spm00000568* | Non-Himalayan | 0 | 1 | | 0 | 1 | |
| *Carex tonsa\|STARR\|B712\|\|spm00001317* | Non-Himalayan | 0 | 1 | | 0 | 1 | |
| *Carex torreyi\|STARR\|B1101\|COL\|spm00001708* | Non-Himalayan | 0 | 1 | | 0 | 1 | |
| *Carex torta\|STARR\|B715\|PEN\|spm00001320* | Non-Himalayan | 0 | 1 | | 0 | 1 | |
| *Carex trachycarpa\|WATERWAY\|trachycarpa_3523\|\|spm00002713* | Non-Himalayan | 0 | 1 | | 0 | 1 | |
| *Carex traiziscana\|MAGUILLA\|\|\|spm00004625* | Non-Himalayan | 0 | 1 | | 0 | 1 | |
| *Carex transandina\|LUCENO\|NA\|AGS\|spm00000554* | Non-Himalayan | 0 | 1 | | 0 | 1 | |
| *Carex transcaucasica\|LUCENO\|\|Iran\|spm00003280* | Non-Himalayan | 0 | 1 | | 0 | 1 | |
| *Carex traversii\|K_FORD\|NA\|NZS\|spm00002187* | Non-Himalayan | 0 | 1 | | 0 | 1 | |
| *Carex triangula\|STARR_FORD\|\|AGS\|spm00005218* | Non-Himalayan | 0 | 1 | | 0 | 1 | |
| *Carex triangularis\|STARR\|B739\|TEX\|spm00001344* | Non-Himalayan | 0 | 1 | | 0 | 1 | |
| *Carex trichocarpa\|STARR\|B726\|DEL\|spm00001331* | Non-Himalayan | 0 | 1 | | 0 | 1 | |
| *Carex tricolor\|JIMENEZMEJIAS\|\|BUL\|spm00003600* | Non-Himalayan | 0 | 1 | | 0 | 1 | |
| *Carex trinervis\|LUCENO\|42E10NPJM\|SPA\|spm00003778* | Non-Himalayan | 0 | 1 | | 0 | 1 | |
| *Carex triquetra\|STARR\|B1193\|CAL\|spm00001800* | Non-Himalayan | 0 | 1 | | 0 | 1 | |
| *Carex trisperma\|STARR\|B729\|MIC\|spm00001334* | Non-Himalayan | 0 | 1 | | 0 | 1 | |
| *Carex tristachya\|XIAOFENG\|NA\|CHS\|spm00000132* | Non-Himalayan | 0 | 1 | | 0 | 1 | |
| *Carex tristis\|JIMENEZMEJIAS\|NA\|IRN\|spm00000599* | Non-Himalayan | 0 | 1 | | 0 | 1 | |
| *Carex troodi\|JIMENEZMEJIAS\|\|CYP\|spm00004356* | Non-Himalayan | 0 | 1 | | 0 | 1 | |
| *Carex tsushimensis\|SANGTAE\|E0541\|KOR?\|spm00000353* | Non-Himalayan | 0 | 1 | | 0 | 1 | |
| *Carex tuberculata\|REZNICEK\|NA\|MXC\|spm00000300* | Non-Himalayan | 0 | 1 | | 0 | 1 | |
| *Carex tuckermanii\|STARR\|B740\|MIC\|spm00001345* | Non-Himalayan | 0 | 1 | | 0 | 1 | |
| *Carex tumulicola\|STARR\|B733\|CAL\|spm00001338* | Non-Himalayan | 0 | 1 | | 0 | 1 | |
| *Carex tunimanensis\|REZNICEK\|NA\|MXT\|spm00000303* | Non-Himalayan | 0 | 1 | | 0 | 1 | |
| *Carex turbinata\|STARR\|B1270\|ARI\|spm00001877* | Non-Himalayan | 0 | 1 | | 0 | 1 | |
| *Carex turgescens\|STARR\|B839\|MSI\|spm00001442* | Non-Himalayan | 0 | 1 | | 0 | 1 | |
| *Carex turkestanica\|JIMENEZMEJIAS\|NA\|KGZ\|spm00003476* | Both | 1 | 1 | | 1 | 1 | |
| *Carex typhina\|STARR\|B725\|LOU\|spm00001330* | Non-Himalayan | 0 | 1 | | 0 | 1 | |
| *Carex uhligii\|STARR_FORD\|STA_54\|NAT\|spm00005205* | Non-Himalayan | 0 | 1 | | 0 | 1 | |
| *Carex ulobasis\|HIPP\|NA\|KOR\|spm00000003* | Non-Himalayan | 0 | 1 | | 0 | 1 | |
| *Carex umbellata\|STARR\|B771\|DEL\|spm00001376* | Non-Himalayan | 0 | 1 | | 0 | 1 | |
| *Carex umbricola\|STARR_FORD\|STA_3\|\|spm00005220* | Non-Himalayan | 0 | 1 | | 0 | 1 | |
| *Carex umbrosa\|LUCENO\|NA\|BUL\|spm00000537* | Non-Himalayan | 0 | 1 | | 0 | 1 | |
| *Carex uncinata\|STARR_FORD\|\|NZN\|spm00005112* | Non-Himalayan | 0 | 1 | | 0 | 1 | |
| *Carex uncinioides\|JIMENEZMEJIAS\|\|CHT\|spm00003409* | Both | 1 | 1 | | 1 | 1 | |
| *Carex unilateralis\|STARR\|B985\|WAS\|spm00001592* | Non-Himalayan | 0 | 1 | | 0 | 1 | |
| *Carex ursina\|STARR\|B1368\|YUK\|spm00001975* | Non-Himalayan | 0 | 1 | | 0 | 1 | |
| *Carex ussuriensis\|LUCENO\|NA\|CHS\|spm00000533* | Non-Himalayan | 0 | 1 | | 0 | 1 | |
| *Carex utriculata\|STARR\|B774\|ARK\|spm00001379* | Non-Himalayan | 0 | 1 | | 0 | 1 | |
| *Carex vacillans\|STARR\|B1282\|MAI\|spm00001889* | Non-Himalayan | 0 | 1 | | 0 | 1 | |
| *Carex vaginosa\|JIMENEZMEJIAS\|\|CHT\|spm00003410* | Both | 1 | 1 | | 1 | 0 | |
| *Carex valbrayi\|HIPP\|NA\|MDG\|spm00002204* | Non-Himalayan | 0 | 1 | | 0 | 1 | |
| *Carex vallicola\|STARR\|B1379\|BRC\|spm00001986* | Non-Himalayan | 0 | 1 | | 0 | 1 | |
| *Carex vallispulchrae\|LUCENO\|NA\|AGS\|spm00000530* | Non-Himalayan | 0 | 1 | | 0 | 1 | |
| *Carex vallisrosetto\|GEHRKE\|NA\|UGA\|spm00000244* | Non-Himalayan | 0 | 1 | | 0 | 1 | |
| *Carex venusta\|STARR\|B767\|MSI\|spm00001372* | Non-Himalayan | 0 | 1 | | 0 | 1 | |
| *Carex vernacula\|STARR_FORD\|STA_882\|CAL\|spm00005382* | Non-Himalayan | 0 | 1 | | 0 | 1 | |
| *Carex verrucosa\|STARR\|B768\|FLA\|spm00001373* | Non-Himalayan | 0 | 1 | | 0 | 1 | |
| *Carex vesicaria\|STARR\|B755\|DEL\|spm00001360* | Non-Himalayan | 0 | 1 | | 0 | 1 | |
| *Carex vestita\|STARR\|B758\|DEL\|spm00001363* | Non-Himalayan | 0 | 1 | | 0 | 1 | |
| *Carex vexans\|STARR\|B761\|FLA\|spm00001366* | Non-Himalayan | 0 | 1 | | 0 | 1 | |
| *Carex vidua\|JIMENEZMEJIAS\|\|CHC\|spm00003411* | Both | 1 | 1 | | 1 | 1 | |
| *Carex virescens\|STARR\|B751\|ALA\|spm00001356* | Non-Himalayan | 0 | 1 | | 0 | 1 | |
| *Carex viridistellata\|STARR\|B1411\|\|spm00002018* | Non-Himalayan | 0 | 1 | | 0 | 1 | |
| *Carex viridula\|JIMENEZMEJIAS\|NA\|KGZ\|spm00003481* | Non-Himalayan | 0 | 1 | | 0 | 1 | |
| *Carex vixdentata\|JIMENEZMEJIAS\|\|AGE\|spm00004366* | Non-Himalayan | 0 | 1 | | 0 | 1 | |
| *Carex vulpinaris\|JIMENEZMEJIAS\|NA\|AFG\|spm00003473* | Both | 1 | 1 | | 1 | 0 | |
| *Carex vulpinoidea\|STARR\|B746\|CAL\|spm00001351* | Non-Himalayan | 0 | 1 | | 0 | 1 | |
| *Carex wahlenbergiana\|HIPP\|NA\|REU\|spm00002201* | Non-Himalayan | 0 | 1 | | 0 | 1 | |
| *Carex wallichiana\|PAK\|183066* | Both | 1 | 1 | | 1 | 0 | |
| *Carex whitneyi\|STARR\|B747\|CAL\|spm00001352* | Non-Himalayan | 0 | 1 | | 0 | 1 | |
| *Carex wiegandii\|STARR\|B748\|MAI\|spm00001353* | Non-Himalayan | 0 | 1 | | 0 | 1 | |
| *Carex willdenowii\|STARR\|B003\|KTY\|spm00000624* | Non-Himalayan | 0 | 1 | | 0 | 1 | |
| *Carex williamsii\|STARR\|B1362\|YUK\|spm00001969* | Non-Himalayan | 0 | 1 | | 0 | 1 | |
| *Carex woodii\|STARR\|B1183\|IOW\|spm00001790* | Non-Himalayan | 0 | 1 | | 0 | 1 | |
| *Carex wootonii\|STARR\|B1206\|ARI\|spm00001813* | Non-Himalayan | 0 | 1 | | 0 | 1 | |
| *Carex xalapensis\|REZNICEK\|NA\|MXC\|spm00000304* | Non-Himalayan | 0 | 1 | | 0 | 1 | |
| *Carex xerantica\|STARR\|B1306\|ONT\|spm00001913* | Non-Himalayan | 0 | 1 | | 0 | 1 | |
| *Carex yadongensis\|CHT\|E00177570* | Non-Himalayan | 0 | 1 | | 1 | 0 | |
| *Carex yandangshanica\|XIAOFENG\|NA\|CHS\|spm00000138* | Non-Himalayan | 0 | 1 | | 0 | 1 | |
| *Carex zikae\|STARR\|B966\|WAS\|spm00001573* | Non-Himalayan | 0 | 1 | | 0 | 1 | |
| *Carex zotovii\|JIMENEZMEJIAS\|\|\|spm00004411* | Non-Himalayan | 0 | 1 | | 0 | 1 | |
| *Carex zuluensis\|LUCENO\|NA\|NAT\|spm00000580* | Non-Himalayan | 0 | 1 | | 0 | 1 | |

*H=Himalayan

*N=Non-Himalayan
