# Supplementary material for: Timing and ecological priority shaped the diversification of sedges in the Himalayas
Source: PeerJ. 2019 Jun 7;7:e6792. doi: 10.7717/peerj.6792 (PMC6557248; doi:10.7717/peerj.6792)
Supplement: Table S3 — a Both constraint and unconstrained datasets contain same no. of taxa, in which 4 outgroup species also included b Total no. of character sites based on concatenated dataset alignment [file peerj-07-6792-s009.docx]

**Table S3** Dataset information for the five phylogenies used in constrained and unconstrained trees

| **Clades** | Vignea | Unispicate | Core Carex | Combined dataset (Vignea, Unispicate, core Carex) | Kobresia |
| --- | --- | --- | --- | --- | --- |
| **^a^Total no. of taxa** | 240 | 134 | 572 | 938 | 42 |
| **No. of Himalayan taxa** | 16 | 30 | 59 | 105 | 28 |
| **No. of non-Himalayan taxa** | 220 | 100 | 509 | 829 | 10 |
| **^b^Total character sites** | 2019 | 1849 | 2058 | 2080 | 1858 |
| **Model of evolution** | GTR+G | GTR+G | GTR+G | GTR+G | GTR+G |

^a^Both constraint and unconstrained datasets contain same no. of taxa, in which 4 outgroup species also included

^b^Total no. of character sites based on concatenated dataset alignment
