# Supplementary material for: Timing and ecological priority shaped the diversification of sedges in the Himalayas
Source: PeerJ. 2019 Jun 7;7:e6792. doi: 10.7717/peerj.6792 (PMC6557248; doi:10.7717/peerj.6792)
Supplement: Table S4 — a Here, W, Western Palearctic, E, Eastern Palearctic, N, Nearctic, A, Afrotropic, T, Neotropic, U, Australasia, I, Indo-Malaya, H, Himalaya, C, Antarctic and O, Oceania [file peerj-07-6792-s010.docx]

**Table S4 List of species with taxonomical sections and binary coding for their presence and absence in biogeographical regions according to broad sense assumption.** Here, W = Western Palearctic, E = Eastern Palearctic, N = Nearctic, A = Afrotropic, T = Neotropic, U = Australasia, I = Indo-Malaya, H = Himalaya, C = Antarctic and O = Oceania

| **Species** | **Sections** | **W** | **E** | **N** | **A** | **T** | **U** | **I** | **H** | **C** | **O** |
| --- | --- | --- | --- | --- | --- | --- | --- | --- | --- | --- | --- |
| *Carex aboriginum* | *Racemosae* | 0 | 0 | 1 | 0 | 0 | 0 | 0 | 0 | 0 | 0 |
| *Carex abrupta* | *Ovales* | 0 | 0 | 1 | 0 | 0 | 0 | 0 | 0 | 0 | 0 |
| *Carex abscondita* | *Careyanae* | 0 | 0 | 1 | 0 | 0 | 0 | 0 | 0 | 0 | 0 |
| *Carex acaulis* | *Abditispicae* | 0 | 0 | 0 | 0 | 1 | 0 | 0 | 0 | 1 | 0 |
| *Carex accrescens* | *Ammoglochin* | 0 | 1 | 0 | 0 | 0 | 0 | 0 | 0 | 0 | 0 |
| *Carex acicularis* | *Aciculares* | 0 | 0 | 0 | 0 | 0 | 1 | 0 | 0 | 0 | 0 |
| *Carex acidicola* | *Griseae* | 0 | 0 | 1 | 0 | 0 | 0 | 0 | 0 | 0 | 0 |
| *Carex acuta* | *Phacocystis* | 1 | 1 | 0 | 0 | 0 | 0 | 0 | 0 | 0 | 0 |
| *Carex acutiformis* | *Paludosae* | 1 | 1 | 0 | 1 | 0 | 0 | 0 | 1 | 0 | 0 |
| *Carex adelostoma* | *Racemosae* | 1 | 1 | 1 | 0 | 0 | 0 | 0 | 0 | 0 | 0 |
| *Carex adusta* | *Ovales* | 0 | 0 | 1 | 0 | 0 | 0 | 0 | 0 | 0 | 0 |
| *Carex aematorrhyncha* | *Paludosae* | 0 | 0 | 0 | 0 | 1 | 0 | 0 | 0 | 0 | 0 |
| *Carex aequialta* | *Phacocystis* | 0 | 1 | 0 | 0 | 0 | 0 | 0 | 0 | 0 | 0 |
| *Carex aestivalis* | *Hymenochlaenae* | 0 | 0 | 1 | 0 | 0 | 0 | 0 | 0 | 0 | 0 |
| *Carex aethiopica* | *Spirostachyae* | 0 | 0 | 0 | 1 | 0 | 0 | 0 | 0 | 0 | 0 |
| *Carex aggregata* | *Phaestoglochin* | 0 | 0 | 1 | 0 | 0 | 0 | 0 | 0 | 0 | 0 |
| *Carex agrostoides* | *Multiflorae* | 0 | 0 | 1 | 0 | 0 | 0 | 0 | 0 | 0 | 0 |
| *Carex alajica* | *Careyanae* | 0 | 1 | 0 | 0 | 0 | 0 | 0 | 0 | 0 | 0 |
| *Carex alata* | *Ovales* | 0 | 0 | 1 | 0 | 0 | 0 | 0 | 0 | 0 | 0 |
| *Carex alatauensis* | *Kobresia* | 0 | 1 | 0 | 0 | 0 | 0 | 0 | 1 | 0 | 0 |
| *Carex alba* | *Albae* | 1 | 1 | 0 | 0 | 0 | 0 | 0 | 0 | 0 | 0 |
| *Carex albata* | *Phleoideae* | 0 | 1 | 0 | 0 | 0 | 0 | 0 | 0 | 0 | 0 |
| *Carex albicans* | *Acrocystis* | 0 | 0 | 1 | 0 | 0 | 0 | 0 | 0 | 0 | 0 |
| *Carex albida* | *Aulocystis* | 0 | 0 | 1 | 0 | 0 | 0 | 0 | 0 | 0 | 0 |
| *Carex albolutescens* | *Ovales* | 0 | 0 | 1 | 0 | 1 | 0 | 0 | 0 | 0 | 0 |
| *Carex albonigra* | *Racemosae* | 0 | 0 | 1 | 0 | 0 | 0 | 0 | 0 | 0 | 0 |
| *Carex albursina* | *Laxiflorae* | 0 | 0 | 1 | 0 | 0 | 0 | 0 | 0 | 0 | 0 |
| *Carex allanii* | *Aciculares* | 0 | 0 | 0 | 0 | 0 | 1 | 0 | 0 | 0 | 0 |
| *Carex alma* | *Multiflorae* | 0 | 0 | 1 | 0 | 0 | 0 | 0 | 0 | 0 | 0 |
| *Carex alopecuroides* | *Molliculae* | 0 | 1 | 0 | 0 | 0 | 1 | 1 | 1 | 0 | 0 |
| *Carex alta* | *Planatae* | 0 | 1 | 0 | 0 | 0 | 0 | 1 | 1 | 0 | 0 |
| *Carex amphibola* | *Griseae* | 0 | 0 | 1 | 0 | 0 | 0 | 0 | 0 | 0 | 0 |
| *Carex amplifolia* | *Anomalae* | 0 | 0 | 1 | 0 | 0 | 0 | 0 | 0 | 0 | 0 |
| *Carex andersonii* | *Phacocystis* | 0 | 0 | 0 | 0 | 1 | 0 | 0 | 0 | 0 | 0 |
| *Carex andina* | *Junciformes* | 0 | 0 | 0 | 0 | 1 | 0 | 0 | 0 | 0 | 0 |
| *Carex andringitrensis* | *Indicae* | 0 | 0 | 0 | 1 | 0 | 0 | 0 | 0 | 0 | 0 |
| *Carex angolensis* | *Indicae* | 0 | 0 | 0 | 1 | 0 | 0 | 0 | 0 | 0 | 0 |
| *Carex angustata* | *Phacocystis* | 0 | 0 | 1 | 0 | 0 | 0 | 0 | 0 | 0 | 0 |
| *Carex angustilepis* | *Schiedeanae* | 0 | 0 | 1 | 0 | 0 | 0 | 0 | 0 | 0 | 0 |
| *Carex angustispica* | *Porocystis* | 0 | 0 | 1 | 0 | 0 | 0 | 0 | 0 | 0 | 0 |
| *Carex anisostachys* | *Longicaules* | 0 | 0 | 1 | 0 | 1 | 0 | 0 | 0 | 0 | 0 |
| *Carex annectens* | *Multiflorae* | 0 | 0 | 1 | 0 | 0 | 0 | 0 | 0 | 0 | 0 |
| *Carex anthoxanthea* | *Circinatae* | 0 | 1 | 1 | 0 | 0 | 0 | 0 | 0 | 0 | 0 |
| *Carex antoniensis* | *Vesicariae* | 1 | 0 | 0 | 0 | 0 | 0 | 0 | 0 | 0 | 0 |
| *Carex aperta* | *Phacocystis* | 0 | 1 | 1 | 0 | 0 | 0 | 0 | 0 | 0 | 0 |
| *Carex aphylla* | *Junciformes* | 0 | 0 | 0 | 0 | 1 | 0 | 0 | 0 | 0 | 0 |
| *Carex appalachica* | *Phaestoglochin* | 0 | 0 | 1 | 0 | 0 | 0 | 0 | 0 | 0 | 0 |
| *Carex appendiculata* | *Phacocystis* | 0 | 1 | 0 | 0 | 0 | 1 | 0 | 0 | 0 | 0 |
| *Carex appressa* | *Heleoglochin* | 0 | 0 | 0 | 0 | 0 | 1 | 0 | 0 | 0 | 1 |
| *Carex appropinquata* | *Heleoglochin* | 1 | 1 | 0 | 0 | 0 | 0 | 0 | 0 | 0 | 0 |
| *Carex aquatilis* | *Phacocystis* | 1 | 1 | 1 | 0 | 0 | 0 | 0 | 0 | 0 | 0 |
| *Carex arapahoensis* | *Ovales* | 0 | 0 | 1 | 0 | 0 | 0 | 0 | 0 | 0 | 0 |
| *Carex arcta* | *Glareosae* | 0 | 0 | 1 | 0 | 0 | 0 | 0 | 0 | 0 | 0 |
| *Carex arctata* | *Hymenochlaenae* | 0 | 0 | 1 | 0 | 0 | 0 | 0 | 0 | 0 | 0 |
| *Carex arctiformis* | *Glareosae* | 0 | 0 | 1 | 0 | 0 | 0 | 0 | 0 | 0 | 0 |
| *Carex arenaria* | *Ammoglochin* | 1 | 0 | 0 | 0 | 0 | 0 | 0 | 0 | 0 | 0 |
| *Carex argyrantha* | *Ovales* | 0 | 0 | 1 | 0 | 0 | 0 | 0 | 0 | 0 | 0 |
| *Carex arkansana* | *Phaestoglochin* | 0 | 0 | 1 | 0 | 0 | 0 | 0 | 0 | 0 | 0 |
| *Carex arnellii* | *Hymenochlaenae* | 1 | 1 | 0 | 0 | 0 | 0 | 0 | 0 | 0 | 0 |
| *Carex assiniboinensis* | *Hymenochlaenae* | 0 | 0 | 1 | 0 | 0 | 0 | 0 | 0 | 0 | 0 |
| *Carex astricta* | *Uncinia* | 0 | 0 | 0 | 0 | 0 | 1 | 0 | 0 | 0 | 0 |
| *Carex asturica* | *Paniceae* | 1 | 0 | 0 | 0 | 0 | 0 | 0 | 0 | 0 | 0 |
| *Carex asynchrona* | *Griseae* | 0 | 0 | 1 | 0 | 0 | 0 | 0 | 0 | 0 | 0 |
| *Carex atherodes* | *Carex* | 1 | 1 | 1 | 0 | 0 | 0 | 0 | 0 | 0 | 0 |
| *Carex athrostachya* | *Ovales* | 0 | 0 | 1 | 0 | 0 | 0 | 0 | 0 | 0 | 0 |
| *Carex atlantica* | *Stellulatae* | 0 | 0 | 1 | 0 | 1 | 0 | 0 | 0 | 0 | 0 |
| *Carex atractodes* | *Granulares* | 0 | 0 | 1 | 0 | 0 | 0 | 0 | 0 | 0 | 0 |
| *Carex atrata* | *Racemosae* | 1 | 1 | 1 | 0 | 0 | 0 | 0 | 1 | 0 | 0 |
| *Carex atratiformis* | *Racemosae* | 0 | 0 | 1 | 0 | 0 | 0 | 0 | 0 | 0 | 0 |
| *Carex atrofusca* | *Aulocystis* | 1 | 1 | 1 | 0 | 0 | 0 | 0 | 1 | 0 | 0 |
| *Carex atropicta* | *Racemosae* | 0 | 0 | 0 | 0 | 1 | 0 | 0 | 0 | 1 | 0 |
| *Carex atrosquama* | *Racemosae* | 0 | 0 | 1 | 0 | 0 | 0 | 0 | 0 | 0 | 0 |
| *Carex aucklandica* | *Uncinia* | 0 | 0 | 0 | 0 | 0 | 1 | 0 | 0 | 0 | 0 |
| *Carex augustinowiczii* | *Racemosae* | 0 | 1 | 0 | 0 | 0 | 0 | 0 | 0 | 0 | 0 |
| *Carex aurea* | *Bicolores* | 0 | 0 | 1 | 0 | 0 | 0 | 0 | 0 | 0 | 0 |
| *Carex aureolensis* | *Squarrosae* | 0 | 0 | 1 | 0 | 1 | 0 | 0 | 0 | 0 | 0 |
| *Carex auriculata* | *Paniceae* | 0 | 1 | 0 | 0 | 0 | 0 | 0 | 0 | 0 | 0 |
| *Carex austrina* | *Phaestoglochin* | 0 | 0 | 1 | 0 | 0 | 0 | 0 | 0 | 0 | 0 |
| *Carex austroalpina* | *Aulocystis* | 1 | 0 | 0 | 0 | 0 | 0 | 0 | 0 | 0 | 0 |
| *Carex austrocaroliniana* | *Careyanae* | 0 | 0 | 1 | 0 | 0 | 0 | 0 | 0 | 0 | 0 |
| *Carex austrocompacta* | *Uncinia* | 0 | 0 | 0 | 0 | 0 | 1 | 0 | 0 | 1 | 0 |
| *Carex austroflaccida* | *Uncinia* | 0 | 0 | 0 | 0 | 0 | 1 | 0 | 0 | 0 | 0 |
| *Carex austromexicana* | *Hymenochlaenae* | 0 | 0 | 1 | 0 | 0 | 0 | 0 | 0 | 0 | 0 |
| *Carex aztecica* | *Indicae* | 0 | 0 | 1 | 0 | 1 | 0 | 0 | 0 | 0 | 0 |
| *Carex azuayae* | *Phacocystis* | 0 | 0 | 0 | 0 | 1 | 0 | 0 | 0 | 0 | 0 |
| *Carex baccans* | *Polystachyae* | 0 | 1 | 0 | 0 | 0 | 1 | 1 | 1 | 0 | 0 |
| *Carex backii* | *Phyllostachyae* | 0 | 0 | 1 | 0 | 0 | 0 | 0 | 0 | 0 | 0 |
| *Carex baileyi* | *Vesicariae* | 0 | 0 | 1 | 0 | 0 | 0 | 0 | 0 | 0 | 0 |
| *Carex baimaensis* | *Careyanae* | 0 | 1 | 0 | 0 | 0 | 0 | 0 | 0 | 0 | 0 |
| *Carex baldensis* | *Baldenses* | 1 | 0 | 0 | 0 | 0 | 0 | 0 | 0 | 0 | 0 |
| *Carex balfourii* | *Ammoglochin* | 0 | 0 | 0 | 1 | 0 | 0 | 0 | 0 | 0 | 0 |
| *Carex baltzellii* | *Pictae* | 0 | 0 | 1 | 0 | 0 | 0 | 0 | 0 | 0 | 0 |
| *Carex banksiana* | *Uncinia* | 0 | 0 | 0 | 0 | 0 | 1 | 0 | 0 | 0 | 0 |
| *Carex banksii* | *Pellucidae* | 0 | 0 | 0 | 0 | 1 | 0 | 0 | 0 | 1 | 0 |
| *Carex barbarae* | *Phacocystis* | 0 | 0 | 1 | 0 | 0 | 0 | 0 | 0 | 0 | 0 |
| *Carex barrattii* | *Limosae* | 0 | 0 | 1 | 0 | 0 | 0 | 0 | 0 | 0 | 0 |
| *Carex basiantha* | *Phyllostachyae* | 0 | 0 | 1 | 0 | 0 | 0 | 0 | 0 | 0 | 0 |
| *Carex basutorum* | *Schoenoxiphium* | 0 | 0 | 0 | 1 | 0 | 0 | 0 | 0 | 0 | 0 |
| *Carex bathiei* | *Indicae* | 0 | 0 | 0 | 1 | 0 | 0 | 0 | 0 | 0 | 0 |
| *Carex bebbii* | *Ovales* | 0 | 0 | 1 | 0 | 0 | 0 | 0 | 0 | 0 | 0 |
| *Carex bella* | *Racemosae* | 0 | 0 | 1 | 0 | 0 | 0 | 0 | 0 | 0 | 0 |
| *Carex bequaertii* | *Rhynchocystis* | 0 | 0 | 0 | 1 | 0 | 0 | 0 | 0 | 0 | 0 |
| *Carex bhutanensis* | *Kobresia* | 0 | 0 | 0 | 0 | 0 | 0 | 0 | 1 | 0 | 0 |
| *Carex bicknellii* | *Ovales* | 0 | 0 | 1 | 0 | 0 | 0 | 0 | 0 | 0 | 0 |
| *Carex bicolor* | *Bicolores* | 1 | 1 | 1 | 0 | 0 | 0 | 0 | 0 | 0 | 0 |
| *Carex bigelowii* | *Phacocystis* | 1 | 1 | 1 | 0 | 0 | 0 | 0 | 1 | 0 | 0 |
| *Carex bijiangensis* | *Racemosae* | 0 | 0 | 0 | 0 | 0 | 0 | 0 | 1 | 0 | 0 |
| *Carex biltmoreana* | *Paniceae* | 0 | 0 | 1 | 0 | 0 | 0 | 0 | 0 | 0 | 0 |
| *Carex binervis* | *Spirostachyae* | 1 | 0 | 0 | 0 | 0 | 0 | 0 | 0 | 0 | 0 |
| *Carex bistaminata* | *Kobresia* | 0 | 1 | 0 | 0 | 0 | 0 | 0 | 1 | 0 | 0 |
| *Carex blakei* | *Spirostachyae* | 0 | 0 | 0 | 0 | 0 | 1 | 0 | 0 | 0 | 0 |
| *Carex blanda* | *Laxiflorae* | 0 | 0 | 1 | 0 | 0 | 0 | 0 | 0 | 0 | 0 |
| *Carex blepharicarpa* | *Aulocystis* | 0 | 1 | 0 | 0 | 0 | 0 | 0 | 0 | 0 | 0 |
| *Carex bohemica* | *Ovales* | 1 | 1 | 0 | 0 | 0 | 0 | 0 | 0 | 0 | 0 |
| *Carex bolanderi* | *Deweyanae* | 0 | 0 | 1 | 0 | 0 | 0 | 0 | 0 | 0 | 0 |
| *Carex boliviensis* | *Porocystis* | 0 | 0 | 1 | 0 | 1 | 0 | 0 | 0 | 0 | 0 |
| *Carex bonanzensis* | *Glareosae* | 0 | 1 | 1 | 0 | 0 | 0 | 0 | 0 | 0 | 0 |
| *Carex bonariensis* | *Bracteosae* | 0 | 0 | 0 | 0 | 1 | 0 | 0 | 0 | 0 | 0 |
| *Carex borbonica* | *Spirostachyae* | 0 | 0 | 0 | 1 | 0 | 0 | 0 | 0 | 0 | 0 |
| *Carex borealipolaris* | *Kobresia* | 1 | 1 | 1 | 0 | 0 | 0 | 0 | 0 | 0 | 0 |
| *Carex borii* | *Aulocystis* | 0 | 0 | 0 | 0 | 0 | 0 | 0 | 1 | 0 | 0 |
| *Carex boryana* | *Spirostachyae* | 0 | 0 | 0 | 1 | 0 | 0 | 0 | 0 | 0 | 0 |
| *Carex bostrychostigma* | *Debiles* | 0 | 1 | 0 | 0 | 0 | 0 | 0 | 0 | 0 | 0 |
| *Carex brachycalama* | *Paniceae* | 0 | 0 | 1 | 0 | 1 | 0 | 0 | 0 | 0 | 0 |
| *Carex brainerdii* | *Acrocystis* | 0 | 0 | 1 | 0 | 0 | 0 | 0 | 0 | 0 | 0 |
| *Carex brasiliensis* | *Glaucescentes* | 0 | 0 | 0 | 0 | 1 | 0 | 0 | 0 | 0 | 0 |
| *Carex breedlovei* | *Fecundae* | 0 | 0 | 1 | 0 | 0 | 0 | 0 | 0 | 0 | 0 |
| *Carex breviaristata* | *Mitratae* | 0 | 1 | 0 | 0 | 0 | 0 | 0 | 0 | 0 | 0 |
| *Carex brevicaulis* | *Uncinia* | 0 | 0 | 0 | 0 | 1 | 0 | 0 | 0 | 1 | 1 |
| *Carex brevicollis* | *Depauperatae* | 1 | 1 | 0 | 0 | 0 | 0 | 0 | 0 | 0 | 0 |
| *Carex brevior* | *Ovales* | 0 | 0 | 1 | 0 | 0 | 0 | 0 | 0 | 0 | 0 |
| *Carex breviprophylla* | *Kobresia* | 0 | 0 | 0 | 0 | 0 | 0 | 0 | 1 | 0 | 0 |
| *Carex breweri* | *Inflatae* | 0 | 0 | 1 | 0 | 0 | 0 | 0 | 0 | 0 | 0 |
| *Carex brizoides* | *Ammoglochin* | 1 | 1 | 0 | 0 | 0 | 0 | 0 | 0 | 0 | 0 |
| *Carex bromoides* | *Deweyanae* | 0 | 0 | 1 | 0 | 0 | 0 | 0 | 0 | 0 | 0 |
| *Carex brunnea* | *Graciles* | 0 | 1 | 0 | 1 | 0 | 1 | 1 | 1 | 0 | 0 |
| *Carex brunnescens* | *Glareosae* | 1 | 1 | 1 | 0 | 0 | 0 | 0 | 0 | 0 | 0 |
| *Carex brysonii* | *Griseae* | 0 | 0 | 1 | 0 | 0 | 0 | 0 | 0 | 0 | 0 |
| *Carex buekii* | *Phacocystis* | 1 | 1 | 0 | 0 | 0 | 0 | 0 | 0 | 0 | 0 |
| *Carex bulbostylis* | *Griseae* | 0 | 0 | 1 | 0 | 0 | 0 | 0 | 0 | 0 | 0 |
| *Carex bulgarica* | *Aulocystis* | 1 | 0 | 0 | 0 | 0 | 0 | 0 | 0 | 0 | 0 |
| *Carex bullata* | *Vesicariae* | 0 | 0 | 1 | 0 | 0 | 0 | 0 | 0 | 0 | 0 |
| *Carex burchelliana* | *Spirostachyae* | 0 | 0 | 0 | 1 | 0 | 0 | 0 | 0 | 0 | 0 |
| *Carex bushii* | *Porocystis* | 0 | 0 | 1 | 0 | 0 | 0 | 0 | 0 | 0 | 0 |
| *Carex buxbaumii* | *Racemosae* | 1 | 1 | 1 | 0 | 0 | 0 | 0 | 0 | 0 | 0 |
| *Carex calcifugens* | *Griseae* | 0 | 0 | 1 | 0 | 0 | 0 | 0 | 0 | 0 | 0 |
| *Carex calcis* | *Echinochlaenae* | 0 | 0 | 0 | 0 | 0 | 1 | 0 | 0 | 0 | 0 |
| *Carex californica* | *Paniceae* | 0 | 0 | 1 | 0 | 0 | 0 | 0 | 0 | 0 | 0 |
| *Carex caligena* | *unplaced_coreCarex* | 0 | 0 | 1 | 0 | 0 | 0 | 0 | 0 | 0 | 0 |
| *Carex camposii* | *Spirostachyae* | 1 | 0 | 0 | 0 | 0 | 0 | 0 | 0 | 0 | 0 |
| *Carex camptoglochin* | *Leucoglochin* | 0 | 0 | 0 | 0 | 1 | 0 | 0 | 0 | 1 | 0 |
| *Carex canariensis* | *Phaestoglochin* | 1 | 0 | 0 | 0 | 0 | 0 | 0 | 0 | 0 | 0 |
| *Carex candolleana* | *Mitratae* | 0 | 1 | 0 | 0 | 0 | 1 | 1 | 1 | 0 | 0 |
| *Carex canescens* | *Glareosae* | 1 | 1 | 1 | 0 | 1 | 1 | 0 | 1 | 1 | 0 |
| *Carex capensis* | *Schoenoxiphium* | 0 | 0 | 0 | 1 | 0 | 0 | 0 | 0 | 0 | 0 |
| *Carex capillaris* | *Chlorostachyae* | 1 | 1 | 1 | 0 | 0 | 0 | 0 | 1 | 0 | 0 |
| *Carex capillifolia* | *Kobresia* | 0 | 1 | 0 | 0 | 0 | 0 | 0 | 1 | 0 | 0 |
| *Carex capitata* | *Capituligerae* | 1 | 1 | 1 | 0 | 0 | 0 | 0 | 0 | 0 | 0 |
| *Carex capitellata* | *Rarae* | 0 | 1 | 0 | 0 | 0 | 0 | 0 | 0 | 0 | 0 |
| *Carex cardiolepis* | *Clandestinae* | 0 | 1 | 0 | 0 | 0 | 0 | 0 | 1 | 0 | 0 |
| *Carex careyana* | *Careyanae* | 0 | 0 | 1 | 0 | 0 | 0 | 0 | 0 | 0 | 0 |
| *Carex caroliniana* | *Porocystis* | 0 | 0 | 1 | 0 | 0 | 0 | 0 | 0 | 0 | 0 |
| *Carex caryophyllea* | *Mitratae* | 1 | 1 | 0 | 0 | 0 | 0 | 0 | 0 | 0 | 0 |
| *Carex castanea* | *Hymenochlaenae* | 0 | 0 | 1 | 0 | 0 | 0 | 0 | 0 | 0 | 0 |
| *Carex castroviejoi* | *Ceratocystis* | 1 | 0 | 0 | 0 | 0 | 0 | 0 | 0 | 0 | 0 |
| *Carex catharinensis* | *Spirostachyae* | 0 | 0 | 0 | 0 | 1 | 0 | 0 | 0 | 0 | 0 |
| *Carex caucasica* | *Racemosae* | 1 | 1 | 0 | 0 | 0 | 0 | 0 | 0 | 0 | 0 |
| *Carex caudata* | *Aulocystis* | 1 | 0 | 0 | 0 | 0 | 0 | 0 | 0 | 0 | 0 |
| *Carex cephaloidea* | *Phaestoglochin* | 0 | 0 | 1 | 0 | 0 | 0 | 0 | 0 | 0 | 0 |
| *Carex cephalophora* | *Phaestoglochin* | 0 | 0 | 1 | 0 | 0 | 0 | 0 | 0 | 0 | 0 |
| *Carex cercostachys* | *Kobresia* | 0 | 0 | 0 | 0 | 0 | 0 | 0 | 1 | 0 | 0 |
| *Carex chalciolepis* | *Racemosae* | 0 | 0 | 1 | 0 | 0 | 0 | 0 | 0 | 0 | 0 |
| *Carex chaofangii* | *Rhomboidales* | 0 | 1 | 0 | 0 | 0 | 0 | 0 | 0 | 0 | 0 |
| *Carex chapmanii* | *Laxiflorae* | 0 | 0 | 1 | 0 | 0 | 0 | 0 | 0 | 0 | 0 |
| *Carex cherokeensis* | *Hymenochlaenae* | 0 | 0 | 1 | 0 | 0 | 0 | 0 | 0 | 0 | 0 |
| *Carex chiapensis* | *Hymenochlaenae* | 0 | 0 | 1 | 0 | 0 | 0 | 0 | 0 | 0 | 0 |
| *Carex chihuahuensis* | *Multiflorae* | 0 | 0 | 1 | 0 | 0 | 0 | 0 | 0 | 0 | 0 |
| *Carex chlorosaccus* | *Indicae* | 0 | 0 | 0 | 1 | 0 | 0 | 0 | 0 | 0 | 0 |
| *Carex chordalis* | *Fecundae* | 0 | 0 | 1 | 0 | 1 | 0 | 0 | 0 | 0 | 0 |
| *Carex chordorrhiza* | *Chordorrhizae* | 1 | 1 | 1 | 0 | 0 | 0 | 0 | 0 | 0 | 0 |
| *Carex chungii* | *Mitratae* | 0 | 1 | 0 | 0 | 0 | 0 | 0 | 0 | 0 | 0 |
| *Carex circinata* | *Circinatae* | 0 | 1 | 1 | 0 | 0 | 0 | 0 | 0 | 0 | 0 |
| *Carex clavata* | *Spirostachyae* | 0 | 0 | 0 | 1 | 0 | 0 | 0 | 0 | 0 | 0 |
| *Carex cognata* | *Vesicariae* | 0 | 0 | 0 | 1 | 0 | 0 | 0 | 0 | 0 | 0 |
| *Carex colchica* | *Ammoglochin* | 1 | 1 | 0 | 0 | 0 | 0 | 0 | 0 | 0 | 0 |
| *Carex collinsii* | *Collinsiae* | 0 | 0 | 1 | 0 | 0 | 0 | 0 | 0 | 0 | 0 |
| *Carex collumanthus* | *Abditispicae* | 0 | 0 | 0 | 0 | 1 | 0 | 0 | 0 | 0 | 0 |
| *Carex comosa* | *Vesicariae* | 0 | 0 | 1 | 0 | 0 | 0 | 0 | 0 | 0 | 0 |
| *Carex complanata* | *Porocystis* | 0 | 0 | 1 | 0 | 1 | 0 | 0 | 0 | 0 | 0 |
| *Carex complexa* | *Schiedeanae* | 0 | 0 | 1 | 0 | 0 | 0 | 0 | 0 | 0 | 0 |
| *Carex composita* | *Polystachyae* | 0 | 1 | 0 | 0 | 0 | 0 | 1 | 1 | 0 | 0 |
| *Carex concinnoides* | *Clandestinae* | 0 | 0 | 1 | 0 | 0 | 0 | 0 | 0 | 0 | 0 |
| *Carex conferta* | *Stenorhynchae* | 0 | 0 | 0 | 1 | 0 | 0 | 0 | 0 | 0 | 0 |
| *Carex congdonii* | *Paludosae* | 0 | 0 | 1 | 0 | 0 | 0 | 0 | 0 | 0 | 0 |
| *Carex congolensis* | *Vesicariae* | 0 | 0 | 0 | 1 | 0 | 0 | 0 | 0 | 0 | 0 |
| *Carex coninux* | *Kobresia* | 0 | 1 | 0 | 0 | 0 | 0 | 0 | 1 | 0 | 0 |
| *Carex conjuncta* | *Vulpinae* | 0 | 0 | 1 | 0 | 0 | 0 | 0 | 0 | 0 | 0 |
| *Carex conoidea* | *Griseae* | 0 | 0 | 1 | 0 | 0 | 0 | 0 | 0 | 0 | 0 |
| *Carex cordillerana* | *Phyllostachyae* | 0 | 0 | 1 | 0 | 0 | 0 | 0 | 0 | 0 | 0 |
| *Carex corrugata* | *Griseae* | 0 | 0 | 1 | 0 | 0 | 0 | 0 | 0 | 0 | 0 |
| *Carex corynoidea* | *Uncinia* | 0 | 0 | 0 | 0 | 0 | 1 | 0 | 0 | 0 | 0 |
| *Carex coulteri* | *Longicaules* | 0 | 0 | 1 | 0 | 0 | 0 | 0 | 0 | 0 | 0 |
| *Carex crawei* | *Granulares* | 0 | 0 | 1 | 0 | 0 | 0 | 0 | 0 | 0 | 0 |
| *Carex crawfordii* | *Ovales* | 0 | 0 | 1 | 0 | 0 | 0 | 0 | 0 | 0 | 0 |
| *Carex crebra* | *Clandestinae* | 0 | 1 | 0 | 0 | 0 | 0 | 0 | 1 | 0 | 0 |
| *Carex crebriflora* | *Laxiflorae* | 0 | 0 | 1 | 0 | 0 | 0 | 0 | 0 | 0 | 0 |
| *Carex cretica* | *Sylvaticae* | 1 | 0 | 0 | 0 | 0 | 0 | 0 | 0 | 0 | 0 |
| *Carex crinalis* | *Longicaules* | 0 | 0 | 0 | 0 | 1 | 0 | 0 | 0 | 0 | 0 |
| *Carex crinita* | *Phacocystis* | 0 | 0 | 1 | 0 | 0 | 0 | 0 | 0 | 0 | 0 |
| *Carex crispa* | *Uncinia* | 0 | 0 | 0 | 0 | 0 | 1 | 0 | 0 | 0 | 0 |
| *Carex cristatella* | *Ovales* | 0 | 0 | 1 | 0 | 0 | 0 | 0 | 0 | 0 | 0 |
| *Carex cruenta* | *Aulocystis* | 0 | 0 | 0 | 0 | 0 | 0 | 0 | 1 | 0 | 0 |
| *Carex cruscorvi* | *Vulpinae* | 0 | 0 | 1 | 0 | 0 | 0 | 0 | 0 | 0 | 0 |
| *Carex cryptolepis* | *Ceratocystis* | 0 | 0 | 1 | 0 | 0 | 0 | 0 | 0 | 0 | 0 |
| *Carex cumberlandensis* | *Careyanae* | 0 | 0 | 1 | 0 | 0 | 0 | 0 | 0 | 0 | 0 |
| *Carex cumulata* | *Ovales* | 0 | 0 | 1 | 0 | 0 | 0 | 0 | 0 | 0 | 0 |
| *Carex curaica* | *Holarrhenae* | 0 | 1 | 0 | 0 | 0 | 0 | 0 | 1 | 0 | 0 |
| *Carex curatorum* | *Scirpinae* | 0 | 0 | 1 | 0 | 0 | 0 | 0 | 0 | 0 | 0 |
| *Carex curticeps* | *Kobresia* | 0 | 0 | 0 | 0 | 0 | 0 | 0 | 1 | 0 | 0 |
| *Carex curvula* | *Curvulae* | 1 | 0 | 0 | 0 | 0 | 0 | 0 | 0 | 0 | 0 |
| *Carex cusickii* | *Heleoglochin* | 0 | 0 | 1 | 0 | 0 | 0 | 0 | 0 | 0 | 0 |
| *Carex cyanea* | *Uncinia* | 0 | 0 | 0 | 0 | 0 | 1 | 0 | 0 | 0 | 0 |
| *Carex dacica* | *Phacocystis* | 1 | 0 | 0 | 0 | 0 | 0 | 0 | 0 | 0 | 0 |
| *Carex daltonii* | *Decorae* | 0 | 0 | 0 | 0 | 0 | 0 | 0 | 1 | 0 | 0 |
| *Carex dapanshanica* | *Mitratae* | 0 | 1 | 0 | 0 | 0 | 0 | 0 | 0 | 0 | 0 |
| *Carex darwinii* | *Phacocystis* | 0 | 0 | 0 | 0 | 1 | 0 | 0 | 0 | 0 | 0 |
| *Carex dasycarpa* | *Hallerianae* | 0 | 0 | 1 | 0 | 0 | 0 | 0 | 0 | 0 | 0 |
| *Carex davalliana* | *Physoglochin* | 1 | 1 | 0 | 0 | 0 | 0 | 0 | 0 | 0 | 0 |
| *Carex davidii* | *Mitratae* | 0 | 1 | 0 | 0 | 0 | 0 | 0 | 0 | 0 | 0 |
| *Carex davisii* | *Hymenochlaenae* | 0 | 0 | 1 | 0 | 0 | 0 | 0 | 0 | 0 | 0 |
| *Carex davyi* | *Ovales* | 0 | 0 | 1 | 0 | 0 | 0 | 0 | 0 | 0 | 0 |
| *Carex deasyi* | *Kobresia* | 0 | 1 | 0 | 0 | 0 | 0 | 0 | 1 | 0 | 0 |
| *Carex debilis* | *Hymenochlaenae* | 1 | 0 | 1 | 0 | 0 | 0 | 0 | 0 | 0 | 0 |
| *Carex decidua* | *Phacocystis* | 0 | 0 | 0 | 0 | 1 | 0 | 0 | 0 | 1 | 0 |
| *Carex decomposita* | *Heleoglochin* | 0 | 0 | 1 | 0 | 0 | 0 | 0 | 0 | 0 | 0 |
| *Carex decurtata* | *Echinochlaenae* | 0 | 0 | 0 | 0 | 0 | 1 | 0 | 0 | 0 | 0 |
| *Carex delicata* | *Chlorostachyae* | 0 | 1 | 0 | 0 | 0 | 0 | 0 | 0 | 0 | 0 |
| *Carex demissa* | *Ceratocystis* | 1 | 1 | 0 | 0 | 0 | 0 | 0 | 1 | 0 | 0 |
| *Carex densa* | *Multiflorae* | 0 | 0 | 1 | 0 | 0 | 0 | 0 | 0 | 0 | 0 |
| *Carex densipilosa* | *Lageniformes* | 0 | 1 | 0 | 0 | 0 | 0 | 0 | 0 | 0 | 0 |
| *Carex depauperata* | *Depauperatae* | 1 | 1 | 0 | 0 | 0 | 0 | 0 | 0 | 0 | 0 |
| *Carex derelicta* | *Ceratocystis* | 1 | 0 | 0 | 0 | 0 | 0 | 0 | 0 | 0 | 0 |
| *Carex deweyana* | *Deweyanae* | 0 | 0 | 1 | 0 | 0 | 0 | 0 | 0 | 0 | 0 |
| *Carex diandra* | *Heleoglochin* | 1 | 1 | 1 | 0 | 0 | 1 | 0 | 1 | 0 | 0 |
| *Carex diastena* | *Glareosae* | 0 | 1 | 0 | 0 | 0 | 0 | 0 | 0 | 0 | 0 |
| *Carex digitata* | *Clandestinae* | 1 | 1 | 0 | 0 | 0 | 0 | 0 | 0 | 0 | 0 |
| *Carex diluta* | *Spirostachyae* | 1 | 1 | 0 | 0 | 0 | 0 | 0 | 1 | 0 | 0 |
| *Carex dioica* | *Physoglochin* | 1 | 1 | 0 | 0 | 0 | 0 | 0 | 0 | 0 | 0 |
| *Carex disperma* | *Dispermae* | 1 | 1 | 1 | 0 | 0 | 0 | 0 | 0 | 0 | 0 |
| *Carex distachya* | *Schiedeanae* | 1 | 1 | 0 | 0 | 0 | 0 | 0 | 0 | 0 | 0 |
| *Carex distans* | *Spirostachyae* | 1 | 1 | 0 | 1 | 0 | 0 | 0 | 0 | 0 | 0 |
| *Carex disticha* | *Holarrhenae* | 1 | 1 | 0 | 0 | 0 | 0 | 0 | 0 | 0 | 0 |
| *Carex divisa* | *Divisae* | 1 | 1 | 0 | 1 | 0 | 0 | 0 | 1 | 0 | 0 |
| *Carex divulsa* | *Phaestoglochin* | 1 | 1 | 0 | 0 | 0 | 0 | 0 | 0 | 0 | 0 |
| *Carex doniana* | *Molliculae* | 0 | 1 | 0 | 0 | 0 | 0 | 1 | 1 | 0 | 0 |
| *Carex douglasii* | *Divisae* | 0 | 0 | 1 | 0 | 0 | 0 | 0 | 0 | 0 | 0 |
| *Carex drucei* | *Uncinia* | 0 | 0 | 0 | 0 | 0 | 1 | 0 | 0 | 0 | 0 |
| *Carex durangensis* | *Vesicariae* | 0 | 0 | 1 | 0 | 0 | 0 | 0 | 0 | 0 | 0 |
| *Carex durieui* | *Unplaced_coreCarex* | 1 | 0 | 0 | 0 | 0 | 0 | 0 | 0 | 0 | 0 |
| *Carex duriuscula* | *Divisae* | 0 | 1 | 1 | 0 | 0 | 1 | 0 | 0 | 0 | 0 |
| *Carex ebenea* | *Ovales* | 0 | 0 | 1 | 0 | 0 | 0 | 0 | 0 | 0 | 0 |
| *Carex eburnea* | *Albae* | 0 | 0 | 1 | 0 | 0 | 0 | 0 | 0 | 0 | 0 |
| *Carex echinata* | *Stellulatae* | 1 | 1 | 1 | 0 | 1 | 1 | 1 | 1 | 0 | 1 |
| *Carex echinochloe* | *Indicae* | 0 | 0 | 0 | 1 | 0 | 0 | 0 | 0 | 0 | 0 |
| *Carex echinodes* | *Ovales* | 0 | 0 | 1 | 0 | 0 | 0 | 0 | 0 | 0 | 0 |
| *Carex ecklonii* | *Spirostachyae* | 0 | 0 | 0 | 1 | 0 | 0 | 0 | 0 | 0 | 0 |
| *Carex ecuadorensis* | *Uncinia* | 0 | 0 | 0 | 0 | 1 | 0 | 0 | 0 | 0 | 0 |
| *Carex edura* | *Uncinia* | 0 | 0 | 0 | 0 | 0 | 1 | 0 | 0 | 1 | 0 |
| *Carex edwardsiana* | *Griseae* | 0 | 0 | 1 | 0 | 0 | 0 | 0 | 0 | 0 | 0 |
| *Carex egena* | *Paniceae* | 0 | 1 | 0 | 0 | 0 | 0 | 0 | 0 | 0 | 0 |
| *Carex egglestonii* | *Ovales* | 0 | 0 | 1 | 0 | 0 | 0 | 0 | 0 | 0 | 0 |
| *Carex egmontiana* | *Uncinia* | 0 | 0 | 0 | 0 | 0 | 1 | 0 | 0 | 0 | 0 |
| *Carex ehrenbergiana* | *Fecundae* | 0 | 0 | 1 | 0 | 0 | 0 | 0 | 0 | 0 | 0 |
| *Carex eleusinoides* | *Phacocystis* | 0 | 1 | 1 | 0 | 0 | 0 | 0 | 0 | 0 | 0 |
| *Carex elgonensis* | *Spirostachyae* | 0 | 0 | 0 | 1 | 0 | 0 | 0 | 0 | 0 | 0 |
| *Carex elliottii* | *Vesicariae* | 0 | 0 | 1 | 0 | 0 | 0 | 0 | 0 | 0 | 0 |
| *Carex elongata* | *Elongatae* | 1 | 1 | 0 | 0 | 0 | 0 | 0 | 0 | 0 | 0 |
| *Carex emoryi* | *Phacocystis* | 0 | 0 | 1 | 0 | 0 | 0 | 0 | 0 | 0 | 0 |
| *Carex endlichii* | *Phacocystis* | 0 | 0 | 1 | 0 | 1 | 0 | 0 | 0 | 0 | 0 |
| *Carex engelmannii* | *Inflatae* | 0 | 0 | 1 | 0 | 0 | 0 | 0 | 0 | 0 | 0 |
| *Carex enneastachya* | *Phacocystis* | 0 | 0 | 0 | 0 | 1 | 0 | 0 | 0 | 0 | 0 |
| *Carex erebus* | *Uncinia* | 0 | 0 | 0 | 0 | 0 | 1 | 0 | 0 | 1 | 0 |
| *Carex ericetorum* | *Acrocystis* | 1 | 1 | 0 | 0 | 0 | 0 | 0 | 0 | 0 | 0 |
| *Carex erinacea* | *Uncinia* | 0 | 0 | 0 | 0 | 1 | 0 | 0 | 0 | 0 | 0 |
| *Carex eriophylla* | *Carex* | 0 | 1 | 0 | 0 | 0 | 0 | 0 | 0 | 0 | 0 |
| *Carex erythrorrhiza* | *Stenorhynchae* | 0 | 0 | 0 | 1 | 0 | 0 | 0 | 0 | 0 | 0 |
| *Carex erythrovaginata* | *Uncinia* | 0 | 0 | 0 | 0 | 0 | 1 | 0 | 0 | 0 | 0 |
| *Carex esenbeckii* | *Kobresia* | 0 | 1 | 0 | 0 | 0 | 0 | 1 | 1 | 0 | 0 |
| *Carex euryphylla* | *Indicae* | 0 | 0 | 0 | 1 | 0 | 0 | 0 | 0 | 0 | 0 |
| *Carex exilis* | *Stellulatae* | 0 | 0 | 1 | 0 | 0 | 0 | 0 | 0 | 0 | 0 |
| *Carex exsiccata* | *Vesicariae* | 0 | 0 | 1 | 0 | 0 | 0 | 0 | 0 | 0 | 0 |
| *Carex extensa* | *Spirostachyae* | 1 | 1 | 0 | 0 | 0 | 0 | 0 | 0 | 0 | 0 |
| *Carex fecunda* | *Fecundae* | 0 | 0 | 0 | 0 | 1 | 0 | 0 | 0 | 0 | 0 |
| *Carex fedia* | *Carex* | 0 | 1 | 0 | 0 | 0 | 0 | 1 | 1 | 0 | 0 |
| *Carex festucacea* | *Ovales* | 0 | 0 | 1 | 0 | 0 | 0 | 0 | 0 | 0 | 0 |
| *Carex feta* | *Ovales* | 0 | 0 | 1 | 0 | 0 | 0 | 0 | 0 | 0 | 0 |
| *Carex filicina* | *Indicae* | 0 | 1 | 0 | 0 | 0 | 0 | 1 | 1 | 0 | 0 |
| *Carex filifolia* | *Filifoliae* | 0 | 0 | 1 | 0 | 0 | 0 | 0 | 0 | 0 | 0 |
| *Carex filipes* | *Paniceae* | 0 | 1 | 0 | 0 | 0 | 0 | 0 | 0 | 0 | 0 |
| *Carex filispica* | *Kobresia* | 0 | 0 | 0 | 0 | 0 | 0 | 0 | 1 | 0 | 0 |
| *Carex fimbriata* | *Aulocystis* | 1 | 0 | 0 | 0 | 0 | 0 | 0 | 0 | 0 | 0 |
| *Carex finitima* | *Debiles* | 0 | 1 | 0 | 0 | 0 | 1 | 1 | 1 | 0 | 0 |
| *Carex firma* | *Aulocystis* | 1 | 0 | 0 | 0 | 0 | 0 | 0 | 0 | 0 | 0 |
| *Carex firmula* | *Uncinia* | 0 | 0 | 1 | 0 | 1 | 0 | 0 | 0 | 1 | 0 |
| *Carex fischeri* | *Spirostachyae* | 0 | 0 | 0 | 1 | 0 | 0 | 0 | 0 | 0 | 0 |
| *Carex fissa* | *Multiflorae* | 0 | 0 | 1 | 0 | 0 | 0 | 0 | 0 | 0 | 0 |
| *Carex fissiglumis* | *Kobresia* | 0 | 0 | 0 | 0 | 0 | 0 | 0 | 1 | 0 | 0 |
| *Carex fissirostris* | *Spirostachyae* | 1 | 0 | 0 | 0 | 0 | 0 | 0 | 0 | 0 | 0 |
| *Carex fissuricola* | *Aulocystis* | 0 | 0 | 1 | 0 | 0 | 0 | 0 | 0 | 0 | 0 |
| *Carex flacca* | *Thuringiaca* | 1 | 1 | 0 | 0 | 0 | 0 | 0 | 1 | 0 | 0 |
| *Carex flaccosperma* | *Griseae* | 0 | 0 | 1 | 0 | 0 | 0 | 0 | 0 | 0 | 0 |
| *Carex flava* | *Ceratocystis* | 1 | 1 | 1 | 0 | 0 | 0 | 0 | 0 | 0 | 0 |
| *Carex floridana* | *Acrocystis* | 0 | 0 | 1 | 0 | 0 | 0 | 0 | 0 | 0 | 0 |
| *Carex fluviatilis* | *Phleoideae* | 0 | 1 | 0 | 0 | 0 | 0 | 1 | 1 | 0 | 0 |
| *Carex foenea* | *Ovales* | 0 | 0 | 1 | 0 | 0 | 0 | 0 | 0 | 0 | 0 |
| *Carex foetida* | *Foetidae* | 1 | 0 | 0 | 0 | 0 | 0 | 0 | 0 | 0 | 0 |
| *Carex folliculata* | *Rostrales* | 0 | 0 | 1 | 0 | 0 | 0 | 0 | 0 | 0 | 0 |
| *Carex foraminata* | *Mitratae* | 0 | 1 | 0 | 0 | 0 | 0 | 0 | 0 | 0 | 0 |
| *Carex formosa* | *Hymenochlaenae* | 0 | 0 | 1 | 0 | 0 | 0 | 0 | 0 | 0 | 0 |
| *Carex forsteri* | *Echinochlaenae* | 0 | 0 | 0 | 0 | 0 | 1 | 0 | 0 | 0 | 0 |
| *Carex fracta* | *Ovales* | 0 | 0 | 1 | 0 | 0 | 0 | 0 | 0 | 0 | 0 |
| *Carex frankii* | *Squarrosae* | 0 | 0 | 1 | 0 | 0 | 0 | 0 | 0 | 0 | 0 |
| *Carex fraseriana* | *Cymophyllus* | 0 | 0 | 1 | 0 | 0 | 0 | 0 | 0 | 0 | 0 |
| *Carex frigida* | *Aulocystis* | 1 | 0 | 0 | 0 | 0 | 0 | 0 | 0 | 0 | 0 |
| *Carex fritschii* | *Acrocystis* | 1 | 0 | 0 | 0 | 0 | 0 | 0 | 0 | 0 | 0 |
| *Carex fucata* | *Phacocystis* | 0 | 0 | 0 | 0 | 0 | 0 | 0 | 1 | 0 | 0 |
| *Carex fuliginosa* | *Aulocystis* | 1 | 1 | 1 | 0 | 0 | 0 | 0 | 0 | 0 | 0 |
| *Carex furva* | *Glareosae* | 1 | 0 | 0 | 0 | 0 | 0 | 0 | 0 | 0 | 0 |
| *Carex fuscula* | *Spirostachyae* | 0 | 0 | 0 | 0 | 1 | 0 | 0 | 0 | 1 | 0 |
| *Carex fusiformis* | *Debiles* | 0 | 0 | 0 | 0 | 0 | 0 | 0 | 1 | 0 | 0 |
| *Carex gammiei* | *Kobresia* | 0 | 0 | 0 | 0 | 0 | 0 | 0 | 1 | 0 | 0 |
| *Carex garberi* | *Bicolores* | 0 | 0 | 1 | 0 | 0 | 0 | 0 | 0 | 0 | 0 |
| *Carex gayana* | *Divisae* | 0 | 0 | 0 | 0 | 1 | 0 | 0 | 0 | 0 | 0 |
| *Carex genkaiensis* | *Mitratae* | 0 | 1 | 0 | 0 | 0 | 0 | 0 | 0 | 0 | 0 |
| *Carex gentilis* | *Graciles* | 0 | 1 | 0 | 0 | 0 | 0 | 0 | 1 | 0 | 0 |
| *Carex geophila* | *Acrocystis* | 0 | 0 | 1 | 0 | 1 | 0 | 0 | 0 | 0 | 0 |
| *Carex geyeri* | *Firmiculmes* | 0 | 0 | 1 | 0 | 0 | 0 | 0 | 0 | 0 | 0 |
| *Carex gholsonii* | *Granulares* | 0 | 0 | 1 | 0 | 0 | 0 | 0 | 0 | 0 | 0 |
| *Carex gibba* | *Gibbae* | 0 | 1 | 0 | 0 | 0 | 0 | 1 | 0 | 0 | 0 |
| *Carex gifuensis* | *Acrocystis* | 0 | 1 | 0 | 0 | 0 | 0 | 0 | 0 | 0 | 0 |
| *Carex gigantea* | *Lupulinae* | 0 | 0 | 1 | 0 | 0 | 0 | 0 | 0 | 0 | 0 |
| *Carex giraldiana* | *Rhomboidales* | 0 | 1 | 0 | 0 | 0 | 0 | 0 | 0 | 0 | 0 |
| *Carex glabrescens* | *Carex* | 0 | 1 | 0 | 0 | 0 | 0 | 0 | 0 | 0 | 0 |
| *Carex glacialis* | *Lamprochlaenae* | 1 | 1 | 1 | 0 | 0 | 0 | 0 | 0 | 0 | 0 |
| *Carex glareosa* | *Glareosae* | 1 | 1 | 1 | 0 | 0 | 0 | 0 | 0 | 0 | 0 |
| *Carex glaucescens* | *Glaucescentes* | 0 | 0 | 1 | 0 | 0 | 0 | 0 | 0 | 0 | 0 |
| *Carex glaucodea* | *Griseae* | 0 | 0 | 1 | 0 | 0 | 0 | 0 | 0 | 0 | 0 |
| *Carex globosa* | *Acrocystis* | 0 | 0 | 1 | 0 | 0 | 0 | 0 | 0 | 0 | 0 |
| *Carex globularis* | *Acrocystis* | 1 | 1 | 0 | 0 | 0 | 0 | 0 | 0 | 0 | 0 |
| *Carex glomerabilis* | *Stenorhynchae* | 0 | 0 | 0 | 1 | 0 | 0 | 0 | 0 | 0 | 0 |
| *Carex gmelinii* | *Racemosae* | 0 | 1 | 1 | 0 | 0 | 0 | 0 | 0 | 0 | 0 |
| *Carex godfreyi* | *Griseae* | 0 | 0 | 1 | 0 | 0 | 0 | 0 | 0 | 0 | 0 |
| *Carex gongshanensis* | *Graciles* | 0 | 0 | 0 | 0 | 0 | 0 | 0 | 1 | 0 | 0 |
| *Carex gotoi* | *Paludosae* | 0 | 1 | 0 | 0 | 0 | 0 | 0 | 0 | 0 | 0 |
| *Carex gracilior* | *Ovales* | 0 | 0 | 1 | 0 | 0 | 0 | 0 | 0 | 0 | 0 |
| *Carex gracillima* | *Hymenochlaenae* | 0 | 0 | 1 | 0 | 0 | 0 | 0 | 0 | 0 | 0 |
| *Carex graminifolia* | *Indicae* | 0 | 0 | 0 | 1 | 0 | 0 | 0 | 0 | 0 | 0 |
| *Carex grandiligulata* | *Siderostictae* | 0 | 1 | 0 | 0 | 0 | 0 | 0 | 0 | 0 | 0 |
| *Carex granularis* | *Granulares* | 0 | 0 | 1 | 0 | 0 | 0 | 0 | 0 | 0 | 0 |
| *Carex gravida* | *Phaestoglochin* | 0 | 0 | 1 | 0 | 0 | 0 | 0 | 0 | 0 | 0 |
| *Carex grayi* | *Lupulinae* | 0 | 0 | 1 | 0 | 0 | 0 | 0 | 0 | 0 | 0 |
| *Carex greenwayi* | *Spirostachyae* | 0 | 0 | 0 | 1 | 0 | 0 | 0 | 0 | 0 | 0 |
| *Carex grioletii* | *Acrocystis* | 1 | 1 | 0 | 0 | 0 | 0 | 0 | 0 | 0 | 0 |
| *Carex grisea* | *Griseae* | 0 | 0 | 1 | 0 | 0 | 0 | 0 | 0 | 0 | 0 |
| *Carex gunniana* | *Spirostachyae* | 0 | 0 | 0 | 0 | 0 | 1 | 0 | 0 | 0 | 0 |
| *Carex gynandra* | *Phacocystis* | 0 | 0 | 1 | 0 | 0 | 0 | 0 | 0 | 0 | 0 |
| *Carex gynocrates* | *Physoglochin* | 1 | 1 | 1 | 0 | 0 | 0 | 0 | 0 | 0 | 0 |
| *Carex gynodynama* | *Hymenochlaenae* | 0 | 0 | 1 | 0 | 0 | 0 | 0 | 0 | 0 | 0 |
| *Carex gypsophila* | *Schiedeanae* | 0 | 0 | 1 | 0 | 0 | 0 | 0 | 0 | 0 | 0 |
| *Carex haematostoma* | *Aulocystis* | 0 | 1 | 0 | 0 | 0 | 0 | 0 | 1 | 0 | 0 |
| *Carex halleriana* | *Hallerianae* | 1 | 1 | 0 | 0 | 0 | 0 | 0 | 1 | 0 | 0 |
| *Carex halliana* | *Paludosae* | 0 | 0 | 1 | 0 | 0 | 0 | 0 | 0 | 0 | 0 |
| *Carex hallii* | *Racemosae* | 0 | 0 | 1 | 0 | 0 | 0 | 0 | 0 | 0 | 0 |
| *Carex hamata* | *Uncinia* | 0 | 0 | 1 | 0 | 1 | 0 | 0 | 0 | 0 | 0 |
| *Carex hancockiana* | *Racemosae* | 0 | 1 | 0 | 0 | 0 | 0 | 0 | 0 | 0 | 0 |
| *Carex handelmazzettii* | *Kobresia* | 0 | 0 | 0 | 0 | 0 | 0 | 0 | 1 | 0 | 0 |
| *Carex harae* | *Kobresia* | 0 | 0 | 0 | 0 | 0 | 0 | 0 | 1 | 0 | 0 |
| *Carex harfordii* | *Ovales* | 0 | 0 | 1 | 0 | 0 | 0 | 0 | 0 | 0 | 0 |
| *Carex hartmanii* | *Racemosae* | 1 | 1 | 0 | 0 | 0 | 0 | 0 | 0 | 0 | 0 |
| *Carex hassei* | *Bicolores* | 0 | 0 | 1 | 0 | 0 | 0 | 0 | 0 | 0 | 0 |
| *Carex hastata* | *Rhomboidales* | 0 | 1 | 0 | 0 | 0 | 0 | 0 | 0 | 0 | 0 |
| *Carex haydeniana* | *Ovales* | 0 | 0 | 1 | 0 | 0 | 0 | 0 | 0 | 0 | 0 |
| *Carex haydenii* | *Phacocystis* | 0 | 0 | 1 | 0 | 0 | 0 | 0 | 0 | 0 | 0 |
| *Carex healyi* | *Uncinia* | 0 | 0 | 0 | 0 | 0 | 1 | 0 | 0 | 0 | 0 |
| *Carex hebes* | *Inversae* | 0 | 0 | 0 | 0 | 0 | 1 | 0 | 0 | 0 | 0 |
| *Carex heleonastes* | *Glareosae* | 1 | 1 | 1 | 0 | 0 | 0 | 0 | 0 | 0 | 0 |
| *Carex helleri* | *Racemosae* | 0 | 0 | 1 | 0 | 0 | 0 | 0 | 0 | 0 | 0 |
| *Carex helodes* | *Spirostachyae* | 1 | 0 | 0 | 0 | 0 | 0 | 0 | 0 | 0 | 0 |
| *Carex hendersonii* | *Laxiflorae* | 0 | 0 | 1 | 0 | 0 | 0 | 0 | 0 | 0 | 0 |
| *Carex heterodoxa* | *Indicae* | 0 | 0 | 0 | 1 | 0 | 0 | 0 | 0 | 0 | 0 |
| *Carex heteroneura* | *Racemosae* | 0 | 0 | 1 | 0 | 0 | 0 | 0 | 0 | 0 | 0 |
| *Carex heterostachya* | *Paludosae* | 0 | 1 | 0 | 0 | 0 | 0 | 0 | 0 | 0 | 0 |
| *Carex hirsutella* | *Porocystis* | 0 | 0 | 1 | 0 | 0 | 0 | 0 | 0 | 0 | 0 |
| *Carex hirta* | *Carex* | 1 | 1 | 0 | 0 | 0 | 0 | 0 | 0 | 0 | 0 |
| *Carex hirtifolia* | *Hirtifoliae* | 0 | 0 | 1 | 0 | 0 | 0 | 0 | 0 | 0 | 0 |
| *Carex hirtigluma* | *Indicae* | 0 | 0 | 0 | 1 | 0 | 0 | 0 | 0 | 0 | 0 |
| *Carex hirtissima* | *Hymenochlaenae* | 0 | 0 | 1 | 0 | 0 | 0 | 0 | 0 | 0 | 0 |
| *Carex hispida* | *Thuringiaca* | 1 | 1 | 0 | 0 | 0 | 0 | 0 | 0 | 0 | 0 |
| *Carex hitchcockiana* | *Griseae* | 0 | 0 | 1 | 0 | 0 | 0 | 0 | 0 | 0 | 0 |
| *Carex hochstetteriana* | *Spirostachyae* | 1 | 0 | 0 | 0 | 0 | 0 | 0 | 0 | 0 | 0 |
| *Carex holostoma* | *Racemosae* | 1 | 1 | 1 | 0 | 0 | 0 | 0 | 0 | 0 | 0 |
| *Carex hoodii* | *Phaestoglochin* | 0 | 0 | 1 | 0 | 0 | 0 | 0 | 0 | 0 | 0 |
| *Carex hookeriana* | *Phaestoglochin* | 0 | 0 | 1 | 0 | 0 | 0 | 0 | 0 | 0 | 0 |
| *Carex horizontalis* | *Uncinia* | 0 | 0 | 0 | 0 | 0 | 1 | 0 | 0 | 0 | 0 |
| *Carex hormathodes* | *Ovales* | 0 | 0 | 1 | 0 | 0 | 0 | 0 | 0 | 0 | 0 |
| *Carex hostiana* | *Ceratocystis* | 1 | 0 | 1 | 0 | 0 | 0 | 0 | 0 | 0 | 0 |
| *Carex houghtoniana* | *Paludosae* | 0 | 0 | 1 | 0 | 0 | 0 | 0 | 0 | 0 | 0 |
| *Carex hughii* | *Kobresia* | 0 | 1 | 0 | 0 | 0 | 0 | 0 | 1 | 0 | 0 |
| *Carex hultenii* | *Indicae* | 0 | 0 | 1 | 0 | 0 | 0 | 0 | 0 | 0 | 0 |
| *Carex humbertiana* | *Phacocystis* | 0 | 1 | 0 | 0 | 0 | 0 | 0 | 0 | 0 | 0 |
| *Carex humboldtiana* | *Indicae* | 0 | 0 | 1 | 0 | 1 | 0 | 0 | 0 | 0 | 0 |
| *Carex hyalina* | *Ovales* | 0 | 0 | 1 | 0 | 0 | 0 | 0 | 0 | 0 | 0 |
| *Carex hyalinolepis* | *Paludosae* | 0 | 0 | 1 | 0 | 0 | 0 | 0 | 0 | 0 | 0 |
| *Carex hypochlora* | *Mitratae* | 0 | 1 | 0 | 0 | 0 | 0 | 0 | 0 | 0 | 0 |
| *Carex hystericina* | *Vesicariae* | 0 | 0 | 1 | 0 | 1 | 0 | 0 | 0 | 0 | 0 |
| *Carex idaea* | *Spirostachyae* | 1 | 0 | 0 | 0 | 0 | 0 | 0 | 0 | 0 | 0 |
| *Carex idahoa* | *Racemosae* | 0 | 0 | 1 | 0 | 0 | 0 | 0 | 0 | 0 | 0 |
| *Carex iljinii* | *Ammoglochin* | 0 | 1 | 0 | 0 | 0 | 0 | 0 | 0 | 0 | 0 |
| *Carex illota* | *Glareosae* | 0 | 0 | 1 | 0 | 0 | 0 | 0 | 0 | 0 | 0 |
| *Carex imbecilla* | *Uncinia* | 0 | 0 | 0 | 0 | 0 | 1 | 0 | 0 | 0 | 0 |
| *Carex impressinervia* | *Griseae* | 0 | 0 | 1 | 0 | 0 | 0 | 0 | 0 | 0 | 0 |
| *Carex inanis* | *Setigerae* | 0 | 0 | 0 | 0 | 0 | 0 | 0 | 1 | 0 | 0 |
| *Carex incomitata* | *Heleoglochin* | 0 | 0 | 0 | 0 | 0 | 1 | 0 | 0 | 0 | 0 |
| *Carex incurviformis* | *Foetidae* | 1 | 1 | 1 | 0 | 0 | 0 | 0 | 0 | 0 | 0 |
| *Carex indica* | *Indicae* | 0 | 1 | 0 | 0 | 0 | 1 | 1 | 1 | 0 | 1 |
| *Carex infirminervia* | *Deweyanae* | 0 | 0 | 1 | 0 | 0 | 0 | 0 | 0 | 0 | 0 |
| *Carex infuscata* | *Racemosae* | 0 | 0 | 0 | 0 | 0 | 0 | 0 | 1 | 0 | 0 |
| *Carex integra* | *Ovales* | 0 | 0 | 1 | 0 | 0 | 0 | 0 | 0 | 0 | 0 |
| *Carex interior* | *Stellulatae* | 0 | 0 | 1 | 0 | 0 | 0 | 0 | 0 | 0 | 0 |
| *Carex interjecta* | *Ovales* | 1 | 0 | 0 | 0 | 0 | 0 | 0 | 0 | 0 | 0 |
| *Carex interrupta* | *Phacocystis* | 0 | 0 | 1 | 0 | 0 | 0 | 0 | 0 | 0 | 0 |
| *Carex intumescens* | *Lupulinae* | 0 | 0 | 1 | 0 | 0 | 0 | 0 | 0 | 0 | 0 |
| *Carex ischnostachya* | *Ischnostachyae* | 0 | 1 | 0 | 0 | 0 | 0 | 0 | 0 | 0 | 0 |
| *Carex ivanoviae* | *Lamprochlaenae* | 0 | 1 | 0 | 0 | 0 | 0 | 0 | 1 | 0 | 0 |
| *Carex jackiana* | *Paniceae* | 0 | 1 | 0 | 0 | 0 | 1 | 1 | 1 | 0 | 0 |
| *Carex jamesii* | *Phyllostachyae* | 0 | 0 | 1 | 0 | 0 | 0 | 0 | 0 | 0 | 0 |
| *Carex jamesonii* | *Fecundae* | 0 | 0 | 1 | 0 | 1 | 0 | 0 | 0 | 0 | 0 |
| *Carex jiuhuaensis* | *Rhomboidales* | 0 | 1 | 0 | 0 | 0 | 0 | 0 | 0 | 0 | 0 |
| *Carex johnstonii* | *Hymenochlaenae* | 0 | 0 | 0 | 1 | 0 | 0 | 0 | 0 | 0 | 0 |
| *Carex jonesii* | *Vulpinae* | 0 | 0 | 1 | 0 | 0 | 0 | 0 | 0 | 0 | 0 |
| *Carex joorii* | *Glaucescentes* | 0 | 0 | 1 | 0 | 0 | 0 | 0 | 0 | 0 | 0 |
| *Carex juniperorum* | *Phyllostachyae* | 0 | 0 | 1 | 0 | 0 | 0 | 0 | 0 | 0 | 0 |
| *Carex kamagariensis* | *Rhomboidales* | 0 | 1 | 0 | 0 | 0 | 0 | 1 | 0 | 0 | 0 |
| *Carex kanaii* | *Kobresia* | 0 | 0 | 0 | 0 | 0 | 0 | 0 | 1 | 0 | 0 |
| *Carex kansuensis* | *Racemosae* | 0 | 1 | 0 | 0 | 0 | 0 | 0 | 1 | 0 | 0 |
| *Carex killickii* | *Schoenoxiphium* | 0 | 0 | 0 | 1 | 0 | 0 | 0 | 0 | 0 | 0 |
| *Carex kingii* | *Uncinia* | 0 | 0 | 0 | 0 | 1 | 0 | 0 | 0 | 0 | 0 |
| *Carex kirinensis* | *Rarae* | 0 | 1 | 0 | 0 | 0 | 0 | 0 | 0 | 0 | 0 |
| *Carex kirkii* | *Ammoglochin* | 0 | 0 | 0 | 0 | 0 | 1 | 0 | 0 | 0 | 0 |
| *Carex kitaibeliana* | *Aulocystis* | 1 | 1 | 0 | 0 | 0 | 0 | 0 | 0 | 0 | 0 |
| *Carex klamathensis* | *Paniceae* | 0 | 0 | 1 | 0 | 0 | 0 | 0 | 0 | 0 | 0 |
| *Carex kobomugi* | *Macrocephalae* | 0 | 1 | 0 | 0 | 0 | 0 | 0 | 0 | 0 | 0 |
| *Carex kobresiformis* | *Rhomboidales* | 0 | 1 | 0 | 0 | 0 | 0 | 0 | 0 | 0 | 0 |
| *Carex kokanica* | *Kobresia* | 0 | 1 | 0 | 0 | 0 | 0 | 0 | 1 | 0 | 0 |
| *Carex koshewnikowii* | *Aulocystis* | 0 | 1 | 0 | 0 | 0 | 0 | 0 | 1 | 0 | 0 |
| *Carex kraliana* | *Laxiflorae* | 0 | 0 | 1 | 0 | 0 | 0 | 0 | 0 | 0 | 0 |
| *Carex krausei* | *Chlorostachyae* | 1 | 1 | 1 | 0 | 0 | 0 | 0 | 0 | 0 | 0 |
| *Carex kumaonensis* | *Decorae* | 0 | 0 | 0 | 0 | 0 | 0 | 0 | 1 | 0 | 0 |
| *Carex kurdica* | *Phacocystis* | 1 | 1 | 0 | 0 | 0 | 0 | 0 | 0 | 0 | 0 |
| *Carex lachenalii* | *Glareosae* | 1 | 1 | 1 | 0 | 0 | 1 | 0 | 0 | 0 | 0 |
| *Carex lacustris* | *Paludosae* | 0 | 0 | 1 | 0 | 0 | 0 | 0 | 0 | 0 | 0 |
| *Carex laeta* | *Clandestinae* | 0 | 0 | 0 | 0 | 0 | 0 | 0 | 1 | 0 | 0 |
| *Carex laeviconica* | *Carex* | 0 | 0 | 1 | 0 | 0 | 0 | 0 | 0 | 0 | 0 |
| *Carex laeviculmis* | *Glareosae* | 0 | 0 | 1 | 0 | 0 | 0 | 0 | 0 | 0 | 0 |
| *Carex laevigata* | *Spirostachyae* | 1 | 0 | 0 | 0 | 0 | 0 | 0 | 0 | 0 | 0 |
| *Carex laevissima* | *Phleoideae* | 0 | 1 | 0 | 0 | 0 | 0 | 0 | 0 | 0 | 0 |
| *Carex laevivaginata* | *Vulpinae* | 0 | 0 | 1 | 0 | 0 | 0 | 0 | 0 | 0 | 0 |
| *Carex lagunensis* | *Ovales* | 0 | 0 | 1 | 0 | 0 | 0 | 0 | 0 | 0 | 0 |
| *Carex lainzii* | *Spirostachyae* | 1 | 0 | 0 | 0 | 0 | 0 | 0 | 0 | 0 | 0 |
| *Carex lancea* | *Schoenoxiphium* | 0 | 0 | 0 | 1 | 0 | 0 | 0 | 0 | 0 | 0 |
| *Carex lancifolia* | *Clandestinae* | 0 | 1 | 0 | 0 | 0 | 0 | 0 | 0 | 0 | 0 |
| *Carex lapponica* | *Glareosae* | 1 | 1 | 1 | 0 | 0 | 0 | 0 | 0 | 0 | 0 |
| *Carex larensis* | *Ceratocystis* | 0 | 0 | 0 | 0 | 1 | 0 | 0 | 0 | 0 | 0 |
| *Carex lasiocarpa* | *Paludosae* | 1 | 1 | 1 | 0 | 0 | 0 | 0 | 0 | 0 | 0 |
| *Carex latebracteata* | *Phyllostachyae* | 0 | 0 | 1 | 0 | 0 | 0 | 0 | 0 | 0 | 0 |
| *Carex lativena* | *Hallerianae* | 0 | 0 | 1 | 0 | 0 | 0 | 0 | 0 | 0 | 0 |
| *Carex laxa* | *Paniceae* | 1 | 1 | 1 | 0 | 0 | 0 | 0 | 0 | 0 | 0 |
| *Carex laxiflora* | *Laxiflorae* | 0 | 0 | 1 | 0 | 0 | 0 | 0 | 0 | 0 | 0 |
| *Carex leavenworthii* | *Phaestoglochin* | 0 | 0 | 1 | 0 | 0 | 0 | 0 | 0 | 0 | 0 |
| *Carex ledebouriana* | *Chlorostachyae* | 1 | 1 | 0 | 0 | 0 | 0 | 0 | 0 | 0 | 0 |
| *Carex leersii* | *Phaestoglochin* | 1 | 1 | 0 | 0 | 0 | 0 | 0 | 1 | 0 | 0 |
| *Carex lehmannii* | *Racemosae* | 0 | 1 | 0 | 0 | 0 | 0 | 0 | 1 | 0 | 0 |
| *Carex leiorhyncha* | *Phleoideae* | 0 | 1 | 0 | 0 | 0 | 0 | 0 | 0 | 0 | 0 |
| *Carex lemmonii* | *Aulocystis* | 0 | 0 | 1 | 0 | 0 | 0 | 0 | 0 | 0 | 0 |
| *Carex leporina* | *Ovales* | 1 | 1 | 1 | 0 | 0 | 0 | 0 | 1 | 0 | 0 |
| *Carex leporinella* | *Ovales* | 0 | 0 | 1 | 0 | 0 | 0 | 0 | 0 | 0 | 0 |
| *Carex leptalea* | *Leptocephalae* | 0 | 0 | 1 | 0 | 1 | 0 | 0 | 0 | 0 | 0 |
| *Carex leptocladus* | *Indicae* | 0 | 0 | 0 | 1 | 0 | 0 | 0 | 0 | 0 | 0 |
| *Carex leptonervia* | *Laxiflorae* | 0 | 0 | 1 | 0 | 0 | 0 | 0 | 0 | 0 | 0 |
| *Carex leptopoda* | *Deweyanae* | 0 | 0 | 1 | 0 | 0 | 0 | 0 | 0 | 0 | 0 |
| *Carex leptosaccus* | *Stenorhynchae* | 0 | 0 | 0 | 1 | 0 | 0 | 0 | 0 | 0 | 0 |
| *Carex leucodonta* | *Acrocystis* | 0 | 0 | 1 | 0 | 0 | 0 | 0 | 0 | 0 | 0 |
| *Carex lianchengensis* | *Rhomboidales* | 0 | 1 | 0 | 0 | 0 | 0 | 0 | 0 | 0 | 0 |
| *Carex limosa* | *Limosae* | 1 | 1 | 1 | 0 | 1 | 0 | 0 | 0 | 0 | 0 |
| *Carex lingii* | *Hemiscaposae* | 0 | 1 | 0 | 0 | 0 | 0 | 0 | 0 | 0 | 0 |
| *Carex liparocarpos* | *Lamprochlaenae* | 1 | 1 | 0 | 0 | 0 | 0 | 0 | 0 | 0 | 0 |
| *Carex littledalei* | *Kobresia* | 0 | 0 | 0 | 0 | 0 | 0 | 0 | 1 | 0 | 0 |
| *Carex litvinovii* | *Aulocystis* | 0 | 1 | 0 | 0 | 0 | 0 | 0 | 0 | 0 | 0 |
| *Carex livida* | *Paniceae* | 1 | 1 | 1 | 0 | 1 | 0 | 0 | 0 | 0 | 0 |
| *Carex loliacea* | *Glareosae* | 1 | 1 | 1 | 0 | 0 | 0 | 0 | 0 | 0 | 0 |
| *Carex lonchocarpa* | *Rostrales* | 0 | 0 | 1 | 0 | 0 | 0 | 0 | 0 | 0 | 0 |
| *Carex longicaulis* | *Longicaules* | 0 | 0 | 1 | 0 | 0 | 0 | 0 | 0 | 0 | 0 |
| *Carex longicruris* | *Graciles* | 0 | 0 | 0 | 0 | 0 | 0 | 1 | 1 | 0 | 0 |
| *Carex longifructus* | *Uncinia* | 0 | 0 | 0 | 0 | 0 | 1 | 0 | 0 | 0 | 0 |
| *Carex longii* | *Ovales* | 0 | 0 | 1 | 0 | 1 | 0 | 0 | 0 | 0 | 0 |
| *Carex longipedunculata* | *Spirostachyae* | 0 | 0 | 0 | 1 | 0 | 0 | 0 | 0 | 0 | 0 |
| *Carex longipes* | *Graciles* | 0 | 1 | 0 | 0 | 0 | 1 | 1 | 1 | 0 | 0 |
| *Carex longirostrata* | *Phacocystis* | 0 | 1 | 0 | 0 | 0 | 0 | 0 | 0 | 0 | 0 |
| *Carex longissima* | *Filifoliae* | 0 | 0 | 1 | 0 | 0 | 0 | 0 | 0 | 0 | 0 |
| *Carex louisianica* | *Lupulinae* | 0 | 0 | 1 | 0 | 0 | 0 | 0 | 0 | 0 | 0 |
| *Carex lowei* | *Spirostachyae* | 1 | 0 | 0 | 0 | 0 | 0 | 0 | 0 | 0 | 0 |
| *Carex ludwigii* | *Schoenoxiphium* | 0 | 0 | 0 | 1 | 0 | 0 | 0 | 0 | 0 | 0 |
| *Carex lupuliformis* | *Lupulinae* | 0 | 0 | 1 | 0 | 0 | 0 | 0 | 0 | 0 | 0 |
| *Carex lupulina* | *Lupulinae* | 0 | 0 | 1 | 0 | 0 | 0 | 0 | 0 | 0 | 0 |
| *Carex lurida* | *Vesicariae* | 0 | 0 | 1 | 0 | 1 | 0 | 0 | 0 | 0 | 0 |
| *Carex lutea* | *Ceratocystis* | 0 | 0 | 1 | 0 | 0 | 0 | 0 | 0 | 0 | 0 |
| *Carex luzulifolia* | *Aulocystis* | 0 | 0 | 1 | 0 | 0 | 0 | 0 | 0 | 0 | 0 |
| *Carex lycurus* | *Stenorhynchae* | 0 | 0 | 0 | 1 | 0 | 0 | 0 | 0 | 0 | 0 |
| *Carex lyngbyei* | *Phacocystis* | 1 | 1 | 1 | 0 | 0 | 0 | 0 | 0 | 0 | 0 |
| *Carex maackii* | *Ovales* | 0 | 1 | 0 | 0 | 0 | 0 | 0 | 0 | 0 | 0 |
| *Carex mabilliana* | *Hallerianae* | 1 | 0 | 0 | 0 | 0 | 0 | 0 | 0 | 0 | 0 |
| *Carex mackenziei* | *Glareosae* | 1 | 1 | 1 | 0 | 0 | 0 | 0 | 0 | 0 | 0 |
| *Carex macloviana* | *Ovales* | 1 | 0 | 1 | 0 | 1 | 0 | 0 | 0 | 1 | 0 |
| *Carex macrocephala* | *Macrocephalae* | 0 | 1 | 1 | 0 | 0 | 0 | 0 | 0 | 0 | 0 |
| *Carex macrochaeta* | *Scitae* | 0 | 1 | 1 | 0 | 0 | 0 | 0 | 0 | 0 | 0 |
| *Carex macrolepis* | *Aulocystis* | 1 | 1 | 0 | 0 | 0 | 0 | 0 | 0 | 0 | 0 |
| *Carex macrophyllidion* | *Indicae* | 0 | 0 | 0 | 1 | 0 | 0 | 0 | 0 | 0 | 0 |
| *Carex macroprophylla* | *Kobresia* | 0 | 1 | 0 | 0 | 0 | 0 | 0 | 0 | 0 | 0 |
| *Carex macrosolen* | *Abditispicae* | 0 | 0 | 0 | 0 | 1 | 0 | 0 | 0 | 0 | 0 |
| *Carex macrostachys* | *Aulocystis* | 1 | 0 | 0 | 0 | 0 | 0 | 0 | 0 | 0 | 0 |
| *Carex macrostyla* | *unplaced* | 1 | 0 | 0 | 0 | 0 | 0 | 0 | 0 | 0 | 0 |
| *Carex madagascariensis* | *Phacocystis* | 0 | 0 | 0 | 1 | 0 | 0 | 0 | 0 | 0 | 0 |
| *Carex madida* | *Uncinia* | 0 | 0 | 0 | 0 | 1 | 0 | 0 | 0 | 0 | 0 |
| *Carex madrensis* | *Indicae* | 0 | 0 | 1 | 0 | 0 | 0 | 0 | 0 | 0 | 0 |
| *Carex magacis* | *Phaestoglochin* | 1 | 0 | 0 | 0 | 0 | 0 | 0 | 0 | 0 | 0 |
| *Carex magellanica* | *Limosae* | 1 | 1 | 1 | 0 | 1 | 0 | 0 | 0 | 1 | 0 |
| *Carex mairei* | *Spirostachyae* | 1 | 0 | 0 | 0 | 0 | 0 | 0 | 0 | 0 | 0 |
| *Carex manhartii* | *Laxiflorae* | 0 | 0 | 1 | 0 | 0 | 0 | 0 | 0 | 0 | 0 |
| *Carex mannii* | *Spirostachyae* | 0 | 0 | 0 | 1 | 0 | 0 | 0 | 0 | 0 | 0 |
| *Carex marianensis* | *Multiflorae* | 0 | 0 | 1 | 0 | 0 | 0 | 0 | 0 | 0 | 0 |
| *Carex marina* | *Glareosae* | 1 | 1 | 1 | 0 | 0 | 0 | 0 | 0 | 0 | 0 |
| *Carex mariposana* | *Ovales* | 0 | 0 | 1 | 0 | 0 | 0 | 0 | 0 | 0 | 0 |
| *Carex maritima* | *Foetidae* | 1 | 1 | 1 | 0 | 1 | 0 | 0 | 1 | 0 | 0 |
| *Carex meadii* | *Paniceae* | 0 | 0 | 1 | 0 | 0 | 0 | 0 | 0 | 0 | 0 |
| *Carex media* | *Racemosae* | 1 | 1 | 1 | 0 | 0 | 0 | 0 | 0 | 0 | 0 |
| *Carex megalepis* | *Uncinia* | 0 | 0 | 0 | 0 | 0 | 1 | 0 | 0 | 0 | 0 |
| *Carex melanantha* | *Racemosae* | 0 | 1 | 0 | 0 | 0 | 0 | 0 | 1 | 0 | 0 |
| *Carex melanocarpa* | *Acrocystis* | 1 | 1 | 0 | 0 | 0 | 0 | 0 | 0 | 0 | 0 |
| *Carex melanocephala* | *Racemosae* | 0 | 1 | 0 | 0 | 0 | 0 | 0 | 0 | 0 | 0 |
| *Carex melanostachya* | *Paludosae* | 1 | 1 | 0 | 0 | 0 | 0 | 0 | 0 | 0 | 0 |
| *Carex membranacea* | *Vesicariae* | 0 | 1 | 1 | 0 | 0 | 0 | 0 | 0 | 0 | 0 |
| *Carex mendocinensis* | *Hymenochlaenae* | 0 | 0 | 1 | 0 | 0 | 0 | 0 | 0 | 0 | 0 |
| *Carex meridensis* | *Uncinia* | 0 | 0 | 0 | 0 | 1 | 0 | 0 | 0 | 1 | 0 |
| *Carex meridiana* | *Mitratae* | 0 | 1 | 0 | 0 | 0 | 0 | 0 | 0 | 0 | 0 |
| *Carex merrittfernaldii* | *Ovales* | 0 | 0 | 1 | 0 | 0 | 0 | 0 | 0 | 0 | 0 |
| *Carex mertensii* | *Racemosae* | 0 | 1 | 1 | 0 | 0 | 0 | 0 | 0 | 0 | 0 |
| *Carex mesochorea* | *Phaestoglochin* | 0 | 0 | 1 | 0 | 0 | 0 | 0 | 0 | 0 | 0 |
| *Carex mesophila* | *Schiedeanae* | 0 | 0 | 1 | 0 | 0 | 0 | 0 | 0 | 0 | 0 |
| *Carex meyeriana* | *Stylosae* | 0 | 1 | 0 | 0 | 0 | 0 | 0 | 0 | 0 | 0 |
| *Carex michauxiana* | *Rostrales* | 0 | 1 | 1 | 0 | 0 | 1 | 0 | 0 | 0 | 0 |
| *Carex michelii* | *Depauperatae* | 1 | 1 | 0 | 0 | 0 | 0 | 0 | 0 | 0 | 0 |
| *Carex micrantha* | *Phacocystis* | 0 | 1 | 0 | 0 | 0 | 0 | 0 | 0 | 0 | 0 |
| *Carex microcarpa* | *Rhynchocystis* | 1 | 0 | 0 | 0 | 0 | 0 | 0 | 0 | 0 | 0 |
| *Carex microdonta* | *Granulares* | 0 | 0 | 1 | 0 | 0 | 0 | 0 | 0 | 0 | 0 |
| *Carex microglochin* | *Leucoglochin* | 1 | 1 | 1 | 0 | 1 | 0 | 0 | 1 | 0 | 0 |
| *Carex micropoda* | *Dornera* | 0 | 1 | 1 | 0 | 0 | 0 | 0 | 0 | 0 | 0 |
| *Carex microptera* | *Ovales* | 0 | 0 | 1 | 0 | 0 | 0 | 0 | 0 | 0 | 0 |
| *Carex mildbraediana* | *Spirostachyae* | 0 | 0 | 0 | 1 | 0 | 0 | 0 | 0 | 0 | 0 |
| *Carex minor* | *Uncinia* | 0 | 0 | 0 | 0 | 0 | 1 | 0 | 0 | 0 | 0 |
| *Carex misera* | *Hymenochlaenae* | 0 | 0 | 1 | 0 | 0 | 0 | 0 | 0 | 0 | 0 |
| *Carex missouriensis* | *Ovales* | 0 | 0 | 1 | 0 | 0 | 0 | 0 | 0 | 0 | 0 |
| *Carex mitchelliana* | *Phacocystis* | 0 | 0 | 1 | 0 | 0 | 0 | 0 | 0 | 0 | 0 |
| *Carex mitrata* | *Mitratae* | 0 | 1 | 0 | 0 | 0 | 0 | 1 | 0 | 0 | 0 |
| *Carex molesta* | *Ovales* | 0 | 0 | 1 | 0 | 0 | 0 | 0 | 0 | 0 | 0 |
| *Carex molestiformis* | *Ovales* | 0 | 0 | 1 | 0 | 0 | 0 | 0 | 0 | 0 | 0 |
| *Carex monostachya* | *Capituligerae* | 0 | 0 | 0 | 1 | 0 | 0 | 0 | 0 | 0 | 0 |
| *Carex monotropa* | *Ceratocystis* | 0 | 0 | 0 | 1 | 0 | 0 | 0 | 0 | 0 | 0 |
| *Carex montana* | *Acrocystis* | 1 | 1 | 0 | 0 | 0 | 0 | 0 | 0 | 0 | 0 |
| *Carex montis-everesti* | *Aulocystis* | 0 | 0 | 0 | 0 | 0 | 0 | 0 | 1 | 0 | 0 |
| *Carex moorcroftii* | *Racemosae* | 0 | 1 | 0 | 0 | 0 | 0 | 0 | 1 | 0 | 0 |
| *Carex morii* | *Decorae* | 0 | 1 | 0 | 0 | 0 | 0 | 0 | 0 | 0 | 0 |
| *Carex mossii* | *Rhynchocystis* | 0 | 0 | 0 | 1 | 0 | 0 | 0 | 0 | 0 | 0 |
| *Carex moupinensis* | *Hypolytroides* | 0 | 1 | 0 | 0 | 0 | 0 | 0 | 0 | 0 | 0 |
| *Carex mucronata* | *Aulocystis* | 1 | 0 | 0 | 0 | 0 | 0 | 0 | 0 | 0 | 0 |
| *Carex muehlenbergii* | *Phaestoglochin* | 0 | 0 | 1 | 0 | 1 | 0 | 0 | 0 | 0 | 0 |
| *Carex multicaulis* | *Firmiculmes* | 0 | 0 | 1 | 0 | 0 | 0 | 0 | 0 | 0 | 0 |
| *Carex multicostata* | *Ovales* | 0 | 0 | 1 | 0 | 0 | 0 | 0 | 0 | 0 | 0 |
| *Carex multifaria* | *Uncinia* | 0 | 0 | 0 | 0 | 1 | 0 | 0 | 0 | 0 | 0 |
| *Carex munda* | *Mundae* | 0 | 0 | 0 | 0 | 0 | 0 | 0 | 1 | 0 | 0 |
| *Carex muriculata* | *Schiedeanae* | 0 | 0 | 1 | 0 | 0 | 0 | 0 | 0 | 0 | 0 |
| *Carex muskingumensis* | *Ovales* | 0 | 0 | 1 | 0 | 0 | 0 | 0 | 0 | 0 | 0 |
| *Carex myosuroides* | *Kobresia* | 1 | 1 | 1 | 0 | 0 | 0 | 0 | 0 | 0 | 0 |
| *Carex nakaoana* | *Aulocystis* | 0 | 0 | 0 | 0 | 0 | 0 | 0 | 1 | 0 | 0 |
| *Carex nakasimae* | *Unplaced_coreCarex* | 0 | 1 | 0 | 0 | 0 | 0 | 0 | 0 | 0 | 0 |
| *Carex nardina* | *Nardinae* | 1 | 1 | 1 | 0 | 0 | 0 | 0 | 0 | 0 | 0 |
| *Carex nebraskensis* | *Phacocystis* | 0 | 0 | 1 | 0 | 0 | 0 | 0 | 0 | 0 | 0 |
| *Carex neesii* | *Kobresia* | 0 | 0 | 0 | 0 | 0 | 0 | 0 | 1 | 0 | 0 |
| *Carex negrii* | *Graciles* | 0 | 0 | 0 | 1 | 0 | 0 | 0 | 0 | 0 | 0 |
| *Carex nelsonii* | *Racemosae* | 0 | 0 | 1 | 0 | 0 | 0 | 0 | 0 | 0 | 0 |
| *Carex nemostachys* | *Confertiflorae* | 0 | 1 | 0 | 0 | 0 | 0 | 1 | 0 | 0 | 0 |
| *Carex nemurensis* | *Glareosae* | 0 | 1 | 0 | 0 | 0 | 0 | 0 | 0 | 0 | 0 |
| *Carex neochevalieri* | *Indicae* | 0 | 0 | 0 | 1 | 0 | 0 | 0 | 0 | 0 | 0 |
| *Carex nervata* | *Mitratae* | 0 | 1 | 0 | 0 | 0 | 0 | 0 | 0 | 0 | 0 |
| *Carex nervina* | *Vulpinae* | 0 | 0 | 1 | 0 | 0 | 0 | 0 | 0 | 0 | 0 |
| *Carex neurocarpa* | *Phleoideae* | 0 | 1 | 0 | 0 | 0 | 0 | 0 | 0 | 0 | 0 |
| *Carex neurophora* | *Vulpinae* | 0 | 0 | 1 | 0 | 0 | 0 | 0 | 0 | 0 | 0 |
| *Carex nigerrima* | *Racemosae* | 0 | 0 | 0 | 0 | 0 | 0 | 0 | 1 | 0 | 0 |
| *Carex nigra* | *Phacocystis* | 1 | 1 | 1 | 0 | 0 | 0 | 0 | 1 | 0 | 0 |
| *Carex nigricans* | *Dornera* | 0 | 0 | 1 | 0 | 0 | 0 | 0 | 0 | 0 | 0 |
| *Carex nigromarginata* | *Acrocystis* | 0 | 0 | 1 | 0 | 0 | 0 | 0 | 0 | 0 | 0 |
| *Carex nivalis* | *Aulocystis* | 0 | 1 | 0 | 0 | 0 | 0 | 0 | 1 | 0 | 0 |
| *Carex nodaeana* | *Rhomboidales* | 0 | 1 | 0 | 0 | 0 | 0 | 0 | 0 | 0 | 0 |
| *Carex normalis* | *Ovales* | 0 | 0 | 1 | 0 | 0 | 0 | 0 | 0 | 0 | 0 |
| *Carex norvegica* | *Racemosae* | 1 | 1 | 1 | 0 | 0 | 0 | 0 | 1 | 0 | 0 |
| *Carex nova* | *Racemosae* | 0 | 0 | 1 | 0 | 0 | 0 | 0 | 0 | 0 | 0 |
| *Carex novaeangliae* | *Acrocystis* | 0 | 0 | 1 | 0 | 0 | 0 | 0 | 0 | 0 | 0 |
| *Carex nubigena* | *Phleoideae* | 0 | 1 | 0 | 0 | 0 | 0 | 1 | 1 | 0 | 0 |
| *Carex nudata* | *Phacocystis* | 0 | 0 | 1 | 0 | 0 | 0 | 0 | 0 | 0 | 0 |
| *Carex obispoensis* | *Hymenochlaenae* | 0 | 0 | 1 | 0 | 0 | 0 | 0 | 0 | 0 | 0 |
| *Carex obliquicarpa* | *Confertiflorae* | 0 | 1 | 0 | 0 | 0 | 0 | 0 | 0 | 0 | 0 |
| *Carex obnupta* | *Phacocystis* | 0 | 0 | 1 | 0 | 0 | 0 | 0 | 0 | 0 | 0 |
| *Carex obovatosquamata* | *Clandestinae* | 0 | 0 | 0 | 0 | 0 | 0 | 0 | 1 | 0 | 0 |
| *Carex obscuriceps* | *Vesicariae* | 0 | 0 | 0 | 0 | 0 | 0 | 0 | 1 | 0 | 0 |
| *Carex obtusata* | *Obtusatae* | 1 | 1 | 1 | 0 | 0 | 0 | 0 | 0 | 0 | 0 |
| *Carex obtusifolia* | *Uncinia* | 0 | 0 | 0 | 0 | 0 | 1 | 0 | 0 | 0 | 0 |
| *Carex occidentalis* | *Phaestoglochin* | 0 | 0 | 1 | 0 | 0 | 0 | 0 | 0 | 0 | 0 |
| *Carex oedipostyla* | *Gynobasidae* | 1 | 0 | 0 | 0 | 0 | 0 | 0 | 0 | 0 | 0 |
| *Carex oklahomensis* | *Vulpinae* | 0 | 0 | 1 | 0 | 0 | 0 | 0 | 0 | 0 | 0 |
| *Carex olbiensis* | *Paniceae* | 1 | 0 | 0 | 0 | 0 | 0 | 0 | 0 | 0 | 0 |
| *Carex oligocarpa* | *Griseae* | 0 | 0 | 1 | 0 | 0 | 0 | 0 | 0 | 0 | 0 |
| *Carex oligocarya* | *Clandestinae* | 0 | 0 | 0 | 0 | 0 | 0 | 0 | 1 | 0 | 0 |
| *Carex oligosperma* | *Vesicariae* | 0 | 1 | 1 | 0 | 0 | 0 | 0 | 0 | 0 | 0 |
| *Carex opaca* | *Ovales* | 0 | 0 | 1 | 0 | 0 | 0 | 0 | 0 | 0 | 0 |
| *Carex oreophila* | *Capituligerae* | 0 | 1 | 0 | 0 | 0 | 0 | 0 | 0 | 0 | 0 |
| *Carex orizabae* | *Ovales* | 0 | 0 | 1 | 0 | 1 | 0 | 0 | 0 | 0 | 0 |
| *Carex ormostachya* | *Laxiflorae* | 0 | 0 | 1 | 0 | 0 | 0 | 0 | 0 | 0 | 0 |
| *Carex ornithopoda* | *Clandestinae* | 1 | 1 | 0 | 0 | 0 | 0 | 0 | 0 | 0 | 0 |
| *Carex oronensis* | *Ovales* | 0 | 0 | 1 | 0 | 0 | 0 | 0 | 0 | 0 | 0 |
| *Carex otrubae* | *Vulpinae* | 1 | 1 | 0 | 0 | 0 | 0 | 0 | 1 | 0 | 0 |
| *Carex ouachitana* | *Griseae* | 0 | 0 | 1 | 0 | 0 | 0 | 0 | 0 | 0 | 0 |
| *Carex ovatispiculata* | *Remotae* | 0 | 1 | 0 | 0 | 0 | 0 | 0 | 1 | 0 | 0 |
| *Carex ovoidispica* | *Kobresia* | 0 | 0 | 0 | 0 | 0 | 0 | 0 | 1 | 0 | 0 |
| *Carex oxylepis* | *Hymenochlaenae* | 0 | 0 | 1 | 0 | 0 | 0 | 0 | 0 | 0 | 0 |
| *Carex ozarkana* | *Ovales* | 0 | 0 | 1 | 0 | 0 | 0 | 0 | 0 | 0 | 0 |
| *Carex pachygyna* | *Siderostictae* | 0 | 1 | 0 | 0 | 0 | 0 | 0 | 0 | 0 | 0 |
| *Carex pachystachya* | *Ovales* | 0 | 1 | 1 | 0 | 0 | 0 | 0 | 0 | 0 | 0 |
| *Carex pachystylis* | *Physodeae* | 1 | 1 | 0 | 0 | 0 | 0 | 0 | 0 | 0 | 0 |
| *Carex paeninsulae* | *Griseae* | 0 | 0 | 1 | 0 | 0 | 0 | 0 | 0 | 0 | 0 |
| *Carex pairae* | *Phaestoglochin* | 1 | 1 | 0 | 0 | 0 | 0 | 0 | 0 | 0 | 0 |
| *Carex paleacea* | *Phacocystis* | 1 | 0 | 1 | 0 | 0 | 0 | 0 | 0 | 0 | 0 |
| *Carex pallescens* | *Porocystis* | 1 | 1 | 1 | 0 | 0 | 0 | 0 | 0 | 0 | 0 |
| *Carex pallidula* | *Clandestinae* | 1 | 0 | 0 | 0 | 0 | 0 | 0 | 0 | 0 | 0 |
| *Carex paneroi* | *Schiedeanae* | 0 | 0 | 1 | 0 | 0 | 0 | 0 | 0 | 0 | 0 |
| *Carex panicea* | *Paniceae* | 1 | 1 | 1 | 0 | 0 | 0 | 0 | 0 | 0 | 0 |
| *Carex paniculata* | *Heleoglochin* | 1 | 1 | 0 | 0 | 0 | 0 | 0 | 0 | 0 | 0 |
| *Carex panormitana* | *Phacocystis* | 1 | 0 | 0 | 0 | 0 | 0 | 0 | 0 | 0 | 0 |
| *Carex pansa* | *Divisae* | 0 | 0 | 1 | 0 | 0 | 0 | 0 | 0 | 0 | 0 |
| *Carex papulosa* | *Paniceae* | 0 | 1 | 0 | 0 | 0 | 0 | 0 | 0 | 0 | 0 |
| *Carex parallela* | *Physoglochin* | 1 | 1 | 1 | 0 | 0 | 0 | 0 | 0 | 0 | 0 |
| *Carex parryana* | *Racemosae* | 0 | 0 | 1 | 0 | 0 | 0 | 0 | 0 | 0 | 0 |
| *Carex parva* | *Leucoglochin* | 0 | 1 | 0 | 0 | 0 | 0 | 0 | 1 | 0 | 0 |
| *Carex parviflora* | *Racemosae* | 1 | 0 | 0 | 0 | 0 | 0 | 0 | 0 | 0 | 0 |
| *Carex parvispica* | *Uncinia* | 0 | 0 | 0 | 0 | 0 | 1 | 0 | 0 | 0 | 0 |
| *Carex parvula* | *Kobresia* | 0 | 1 | 0 | 0 | 0 | 0 | 1 | 1 | 0 | 0 |
| *Carex pauciflora* | *Leucoglochin* | 1 | 1 | 1 | 0 | 0 | 0 | 0 | 0 | 0 | 0 |
| *Carex paulovargasii* | *Spirostachyae* | 1 | 0 | 0 | 0 | 0 | 0 | 0 | 0 | 0 | 0 |
| *Carex paysonis* | *Scitae* | 0 | 0 | 1 | 0 | 0 | 0 | 0 | 0 | 0 | 0 |
| *Carex peckii* | *Acrocystis* | 0 | 0 | 1 | 0 | 0 | 0 | 0 | 0 | 0 | 0 |
| *Carex pediformis* | *Clandestinae* | 1 | 1 | 0 | 0 | 0 | 0 | 0 | 0 | 0 | 0 |
| *Carex pedunculata* | *Clandestinae* | 0 | 0 | 1 | 0 | 0 | 0 | 0 | 0 | 0 | 0 |
| *Carex peichuniana* | *Kobresia* | 0 | 0 | 0 | 0 | 0 | 0 | 0 | 1 | 0 | 0 |
| *Carex pellita* | *Paludosae* | 0 | 0 | 1 | 0 | 0 | 0 | 0 | 0 | 0 | 0 |
| *Carex pelocarpa* | *Racemosae* | 0 | 0 | 1 | 0 | 0 | 0 | 0 | 0 | 0 | 0 |
| *Carex pendula* | *Rhynchocystis* | 1 | 1 | 0 | 0 | 0 | 0 | 0 | 0 | 0 | 0 |
| *Carex pensylvanica* | *Acrocystis* | 0 | 0 | 1 | 0 | 0 | 0 | 0 | 0 | 0 | 0 |
| *Carex perdentata* | *Phaestoglochin* | 0 | 0 | 1 | 0 | 0 | 0 | 0 | 0 | 0 | 0 |
| *Carex peregrina* | *Psyllophora* | 1 | 0 | 0 | 1 | 0 | 0 | 0 | 0 | 0 | 0 |
| *Carex perglobosa* | *Foetidae* | 0 | 0 | 1 | 0 | 0 | 0 | 0 | 0 | 0 | 0 |
| *Carex perplexa* | *Uncinia* | 0 | 0 | 0 | 0 | 0 | 1 | 0 | 0 | 0 | 0 |
| *Carex perraudieriana* | *Spirostachyae* | 1 | 0 | 0 | 0 | 0 | 0 | 0 | 0 | 0 | 0 |
| *Carex perstricta* | *Schiedeanae* | 0 | 0 | 1 | 0 | 0 | 0 | 0 | 0 | 0 | 0 |
| *Carex petasata* | *Ovales* | 0 | 0 | 1 | 0 | 0 | 0 | 0 | 0 | 0 | 0 |
| *Carex petitiana* | *Spirostachyae* | 0 | 0 | 0 | 1 | 0 | 0 | 0 | 0 | 0 | 0 |
| *Carex peucophila* | *Ovales* | 0 | 0 | 1 | 0 | 1 | 0 | 0 | 0 | 0 | 0 |
| *Carex phaeocephala* | *Ovales* | 0 | 0 | 1 | 0 | 0 | 0 | 0 | 0 | 0 | 0 |
| *Carex phleoides* | *Uncinia* | 0 | 0 | 1 | 0 | 1 | 0 | 0 | 0 | 0 | 0 |
| *Carex phragmitoides* | *Vesicariae* | 0 | 0 | 0 | 1 | 0 | 0 | 0 | 0 | 0 | 0 |
| *Carex phyllostachys* | *Phyllostachys* | 1 | 1 | 0 | 0 | 0 | 0 | 0 | 0 | 0 | 0 |
| *Carex physodes* | *Physodeae* | 1 | 1 | 0 | 0 | 0 | 0 | 0 | 1 | 0 | 0 |
| *Carex pichinchensis* | *Fecundae* | 0 | 0 | 0 | 0 | 1 | 0 | 0 | 0 | 0 | 0 |
| *Carex picta* | *Pictae* | 0 | 0 | 1 | 0 | 0 | 0 | 0 | 0 | 0 | 0 |
| *Carex pigra* | *Griseae* | 0 | 0 | 1 | 0 | 0 | 0 | 0 | 0 | 0 | 0 |
| *Carex pilosa* | *Paniceae* | 1 | 0 | 0 | 0 | 0 | 0 | 0 | 0 | 0 | 0 |
| *Carex pilulifera* | *Acrocystis* | 1 | 0 | 0 | 0 | 0 | 0 | 0 | 0 | 0 | 0 |
| *Carex pinophila* | *Hymenochlaenae* | 0 | 0 | 1 | 0 | 0 | 0 | 0 | 0 | 0 | 0 |
| *Carex pisiformis* | *Mitratae* | 0 | 1 | 0 | 0 | 0 | 0 | 1 | 0 | 0 | 0 |
| *Carex pityophila* | *Acrocystis* | 0 | 0 | 1 | 0 | 0 | 0 | 0 | 0 | 0 | 0 |
| *Carex planata* | *Remotae* | 0 | 1 | 0 | 0 | 0 | 0 | 0 | 0 | 0 | 0 |
| *Carex planilomina* | *Schiedeanae* | 0 | 0 | 1 | 0 | 0 | 0 | 0 | 0 | 0 | 0 |
| *Carex planispicata* | *Griseae* | 0 | 0 | 1 | 0 | 0 | 0 | 0 | 0 | 0 | 0 |
| *Carex planostachys* | *Hallerianae* | 0 | 0 | 1 | 0 | 1 | 0 | 0 | 0 | 0 | 0 |
| *Carex plantaginea* | *Careyanae* | 0 | 0 | 1 | 0 | 0 | 0 | 0 | 0 | 0 | 0 |
| *Carex platyphylla* | *Careyanae* | 0 | 0 | 1 | 0 | 0 | 0 | 0 | 0 | 0 | 0 |
| *Carex plectobasis* | *Aulocystis* | 0 | 0 | 0 | 0 | 0 | 0 | 0 | 1 | 0 | 0 |
| *Carex pleiostachys* | *Echinochlaenae* | 0 | 0 | 0 | 0 | 0 | 1 | 0 | 0 | 0 | 0 |
| *Carex pluriflora* | *Limosae* | 0 | 1 | 1 | 0 | 0 | 0 | 0 | 0 | 0 | 0 |
| *Carex podocarpa* | *Scitae* | 0 | 1 | 1 | 0 | 0 | 0 | 0 | 0 | 0 | 0 |
| *Carex polymorpha* | *Paniceae* | 0 | 0 | 1 | 0 | 0 | 0 | 0 | 0 | 0 | 0 |
| *Carex polyschoenoides* | *Mitratae* | 0 | 1 | 0 | 0 | 0 | 0 | 0 | 0 | 0 | 0 |
| *Carex polystachya* | *Indicae* | 0 | 0 | 1 | 0 | 1 | 0 | 0 | 0 | 0 | 0 |
| *Carex polysticha* | *Vesicariae* | 0 | 0 | 1 | 0 | 1 | 0 | 0 | 0 | 0 | 0 |
| *Carex pomiensis* | *Decorae* | 0 | 0 | 0 | 0 | 0 | 0 | 0 | 1 | 0 | 0 |
| *Carex potens* | *Uncinia* | 0 | 0 | 0 | 0 | 0 | 1 | 0 | 0 | 0 | 0 |
| *Carex potosina* | *Potosinae* | 0 | 0 | 1 | 0 | 0 | 0 | 0 | 0 | 0 | 0 |
| *Carex praeceptorium* | *Glareosae* | 0 | 0 | 1 | 0 | 0 | 0 | 0 | 0 | 0 | 0 |
| *Carex praeclara* | *Racemosae* | 0 | 0 | 0 | 0 | 0 | 0 | 0 | 1 | 0 | 0 |
| *Carex praecox* | *Ammoglochin* | 1 | 1 | 0 | 0 | 0 | 0 | 0 | 0 | 0 | 0 |
| *Carex praegracilis* | *Divisae* | 0 | 0 | 1 | 0 | 1 | 0 | 0 | 0 | 0 | 0 |
| *Carex prainii* | *Kobresia* | 0 | 0 | 0 | 0 | 0 | 0 | 0 | 1 | 0 | 0 |
| *Carex prairea* | *Heleoglochin* | 0 | 0 | 1 | 0 | 0 | 0 | 0 | 0 | 0 | 0 |
| *Carex prasina* | *Hymenochlaenae* | 0 | 0 | 1 | 0 | 0 | 0 | 0 | 0 | 0 | 0 |
| *Carex praticola* | *Ovales* | 0 | 0 | 1 | 0 | 0 | 0 | 0 | 0 | 0 | 0 |
| *Carex preslii* | *Ovales* | 0 | 0 | 1 | 0 | 0 | 0 | 0 | 0 | 0 | 0 |
| *Carex pringlei* | *Thuringiaca* | 0 | 0 | 1 | 0 | 0 | 0 | 0 | 0 | 0 | 0 |
| *Carex projecta* | *Ovales* | 0 | 0 | 1 | 0 | 0 | 0 | 0 | 0 | 0 | 0 |
| *Carex proposita* | *Ovales* | 0 | 0 | 1 | 0 | 0 | 0 | 0 | 0 | 0 | 0 |
| *Carex proxima* | *Indicae* | 0 | 0 | 0 | 1 | 0 | 0 | 0 | 0 | 0 | 0 |
| *Carex pruinosa* | *Phacocystis* | 0 | 1 | 0 | 0 | 0 | 0 | 1 | 1 | 0 | 0 |
| *Carex pseudobicolor* | *Racemosae* | 0 | 0 | 0 | 0 | 0 | 0 | 0 | 1 | 0 | 0 |
| *Carex pseudobrizoides* | *Ammoglochin* | 1 | 0 | 0 | 0 | 0 | 0 | 0 | 0 | 0 | 0 |
| *Carex pseudochinensis* | *Anomalae* | 0 | 1 | 0 | 0 | 0 | 0 | 0 | 0 | 0 | 0 |
| *Carex pseudocuraica* | *Chordorrhizae* | 0 | 1 | 0 | 0 | 0 | 0 | 0 | 0 | 0 | 0 |
| *Carex pseudocyperus* | *Vesicariae* | 1 | 1 | 1 | 0 | 0 | 1 | 1 | 1 | 0 | 0 |
| *Carex pseudofoetida* | *Foetidae* | 0 | 1 | 0 | 0 | 0 | 0 | 0 | 1 | 0 | 0 |
| *Carex pseudogammiei* | *Kobresia* | 0 | 0 | 0 | 0 | 0 | 0 | 0 | 1 | 0 | 0 |
| *Carex pseudolaxa* | *Kobresia* | 0 | 0 | 0 | 0 | 0 | 0 | 0 | 1 | 0 | 0 |
| *Carex pseudololiacea* | *Glareosae* | 0 | 1 | 0 | 0 | 0 | 0 | 0 | 0 | 0 | 0 |
| *Carex pseudorufa* | *Schoenoxiphium* | 0 | 0 | 0 | 1 | 0 | 0 | 0 | 0 | 0 | 0 |
| *Carex pseudotristachya* | *Mitratae* | 0 | 1 | 0 | 0 | 0 | 0 | 0 | 0 | 0 | 0 |
| *Carex pseuduncinoides* | *Kobresia* | 0 | 1 | 0 | 0 | 0 | 0 | 0 | 1 | 0 | 0 |
| *Carex pterocarpa* | *Ammoglochin* | 0 | 0 | 0 | 0 | 0 | 1 | 0 | 0 | 0 | 0 |
| *Carex pulchra* | *Decorae* | 0 | 0 | 0 | 0 | 0 | 0 | 0 | 1 | 0 | 0 |
| *Carex pulicaris* | *Psyllophora* | 1 | 0 | 0 | 0 | 0 | 0 | 0 | 0 | 0 | 0 |
| *Carex pumila* | *Paludosae* | 0 | 1 | 0 | 0 | 0 | 1 | 0 | 0 | 0 | 0 |
| *Carex punctata* | *Spirostachyae* | 1 | 1 | 0 | 0 | 0 | 0 | 0 | 0 | 0 | 0 |
| *Carex punicea* | *Uncinia* | 0 | 0 | 0 | 0 | 0 | 1 | 0 | 0 | 0 | 0 |
| *Carex purpurata* | *Uncinia* | 0 | 0 | 0 | 0 | 0 | 1 | 0 | 0 | 0 | 0 |
| *Carex purpurifera* | *Laxiflorae* | 0 | 0 | 1 | 0 | 0 | 0 | 0 | 0 | 0 | 0 |
| *Carex pycnostachys* | *Foetidae* | 0 | 1 | 0 | 0 | 0 | 0 | 0 | 0 | 0 | 0 |
| *Carex pyramidalis* | *Indicae* | 0 | 0 | 0 | 1 | 0 | 0 | 0 | 0 | 0 | 0 |
| *Carex pyrenaica* | *Dornera* | 1 | 1 | 0 | 0 | 0 | 0 | 0 | 0 | 0 | 0 |
| *Carex qiyunensis* | *Mitratae* | 0 | 1 | 0 | 0 | 0 | 0 | 0 | 0 | 0 | 0 |
| *Carex quadriflora* | *Clandestinae* | 0 | 1 | 0 | 0 | 0 | 0 | 0 | 0 | 0 | 0 |
| *Carex queretarensis* | *Hymenochlaenae* | 0 | 0 | 1 | 0 | 0 | 0 | 0 | 0 | 0 | 0 |
| *Carex raddei* | *Carex* | 0 | 1 | 0 | 0 | 0 | 0 | 0 | 0 | 0 | 0 |
| *Carex radfordii* | *Laxiflorae* | 0 | 0 | 1 | 0 | 0 | 0 | 0 | 0 | 0 | 0 |
| *Carex radiata* | *Phaestoglochin* | 0 | 0 | 1 | 0 | 0 | 0 | 0 | 0 | 0 | 0 |
| *Carex radicalis* | *Radicales* | 0 | 0 | 0 | 0 | 0 | 0 | 0 | 1 | 0 | 0 |
| *Carex rainbowii* | *Sylvaticae* | 0 | 0 | 0 | 1 | 0 | 0 | 0 | 0 | 0 | 0 |
| *Carex ramenskii* | *Phacocystis* | 0 | 1 | 1 | 0 | 0 | 0 | 0 | 0 | 0 | 0 |
| *Carex ramosa* | *Indicae* | 0 | 0 | 0 | 1 | 0 | 0 | 0 | 0 | 0 | 0 |
| *Carex randalpina* | *Phacocystis* | 1 | 0 | 0 | 0 | 0 | 0 | 0 | 0 | 0 | 0 |
| *Carex rariflora* | *Limosae* | 1 | 1 | 1 | 0 | 0 | 0 | 0 | 0 | 0 | 0 |
| *Carex raynoldsii* | *Racemosae* | 0 | 0 | 1 | 0 | 0 | 0 | 0 | 0 | 0 | 0 |
| *Carex remota* | *Remotae* | 1 | 1 | 0 | 0 | 0 | 0 | 0 | 1 | 0 | 0 |
| *Carex remotiuscula* | *Remotae* | 0 | 1 | 0 | 0 | 0 | 0 | 0 | 1 | 0 | 0 |
| *Carex renauldii* | *Hymenochlaenae* | 0 | 0 | 0 | 1 | 0 | 0 | 0 | 0 | 0 | 0 |
| *Carex reniformis* | *Ovales* | 0 | 0 | 1 | 0 | 0 | 0 | 0 | 0 | 0 | 0 |
| *Carex renschiana* | *Indicae* | 0 | 0 | 0 | 1 | 0 | 0 | 0 | 0 | 0 | 0 |
| *Carex resectans* | *Inversae* | 0 | 0 | 0 | 0 | 0 | 1 | 0 | 0 | 0 | 0 |
| *Carex retroflexa* | *Phaestoglochin* | 0 | 0 | 1 | 0 | 0 | 0 | 0 | 0 | 0 | 0 |
| *Carex retrorsa* | *Vesicariae* | 0 | 0 | 1 | 0 | 0 | 0 | 0 | 0 | 0 | 0 |
| *Carex reznicekii* | *Acrocystis* | 0 | 0 | 1 | 0 | 0 | 0 | 0 | 0 | 0 | 0 |
| *Carex rhizina* | *Clandestinae* | 1 | 1 | 0 | 0 | 0 | 0 | 0 | 0 | 0 | 0 |
| *Carex rhodesiaca* | *Phacocystis* | 0 | 0 | 0 | 1 | 0 | 0 | 0 | 0 | 0 | 0 |
| *Carex rhynchoperigynium* | *Hymenochlaenae* | 0 | 0 | 1 | 0 | 0 | 0 | 0 | 0 | 0 | 0 |
| *Carex richardsonii* | *Clandestinae* | 0 | 0 | 1 | 0 | 0 | 0 | 0 | 0 | 0 | 0 |
| *Carex riloensis* | *Acrocystis* | 1 | 0 | 0 | 0 | 0 | 0 | 0 | 0 | 0 | 0 |
| *Carex rivulorum* | *Rhomboidales* | 0 | 1 | 0 | 0 | 0 | 0 | 0 | 0 | 0 | 0 |
| *Carex roanensis* | *Hymenochlaenae* | 0 | 0 | 1 | 0 | 0 | 0 | 0 | 0 | 0 | 0 |
| *Carex rochebrunii* | *Remotae* | 0 | 1 | 0 | 0 | 0 | 0 | 1 | 1 | 0 | 0 |
| *Carex rorulenta* | *Hallerianae* | 1 | 0 | 0 | 0 | 0 | 0 | 0 | 0 | 0 | 0 |
| *Carex rosea* | *Phaestoglochin* | 0 | 0 | 1 | 0 | 0 | 0 | 0 | 0 | 0 | 0 |
| *Carex rossii* | *Acrocystis* | 0 | 0 | 1 | 0 | 0 | 0 | 0 | 0 | 0 | 0 |
| *Carex rostrata* | *Vesicariae* | 1 | 1 | 1 | 0 | 0 | 0 | 0 | 1 | 0 | 0 |
| *Carex rotundata* | *Vesicariae* | 1 | 1 | 1 | 0 | 0 | 0 | 0 | 0 | 0 | 0 |
| *Carex rubicunda* | *Echinochlaenae* | 0 | 0 | 0 | 0 | 0 | 1 | 0 | 0 | 0 | 0 |
| *Carex rufulistolon* | *Thuringiaca* | 0 | 0 | 0 | 0 | 0 | 0 | 0 | 1 | 0 | 0 |
| *Carex rugulosa* | *Paludosae* | 0 | 1 | 0 | 0 | 0 | 0 | 0 | 0 | 0 | 0 |
| *Carex runssoroensis* | *Capituligerae* | 0 | 0 | 0 | 1 | 0 | 0 | 0 | 0 | 0 | 0 |
| *Carex rupestris* | *Petraeae* | 1 | 1 | 1 | 0 | 0 | 0 | 0 | 0 | 0 | 0 |
| *Carex rutenbergiana* | *Indicae* | 0 | 0 | 0 | 1 | 0 | 0 | 0 | 0 | 0 | 0 |
| *Carex ruthii* | *Stellulatae* | 0 | 0 | 1 | 0 | 0 | 0 | 0 | 0 | 0 | 0 |
| *Carex sabulosa* | *Racemosae* | 0 | 1 | 1 | 0 | 0 | 0 | 0 | 0 | 0 | 0 |
| *Carex sagei* | *Ceratocystis* | 0 | 0 | 0 | 0 | 1 | 0 | 0 | 0 | 1 | 0 |
| *Carex salina* | *Phacocystis* | 1 | 1 | 1 | 0 | 0 | 0 | 0 | 0 | 0 | 0 |
| *Carex salticola* | *Uncinia* | 0 | 0 | 0 | 0 | 1 | 0 | 0 | 0 | 0 | 0 |
| *Carex sanguinea* | *Kobresia* | 0 | 0 | 0 | 0 | 0 | 0 | 0 | 1 | 0 | 0 |
| *Carex sargentiana* | *Kobresia* | 0 | 1 | 0 | 0 | 0 | 0 | 0 | 1 | 0 | 0 |
| *Carex sartwelliana* | *Paludosae* | 0 | 0 | 1 | 0 | 0 | 0 | 0 | 0 | 0 | 0 |
| *Carex sartwellii* | *Holarrhenae* | 0 | 0 | 1 | 0 | 0 | 0 | 0 | 0 | 0 | 0 |
| *Carex saxatilis* | *Vesicariae* | 1 | 1 | 1 | 0 | 0 | 0 | 0 | 0 | 0 | 0 |
| *Carex saximontana* | *Phyllostachyae* | 0 | 0 | 1 | 0 | 0 | 0 | 0 | 0 | 0 | 0 |
| *Carex scabrata* | *Anomalae* | 0 | 0 | 1 | 0 | 0 | 0 | 0 | 0 | 0 | 0 |
| *Carex scabriuscula* | *Scirpinae* | 0 | 0 | 1 | 0 | 0 | 0 | 0 | 0 | 0 | 0 |
| *Carex scaposa* | *Hemiscaposae* | 0 | 1 | 0 | 0 | 0 | 0 | 1 | 0 | 0 | 0 |
| *Carex schiedeana* | *Schiedeanae* | 0 | 0 | 1 | 0 | 0 | 0 | 0 | 0 | 0 | 0 |
| *Carex schimperiana* | *Schoenoxiphium* | 0 | 0 | 0 | 1 | 0 | 0 | 0 | 0 | 0 | 0 |
| *Carex schlagintweitiana* | *Setigerae* | 0 | 0 | 0 | 0 | 0 | 0 | 0 | 1 | 0 | 0 |
| *Carex schottii* | *Phacocystis* | 0 | 0 | 1 | 0 | 0 | 0 | 0 | 0 | 0 | 0 |
| *Carex schweickerdtii* | *Schoenoxiphium* | 0 | 0 | 0 | 1 | 0 | 0 | 0 | 0 | 0 | 0 |
| *Carex schweinitzii* | *Vesicariae* | 0 | 0 | 1 | 0 | 0 | 0 | 0 | 0 | 0 | 0 |
| *Carex scirpoidea* | *Scirpinae* | 1 | 1 | 1 | 0 | 0 | 0 | 0 | 0 | 0 | 0 |
| *Carex senanensis* | *Deweyanae* | 0 | 1 | 0 | 0 | 0 | 0 | 0 | 0 | 0 | 0 |
| *Carex senta* | *Phacocystis* | 0 | 0 | 1 | 0 | 0 | 0 | 0 | 0 | 0 | 0 |
| *Carex seorsa* | *Stellulatae* | 0 | 0 | 1 | 0 | 0 | 0 | 0 | 0 | 0 | 0 |
| *Carex serpenticola* | *Acrocystis* | 0 | 0 | 1 | 0 | 0 | 0 | 0 | 0 | 0 | 0 |
| *Carex serratodens* | *Racemosae* | 0 | 0 | 1 | 0 | 0 | 0 | 0 | 0 | 0 | 0 |
| *Carex setschwanensis* | *Kobresia* | 0 | 1 | 0 | 0 | 0 | 0 | 0 | 1 | 0 | 0 |
| *Carex shangchengensis* | *Rhomboidales* | 0 | 1 | 0 | 0 | 0 | 0 | 0 | 0 | 0 | 0 |
| *Carex sheldonii* | *Carex* | 0 | 0 | 1 | 0 | 0 | 0 | 0 | 0 | 0 | 0 |
| *Carex shinnersii* | *Ovales* | 0 | 0 | 1 | 0 | 0 | 0 | 0 | 0 | 0 | 0 |
| *Carex shortiana* | *Shortianae* | 0 | 0 | 1 | 0 | 0 | 0 | 0 | 0 | 0 | 0 |
| *Carex siccata* | *Ammoglochin* | 0 | 0 | 1 | 0 | 0 | 0 | 0 | 0 | 0 | 0 |
| *Carex siderosticta* | *Siderostictae* | 0 | 1 | 0 | 0 | 0 | 0 | 0 | 0 | 0 | 0 |
| *Carex silicea* | *Ovales* | 0 | 0 | 1 | 0 | 0 | 0 | 0 | 0 | 0 | 0 |
| *Carex silvestris* | *Uncinia* | 0 | 0 | 0 | 0 | 0 | 1 | 0 | 0 | 0 | 0 |
| *Carex simensis* | *Spirostachyae* | 0 | 0 | 0 | 1 | 0 | 0 | 0 | 0 | 0 | 0 |
| *Carex simpliciuscula* | *Kobresia* | 1 | 1 | 1 | 0 | 0 | 0 | 0 | 1 | 0 | 0 |
| *Carex simulans* | *Rhomboidales* | 0 | 1 | 0 | 0 | 0 | 0 | 0 | 0 | 0 | 0 |
| *Carex simulata* | *Divisae* | 0 | 0 | 1 | 0 | 0 | 0 | 0 | 0 | 0 | 0 |
| *Carex socialis* | *Phaestoglochin* | 0 | 0 | 1 | 0 | 0 | 0 | 0 | 0 | 0 | 0 |
| *Carex songorica* | *Paludosae* | 0 | 1 | 0 | 0 | 0 | 0 | 0 | 1 | 0 | 0 |
| *Carex sorianoi* | *Junciformes* | 0 | 0 | 0 | 0 | 1 | 0 | 0 | 0 | 0 | 0 |
| *Carex sororia* | *Bracteosae* | 0 | 0 | 0 | 0 | 1 | 0 | 0 | 0 | 0 | 0 |
| *Carex sparganioides* | *Phaestoglochin* | 0 | 0 | 1 | 0 | 0 | 0 | 0 | 0 | 0 | 0 |
| *Carex spartea* | *Schoenoxiphium* | 0 | 0 | 0 | 1 | 0 | 0 | 0 | 0 | 0 | 0 |
| *Carex specifica* | *Ovales* | 0 | 0 | 1 | 0 | 0 | 0 | 0 | 0 | 0 | 0 |
| *Carex spectabilis* | *Scitae* | 0 | 1 | 1 | 0 | 0 | 0 | 0 | 0 | 0 | 0 |
| *Carex specuicola* | *Racemosae* | 0 | 0 | 1 | 0 | 0 | 0 | 0 | 0 | 0 | 0 |
| *Carex sphaerogyna* | *Vesicariae* | 0 | 0 | 0 | 1 | 0 | 0 | 0 | 0 | 0 | 0 |
| *Carex spicata* | *Phaestoglochin* | 1 | 1 | 0 | 0 | 0 | 0 | 0 | 0 | 0 | 0 |
| *Carex spissa* | *Thuringiaca* | 0 | 0 | 1 | 0 | 0 | 0 | 0 | 0 | 0 | 0 |
| *Carex sprengelii* | *Hymenochlaenae* | 0 | 0 | 1 | 0 | 0 | 0 | 0 | 0 | 0 | 0 |
| *Carex squarrosa* | *Squarrosae* | 0 | 0 | 1 | 0 | 0 | 0 | 0 | 0 | 0 | 0 |
| *Carex stellata* | *Schiedeanae* | 0 | 0 | 1 | 0 | 0 | 0 | 0 | 0 | 0 | 0 |
| *Carex stenocarpa* | *Aulocystis* | 0 | 1 | 0 | 0 | 0 | 0 | 0 | 1 | 0 | 0 |
| *Carex stenophylla* | *Divisae* | 1 | 1 | 0 | 1 | 0 | 0 | 0 | 1 | 0 | 0 |
| *Carex stenoptila* | *Ovales* | 0 | 0 | 1 | 0 | 0 | 0 | 0 | 0 | 0 | 0 |
| *Carex sterilis* | *Stellulatae* | 0 | 0 | 1 | 0 | 0 | 0 | 0 | 0 | 0 | 0 |
| *Carex steudneri* | *Indicae* | 0 | 0 | 0 | 1 | 0 | 0 | 0 | 0 | 0 | 0 |
| *Carex stevenii* | *Racemosae* | 0 | 0 | 1 | 0 | 0 | 0 | 0 | 0 | 0 | 0 |
| *Carex stipata* | *Vulpinae* | 0 | 1 | 1 | 0 | 0 | 0 | 0 | 0 | 0 | 0 |
| *Carex straminea* | *Ovales* | 0 | 0 | 1 | 0 | 0 | 0 | 0 | 0 | 0 | 0 |
| *Carex straminiformis* | *Ovales* | 0 | 0 | 1 | 0 | 0 | 0 | 0 | 0 | 0 | 0 |
| *Carex striata* | *Paludosae* | 0 | 0 | 1 | 0 | 0 | 0 | 0 | 0 | 0 | 0 |
| *Carex striatula* | *Laxiflorae* | 0 | 0 | 1 | 0 | 0 | 0 | 0 | 0 | 0 | 0 |
| *Carex stricta* | *Phacocystis* | 0 | 0 | 1 | 0 | 0 | 0 | 0 | 0 | 0 | 0 |
| *Carex strictissima* | *Uncinia* | 0 | 0 | 0 | 0 | 0 | 1 | 0 | 0 | 0 | 0 |
| *Carex strigosa* | *Sylvaticae* | 1 | 1 | 0 | 0 | 0 | 0 | 0 | 0 | 0 | 0 |
| *Carex styloflexa* | *Laxiflorae* | 0 | 0 | 1 | 0 | 0 | 0 | 0 | 0 | 0 | 0 |
| *Carex stylosa* | *Stylosae* | 1 | 1 | 1 | 0 | 0 | 0 | 0 | 0 | 0 | 0 |
| *Carex subbracteata* | *Ovales* | 0 | 0 | 1 | 0 | 0 | 0 | 0 | 0 | 0 | 0 |
| *Carex subebracteata* | *Mitratae* | 0 | 1 | 0 | 0 | 0 | 0 | 0 | 0 | 0 | 0 |
| *Carex suberecta* | *Ovales* | 0 | 0 | 1 | 0 | 0 | 0 | 0 | 0 | 0 | 0 |
| *Carex subfusca* | *Ovales* | 0 | 0 | 1 | 0 | 0 | 0 | 0 | 0 | 0 | 1 |
| *Carex subnigricans* | *Inflatae* | 0 | 0 | 1 | 0 | 0 | 0 | 0 | 0 | 0 | 0 |
| *Carex subphysodes* | *Physodeae* | 0 | 1 | 0 | 0 | 0 | 0 | 0 | 0 | 0 | 0 |
| *Carex subsacculata* | *Uncinia* | 0 | 0 | 0 | 0 | 1 | 0 | 0 | 0 | 0 | 0 |
| *Carex subspathacea* | *Phacocystis* | 1 | 1 | 1 | 0 | 0 | 0 | 0 | 0 | 0 | 0 |
| *Carex subtilis* | *Uncinia* | 0 | 0 | 0 | 0 | 0 | 1 | 0 | 0 | 0 | 0 |
| *Carex subviridis* | *Uncinia* | 0 | 0 | 0 | 0 | 0 | 1 | 0 | 0 | 0 | 0 |
| *Carex suifunensis* | *Phacocystis* | 0 | 1 | 0 | 0 | 0 | 0 | 0 | 0 | 0 | 0 |
| *Carex superata* | *Phyllostachyae* | 0 | 0 | 1 | 0 | 0 | 0 | 0 | 0 | 0 | 0 |
| *Carex swanii* | *Porocystis* | 0 | 0 | 1 | 0 | 0 | 0 | 0 | 0 | 0 | 0 |
| *Carex sychnocephala* | *Ovales* | 0 | 0 | 1 | 0 | 0 | 0 | 0 | 0 | 0 | 0 |
| *Carex sylvatica* | *Sylvaticae* | 1 | 1 | 0 | 0 | 0 | 0 | 0 | 0 | 0 | 0 |
| *Carex tahoensis* | *Ovales* | 0 | 0 | 1 | 0 | 0 | 0 | 0 | 0 | 0 | 0 |
| *Carex tangulashanensis* | *Lamprochlaenae* | 0 | 1 | 0 | 0 | 0 | 0 | 0 | 1 | 0 | 0 |
| *Carex tapintzensis* | *Clandestinae* | 0 | 0 | 0 | 0 | 0 | 0 | 0 | 1 | 0 | 0 |
| *Carex tasmanica* | *Echinochlaenae* | 0 | 0 | 0 | 0 | 0 | 1 | 0 | 0 | 0 | 0 |
| *Carex tenax* | *Hallerianae* | 0 | 0 | 1 | 0 | 0 | 0 | 0 | 0 | 0 | 0 |
| *Carex tenera* | *Ovales* | 0 | 0 | 1 | 0 | 0 | 0 | 0 | 0 | 0 | 0 |
| *Carex teneriformis* | *Ovales* | 0 | 0 | 1 | 0 | 0 | 0 | 0 | 0 | 0 | 1 |
| *Carex tenuiflora* | *Glareosae* | 1 | 1 | 1 | 0 | 0 | 0 | 0 | 0 | 0 | 0 |
| *Carex tetanica* | *Paniceae* | 0 | 0 | 1 | 0 | 0 | 0 | 0 | 0 | 0 | 0 |
| *Carex tetrastachya* | *Ovales* | 0 | 0 | 1 | 0 | 0 | 0 | 0 | 0 | 0 | 0 |
| *Carex texensis* | *Phaestoglochin* | 0 | 0 | 1 | 0 | 0 | 0 | 0 | 0 | 0 | 0 |
| *Carex thornei* | *Griseae* | 0 | 0 | 1 | 0 | 0 | 0 | 0 | 0 | 0 | 0 |
| *Carex thurberi* | *Vesicariae* | 0 | 0 | 1 | 0 | 1 | 0 | 0 | 0 | 0 | 0 |
| *Carex tibetikobresia* | *Kobresia* | 0 | 1 | 0 | 0 | 0 | 0 | 0 | 1 | 0 | 0 |
| *Carex timida* | *Phyllostachyae* | 0 | 0 | 1 | 0 | 0 | 0 | 0 | 0 | 0 | 0 |
| *Carex tincta* | *Ovales* | 0 | 0 | 1 | 0 | 0 | 0 | 0 | 0 | 0 | 0 |
| *Carex tomentosa* | *Acrocystis* | 1 | 1 | 0 | 0 | 0 | 0 | 0 | 0 | 0 | 0 |
| *Carex tonsa* | *Acrocystis* | 0 | 0 | 1 | 0 | 0 | 0 | 0 | 0 | 0 | 0 |
| *Carex torreyi* | *Porocystis* | 0 | 0 | 1 | 0 | 0 | 0 | 0 | 0 | 0 | 0 |
| *Carex torta* | *Phacocystis* | 0 | 0 | 1 | 0 | 0 | 0 | 0 | 0 | 0 | 0 |
| *Carex trachycarpa* | *Inversae* | 0 | 0 | 0 | 0 | 0 | 1 | 0 | 0 | 0 | 0 |
| *Carex traiziscana* | *Glareosae* | 0 | 1 | 0 | 0 | 0 | 0 | 0 | 0 | 0 | 0 |
| *Carex transandina* | *Aciculares* | 0 | 0 | 0 | 0 | 1 | 0 | 0 | 0 | 0 | 0 |
| *Carex transcaucasica* | *Phacocystis* | 1 | 1 | 0 | 0 | 0 | 0 | 0 | 0 | 0 | 0 |
| *Carex traversii* | *Echinochlaenae* | 0 | 0 | 0 | 0 | 0 | 1 | 0 | 0 | 0 | 0 |
| *Carex triangula* | *Uncinia* | 0 | 0 | 0 | 0 | 1 | 0 | 0 | 0 | 0 | 0 |
| *Carex triangularis* | *Multiflorae* | 0 | 0 | 1 | 0 | 0 | 0 | 0 | 0 | 0 | 0 |
| *Carex trichocarpa* | *Carex* | 0 | 0 | 1 | 0 | 0 | 0 | 0 | 0 | 0 | 0 |
| *Carex tricolor* | *Acrocystis* | 1 | 0 | 0 | 0 | 0 | 0 | 0 | 0 | 0 | 0 |
| *Carex trinervis* | *Phacocystis* | 1 | 0 | 0 | 0 | 0 | 0 | 0 | 0 | 0 | 0 |
| *Carex triquetra* | *Triquetrae* | 0 | 0 | 1 | 0 | 0 | 0 | 0 | 0 | 0 | 0 |
| *Carex trisperma* | *Glareosae* | 0 | 0 | 1 | 0 | 0 | 0 | 0 | 0 | 0 | 0 |
| *Carex tristachya* | *Mitratae* | 0 | 1 | 0 | 0 | 0 | 1 | 1 | 0 | 0 | 0 |
| *Carex tristis* | *Aulocystis* | 0 | 1 | 0 | 0 | 0 | 0 | 0 | 0 | 0 | 0 |
| *Carex troodi* | *Spirostachyae* | 1 | 0 | 0 | 0 | 0 | 0 | 0 | 0 | 0 | 0 |
| *Carex tsushimensis* | *Mitratae* | 0 | 1 | 0 | 0 | 0 | 0 | 0 | 0 | 0 | 0 |
| *Carex tuberculata* | *Fecundae* | 0 | 0 | 1 | 0 | 0 | 0 | 0 | 0 | 0 | 0 |
| *Carex tuckermanii* | *Vesicariae* | 0 | 0 | 1 | 0 | 0 | 0 | 0 | 0 | 0 | 0 |
| *Carex tumulicola* | *Phaestoglochin* | 0 | 0 | 1 | 0 | 0 | 0 | 0 | 0 | 0 | 0 |
| *Carex tunimanensis* | *Hymenochlaenae* | 0 | 0 | 1 | 0 | 1 | 0 | 0 | 0 | 0 | 0 |
| *Carex turbinata* | *Acrocystis* | 0 | 0 | 1 | 0 | 0 | 0 | 0 | 0 | 0 | 0 |
| *Carex turgescens* | *Rostrales* | 0 | 0 | 1 | 0 | 0 | 0 | 0 | 0 | 0 | 0 |
| *Carex turkestanica* | *Lamprochlaenae* | 0 | 1 | 0 | 0 | 0 | 0 | 0 | 1 | 0 | 0 |
| *Carex typhina* | *Squarrosae* | 0 | 0 | 1 | 0 | 0 | 0 | 0 | 0 | 0 | 0 |
| *Carex uhligii* | *Schoenoxiphium* | 0 | 0 | 0 | 1 | 0 | 0 | 0 | 0 | 0 | 0 |
| *Carex ulobasis* | *Acrocystis* | 0 | 1 | 0 | 0 | 0 | 0 | 0 | 0 | 0 | 0 |
| *Carex umbellata* | *Acrocystis* | 0 | 0 | 1 | 0 | 0 | 0 | 0 | 0 | 0 | 0 |
| *Carex umbricola* | *Uncinia* | 0 | 0 | 0 | 0 | 0 | 1 | 0 | 0 | 0 | 0 |
| *Carex umbrosa* | *Mitratae* | 1 | 1 | 0 | 0 | 0 | 0 | 0 | 0 | 0 | 0 |
| *Carex uncinata* | *Uncinia* | 0 | 0 | 0 | 0 | 0 | 1 | 0 | 0 | 0 | 1 |
| *Carex uncinioides* | *Kobresia* | 0 | 1 | 0 | 0 | 0 | 0 | 1 | 1 | 0 | 0 |
| *Carex unilateralis* | *Ovales* | 0 | 0 | 1 | 0 | 0 | 0 | 0 | 0 | 0 | 0 |
| *Carex ursina* | *Glareosae* | 1 | 1 | 1 | 0 | 0 | 0 | 0 | 0 | 0 | 0 |
| *Carex ussuriensis* | *Albae* | 0 | 1 | 0 | 0 | 0 | 0 | 0 | 0 | 0 | 0 |
| *Carex utriculata* | *Vesicariae* | 1 | 1 | 1 | 0 | 0 | 0 | 0 | 0 | 0 | 0 |
| *Carex vacillans* | *Phacocystis* | 1 | 0 | 1 | 0 | 0 | 0 | 0 | 0 | 0 | 0 |
| *Carex vaginosa* | *Kobresia* | 0 | 0 | 0 | 0 | 0 | 0 | 0 | 1 | 0 | 0 |
| *Carex valbrayi* | *Indicae* | 0 | 0 | 0 | 1 | 0 | 0 | 0 | 0 | 0 | 0 |
| *Carex vallicola* | *Phaestoglochin* | 0 | 0 | 1 | 0 | 0 | 0 | 0 | 0 | 0 | 0 |
| *Carex vallispulchrae* | *Aciculares* | 0 | 0 | 0 | 0 | 1 | 0 | 0 | 0 | 1 | 0 |
| *Carex vallisrosetto* | *Spirostachyae* | 0 | 0 | 0 | 1 | 0 | 0 | 0 | 0 | 0 | 0 |
| *Carex venusta* | *Hymenochlaenae* | 0 | 0 | 1 | 0 | 0 | 0 | 0 | 0 | 0 | 0 |
| *Carex vernacula* | *Foetidae* | 0 | 0 | 1 | 0 | 0 | 0 | 0 | 0 | 0 | 0 |
| *Carex verrucosa* | *Glaucescentes* | 0 | 0 | 1 | 0 | 0 | 0 | 0 | 0 | 0 | 0 |
| *Carex vesicaria* | *Vesicariae* | 1 | 1 | 1 | 0 | 0 | 0 | 0 | 0 | 0 | 0 |
| *Carex vestita* | *Paludosae* | 0 | 0 | 1 | 0 | 0 | 0 | 0 | 0 | 0 | 0 |
| *Carex vexans* | *Ovales* | 0 | 0 | 1 | 0 | 0 | 0 | 0 | 0 | 0 | 0 |
| *Carex vidua* | *Kobresia* | 0 | 1 | 0 | 0 | 0 | 0 | 0 | 1 | 0 | 0 |
| *Carex virescens* | *Porocystis* | 0 | 0 | 1 | 0 | 0 | 0 | 0 | 0 | 0 | 0 |
| *Carex viridistellata* | *Ceratocystis* | 0 | 0 | 1 | 0 | 0 | 0 | 0 | 0 | 0 | 0 |
| *Carex viridula* | *Ceratocystis* | 0 | 1 | 0 | 0 | 0 | 0 | 0 | 0 | 0 | 0 |
| *Carex vixdentata* | *Spirostachyae* | 0 | 0 | 0 | 0 | 1 | 0 | 0 | 0 | 0 | 0 |
| *Carex vulpinaris* | *Phaestoglochin* | 0 | 0 | 0 | 0 | 0 | 0 | 0 | 1 | 0 | 0 |
| *Carex vulpinoidea* | *Multiflorae* | 0 | 0 | 1 | 0 | 1 | 0 | 0 | 0 | 0 | 0 |
| *Carex wahlenbergiana* | *Indicae* | 0 | 0 | 0 | 1 | 0 | 0 | 0 | 0 | 0 | 0 |
| *Carex wallichiana* | *Echinochloomorphae* | 0 | 0 | 0 | 0 | 0 | 0 | 0 | 1 | 0 | 0 |
| *Carex whitneyi* | *Longicaules* | 0 | 0 | 1 | 0 | 0 | 0 | 0 | 0 | 0 | 0 |
| *Carex wiegandii* | *Stellulatae* | 0 | 0 | 1 | 0 | 0 | 0 | 0 | 0 | 0 | 0 |
| *Carex willdenowii* | *Phyllostachyae* | 0 | 0 | 1 | 0 | 0 | 0 | 0 | 0 | 0 | 0 |
| *Carex williamsii* | *Chlorostachyae* | 1 | 1 | 1 | 0 | 0 | 0 | 0 | 0 | 0 | 0 |
| *Carex woodii* | *Paniceae* | 0 | 0 | 1 | 0 | 0 | 0 | 0 | 0 | 0 | 0 |
| *Carex wootonii* | *Ovales* | 0 | 0 | 1 | 0 | 0 | 0 | 0 | 0 | 0 | 0 |
| *Carex xalapensis* | *Phaestoglochin* | 0 | 0 | 1 | 0 | 1 | 0 | 0 | 0 | 0 | 0 |
| *Carex xerantica* | *Ovales* | 0 | 0 | 1 | 0 | 0 | 0 | 0 | 0 | 0 | 0 |
| *Carex yadongensis* | *Kobresia* | 0 | 0 | 0 | 0 | 0 | 0 | 0 | 1 | 0 | 0 |
| *Carex yandangshanica* | *Rhomboidales* | 0 | 1 | 0 | 0 | 0 | 0 | 0 | 0 | 0 | 0 |
| *Carex zikae* | *Acrocystis* | 0 | 0 | 1 | 0 | 0 | 0 | 0 | 0 | 0 | 0 |
| *Carex zotovii* | *Uncinia* | 0 | 0 | 0 | 0 | 0 | 1 | 0 | 0 | 0 | 0 |
| *Carex zuluensis* | *Indicae* | 0 | 0 | 0 | 1 | 0 | 0 | 0 | 0 | 0 | 0 |
