# Supplementary material for: Timing and ecological priority shaped the diversification of sedges in the Himalayas
Source: PeerJ. 2019 Jun 7;7:e6792. doi: 10.7717/peerj.6792 (PMC6557248; doi:10.7717/peerj.6792)
Supplement: Table S5 [file peerj-07-6792-s011.docx]

**Table S5 List of species with synonym names according to Global *Carex* Group (2015) were also used in order to retrieve GBIF data**.

| **Species** | **Synonym names for the species** |
| --- | --- |
| *Carex capillifolia* | *Kobresia capillifolia* |
| *Carex cercostachys* | *Kobresia cercostachys* |
| *Carex coninux* | *Kobresia karakorumensis* |
| *Carex curticeps* | *Kobresia curticeps* |
| *Carex deasyi* | *Kobresia schoenoides* |
| *Carex esenbeckii* | *Kobresia esenbeckii* |
| *Carex filispica* | *Kobresia filicina* |
| *Carex fissiglumis* | *Kobresia fissiglumis* |
| *Carex gammiei* | *Kobresia gammiei* |
| *Carex setschwanensis* | *Kobresia setschwanensis* |
| *Carex simpliciuscula* | *Kobresia simpliciuscula* |
| *Carex sanguinea* | *Kobresia sanguinea* |
| *Carex bhutanensis* | *Kobresia prainii* |
| *Carex bistaminata* | *Kobresia myosuroides* |
| *Carex borealipolaris* | *Kobresia sibirica* |
| *Carex breviprophylla* | *Kobresia gandakiensis* |
| *Carex handel-mazzettii* | *Kobresia handel-mazzettii* |
| *Carex prainii* | *Kobresia sikkimensis* |
| *Carex harae* | *Kobresia harae* |
| *Carex pseuduncinoides* | *Kobresia kansuensis* |
| *Carex parvula* | *Kobresia pygmaea* |
| *Carex pseudogammiei* | *Kobresia loliacea* |
| *Carex pseudolaxa* | *Kobresia laxa* |
| *Carex hughii* | *Kobresia graminifolia* |
| *Carex vidua* | *Kobresia vidua* |
| *Carex uncinioides* | *Kobresia uncinioides* |
| *Carex alatauensis* | *Kobresia humilis* |
| *Carex bistaminata* | *Kobresia myosuroides* |
| *Carex peichuniana* | *Kobresia inflata* |
| *Carex vaginosa* | *Kobresia nepalensis* |
| *Carex littledalei* | *Kobresia littledalei* |
| *Carex ovoidispica* | *Kobresia nitens* |
| *Carex parvispica* | *Uncinia sinclairii* |
| *Carex phleoides* | *Uncinia phleoides* |
| *Carex astricta* | *Uncinia caespitosa* |
| *Carex zotovii* | *Uncinia zotovii* |
| *Carex salticola* | *Uncinia andina* |
| *Carex perplexa* | *Uncinia perplexa* |
| *Carex punicea* | *Uncinia rubra* |
| *Carex triangula* | *Uncinia triquetra* |
| *Carex penalpina* | *Uncinia purpurata* |
| *Carex corynoidea* | *Uncinia clavata* |
| *Carex potens* | *Uncinia affinis* |
| *Carex crispa* | *Uncinia involuta* |
| *Carex cyanea* | *Uncinia leptostachya* |
| *Carex drucei* | *Uncinia drucei* |
| *Carex ecuadorensis* | *Uncinia ecuadorensis* |
| *Carex multifaria* | *Uncinia multifaria* |
| *Carex edura* | *Uncinia divaricata* |
| *Carex egmontiana* | *Uncinia egmontiana* |
| *Carex erebus* | *Uncinia hookeri* |
| *Carex erinacea* | *Uncinia erinacea* |
| *Carex obtusifolia* | *Uncinia obtusifolia* |
| *Carex erythrovaginata* | *Uncinia laxiflora* |
| *Carex firmula* | *Uncinia tenuis* |
| *Carex hamata* | *Uncinia hamata* |
| *Carex longifructus* | *Uncinia longifructus* |
| *Carex minor* | *Uncinia angustifolia* |
| *Carex potens* | *Uncinia riparia* |
| *Carex subtilis* | *Uncinia macrolepis* |
| *Carex uncinata* | *Uncinia uncinata* |
| *Carex megalepis* | *Uncinia ferruginea* |
| *Carex subsacculata* | *Uncinia subsacculata* |
| *Carex madida* | *Uncinia lacustris* |
| *Carex subtilis* | *Uncinia elegans* |
| *Carex horizontalis* | *Uncinia rupestris* |
| *Carex imbecilla* | *Uncinia gracilenta* |
| *Carex fraseriana* | *Cymophyllus fraserianus* |
| *Carex killickii* | *Schoenoxiphium filiforme* |
| *Carex uhligii* | *Schoenoxiphium lehmannii* |
| *Carex lancea* | *Schoenoxiphium lanceum* |
| *Carex ludwigii* | *Schoenoxiphium ludwigii* |
| *Carex pseudorufa* | *Schoenoxiphium burttii* |
| *Carex spartea* | *Schoenoxiphium sparteum* |
| *Carex schweickerdtii* | *Schoenoxiphium schweickerdtii* |
| *Carex spartea* | *Schoenoxiphium sparteum* |
| *Carex basutorum* | *Schoenoxiphium basutorum* |
