# Supplementary material for: Timing and ecological priority shaped the diversification of sedges in the Himalayas
Source: PeerJ. 2019 Jun 7;7:e6792. doi: 10.7717/peerj.6792 (PMC6557248; doi:10.7717/peerj.6792)
Supplement: Table S6 — xxx= sequence was not available. *only Morton Arboretum herbarium voucher numbers were mentioned here as complete copy of specimens was submitted to this herbarium, while for last 27 specimens, herbarium voucher number was mentioned from where these were received. [file peerj-07-6792-s012.docx]

**Table S6 List of new sequences with their GenBank accession numbers for ETS, ITS and matK regions**

| **Species** | **Herbarium vouchers*** | **GenBank Accession Numbers** | | |  |
| --- | --- | --- | --- | --- | --- |
|  |  | **ETS** | **ITS** | **matK** | |
| *Carex atrofusca* | 183078 | MH191326 | MH329389 | xxx | |
| *Carex atrofusca* | 183077 | xxx | xxx | xxx | |
| *Carex brunnea* | 183095 | MH191327 | MH329390 | MH496659 | |
| *Carex brunnea* | 183093 | MH191328 | MH329391 | MH496660 | |
| *Carex brunnea* | 183087 | MH208841 | MH329392 | MH496662 | |
| *Carex brunnea* | 183089 | MH208842 | MH329393 | MH496663 | |
| *Carex brunnea* | 183090 | MH208843 | MH329394 | MH496664 | |
| *Carex brunnea* | 183056 | xxx | xxx | xxx | |
| *Carex brunnea* | 183094 | MH208844 | MH329395 | MH496665 | |
| *Carex brunnea* | 183088 | MH208845 | xxx | MH496666 | |
| *Carex brunnea* | 183096 | MH208846 | MH329396 | MH496667 | |
| *Carex brunnea* | 183092 | MH208847 | MH329397 | MH496668 | |
| *Carex brunnea* | 183091 | MH208848 | MH348210 | MH496669 | |
| *Carex canescens* | 183104 | MH208850 | MH348211 | MH496670 | |
| *Carex canescens* | 183103 | MH208851 | MH348212 | MH496671 | |
| *Carex cardiolepis* | 183054 | MH208852 | MH348214 | xxx | |
| *Carex filicina* | 183074 | MH219919 | MH348216 | MH496674 | |
| *Carex filicina* | 183073 | MH219920 | MH348217 | MH496675 | |
| *Carex filicina* | 183065 | MH219921 | MH348218 | xxx | |
| *Carex fedia* | 183064 | MH219922 | MH348215 | MH496673 | |
| *Carex infuscata* | 183057 | xxx | xxx | xxx | |
| *Carex infuscata* | 183079 | MH219923 | MH348219 | MH496676 | |
| *Carex infuscata* | 183080 | MH219924 | MH348220 | xxx | |
| *Carex infuscata* | 183058 | xxx | xxx | xxx | |
| *Carex nubigena* | 183082 | MH219925 | MH348221 | MH496677 | |
| *Carex nubigena* | 183085 | MH219926 | MH348222 | MH496678 | |
| *Carex nubigena* | 183086 | MH229870 | MH348223 | MH496679 | |
| *Carex nubigena* | 183081 | MH229871 | MH348224 | MH496680 | |
| *Carex nubigena* | 183084 | MH229872 | MH348225 | MH496681 | |
| *Carex nubigena* | 183083 | MH229873 | MH348226 | MH496682 | |
| *Carex pamirica* | 183076 | xxx | xxx | xxx | |
| *Carex pseudofoetida* | 183071 | xxx | MH348227 | MH496683 | |
| *Carex pseudofoetida* | 183072 | xxx | MH348228 | MH496684 | |
| *Carex pseudolaxa* | 183109 | MH229874 | MH348229 | MH496685 | |
| *Carex pseudolaxa* | 183107 | MH229875 | MH348230 | MH496686 | |
| *Carex pseudolaxa* | 183106 | MH229876 | MH348231 | MH496687 | |
| *Carex pseudolaxa* | 183108 | MH229877 | MH348232 | MH496688 | |
| *Carex pseudolaxa* | 183112 | MH229878 | MH348233 | MH496689 | |
| *Carex pseudolaxa* | 183111 | MH229879 | MH348234 | MH496690 | |
| *Carex pseudolaxa* | 183110 | MH229880 | MH348235 | MH496691 | |
| *Carex pseudolaxa* | 183105 | MH229881 | MH348236 | xxx | |
| *Carex psychrophila* | 183055 | xxx | xxx | xxx | |
| *Carex sanguinea* | 183075 | MH229882 | MH348237 | MH496692 | |
| *Carex schlagintweitiana* | 183068 | MH229883 | xxx | MH496693 | |
| *Carex wallichiana* | 183066 | MH229884 | MH348241 | MH496695 | |
| *Carex wallichiana* | 183067 | MH229885 | MH348242 | MH496696 | |
| *Carex dimorpholepis* | 184272 | xxx | xxx | xxx | |
| *Carex brunnea* | 184278 | MH208849 | xxx | MH496661 | |
| *Carex diandra* | 184275 | MH208853 | xxx | xxx | |
| *Carex schlagintweitiana* | 184274; 184273 | MH329370 | MH348238 | MH496694 | |
| *Carex cardiolepis* | 184271 | MH329369 | MH348213 | MH496672 | |
| *Carex pseudocyperus* | 184276 | xxx | xxx | xxx | |
| *Carex pamirica* | 184270 | xxx | xxx | xxx | |
| *Carex simpliciuscula* | 183069 | MH329371 | MH348239 | xxx | |
| *Carex simpliciuscula* | 183070 | MH329372 | MH348240 | xxx | |
| *Carex breviprophylla* | E00184752 | MH329381 | xxx | MH496708 | |
| *Carex daltonii* | E00047630 | MH329373 | MH348243 | MH496697 | |
| *Carex finitima* | E00269484 | MH329374 | MH348244 | MH496698 | |
| *Carex fissiglumis* | E00693544 | MH329380 | xxx | MH496707 | |
| *Carex fucata* | 5778634 | MH329388 | xxx | xxx | |
| *Carex fusiformis* | E00666305 | xxx | MH348245 | MH496699 | |
| *Carex gentilis* | 5306057 | MH329387 | xxx | xxx | |
| *Carex harae* | E00048401 | MH329383 | xxx | xxx | |
| *Carex harae* | E00048402 | MH329382 | xxx | MH496709 | |
| *Carex jackiana* | E00666333 | MH329375 | MH348246 | MH496700 | |
| *Carex kumaonensis* | E00656734 | MH329376 | MH348247 | MH496701 | |
| *Carex longicruris* | E00693600 | MH329377 | xxx | MH496702 | |
| *Carex longicruris* | E00693633 | xxx | xxx | MH496703 | |
| *Carex longipes* | E00693601 | MH329378 | MH348248 | MH496704 | |
| *Carex longipes* | E00003298 | xxx | MH348249 | MH496705 | |
| *Carex obovatosquamata* | E00666339 | MH329379 | xxx | MH496706 | |
| *Carex obscuriceps* | E00693599 | xxx | MH348250 | xxx | |
| *Carex ovatispiculata* | MSB-140867 | MH329386 | xxx | MH496712 | |
| *Carex prainii* | E00256786 | MH329384 | xxx | MH496710 | |
| *Carex pruinosa* | E00263657 | xxx | MH348251 | xxx | |
| *Carex pruinosa* | E00264126 | xxx | MH348252 | xxx | |
| *Carex pulchra* | E00693608 | xxx | MH348253 | xxx | |
| *Carex radicalis* | E00693596 | xxx | MH348254 | xxx | |
| *Carex rufulistolon* | E00424592 | xxx | MH348255 | xxx | |
| *Carex rufulistolon* | E00270126 | xxx | MH348256 | xxx | |
| *Carex rufulistolon* | E00238910 | xxx | MH348257 | xxx | |
| *Carex yadongensis* | E00177570 | MH329385 | xxx | MH496711 | |

**xxx= sequence was not available**

*****only Morton Arboretum herbarium voucher numbers were mentioned here as complete copy of specimens was submitted to this herbarium, while for last 27 specimens, herbarium voucher number was mentioned from where these were received.
