# Supplementary material for: Timing and ecological priority shaped the diversification of sedges in the Himalayas
Source: PeerJ. 2019 Jun 7;7:e6792. doi: 10.7717/peerj.6792 (PMC6557248; doi:10.7717/peerj.6792)
Supplement: Table S7 — The average of all the dispersal events with standard deviations in anagenetic events. Here, W, Western Palearctic, E, Eastern Palearctic, N, Nearctic, A, Afrotropic, T, Neotropic, U, Australasia, I, Indo-Malaya, H, Himalaya, C, Antarctic and O, Oceania [file peerj-07-6792-s013.docx]

**Table S7 Summary of the anagenetic and cladogenetic events obtained from 1000 BSM.** The average of all the dispersal events with standard deviations in anagenetic events. Here, W = Western Palearctic, E = Eastern Palearctic, N = Nearctic, A = Afrotropic, T = Neotropic, U = Australasia, I = Indo-Malaya, H = Himalaya, C = Antarctic and O = Oceania

|  | **Vicariance events** | **Sympatric events** | **Dispersal events** | **All anagenetic events** | **All cladogenetic events** | **Total events** |
| --- | --- | --- | --- | --- | --- | --- |
| **Means** | 52.96 | 910.5 | 734.5 | 734.5 | 965 | 1709 |
| **Standard deviation** | 5.96 | 10.4 | 17.85 | 15.1 | 0 | 15.1 |

**Anagenetic dispersal or all dispersal events (mean and standard deviation of all observed anagenetic dispersals)**

**Means:**

|  | **W** | **E** | **N** | **A** | **T** | **U** | **I** | **H** |
| --- | --- | --- | --- | --- | --- | --- | --- | --- |
| **W** | 0 | 47.91 | 21.39 | 14.46 | 9.67 | 8.18 | 5.04 | **14.48** |
| **E** | 63.56 | 0 | 42.06 | 12.68 | 10.23 | 15.04 | 21.32 | **39.42** |
| **N** | 48.54 | 63.72 | 0 | 15.79 | 44.53 | 13.61 | 10.6 | **21.44** |
| **A** | 4.04 | 4.62 | 5.47 | 0 | 3.77 | 2.58 | 2.54 | **4.12** |
| **T** | 5.68 | 5.12 | 8.27 | 3.06 | 0 | 4.68 | 2.56 | **3.60** |
| **U** | 3.73 | 5.15 | 3.25 | 2.52 | 2.48 | 0 | 3.69 | **3.64** |
| **I** | 2.5 | 6.04 | 2 | 1.81 | 2.06 | 3.47 | 0 | **5.22** |
| **H** | **12.48** | **35.26** | **8.42** | **6.52** | **5.52** | **7.74** | **12.88** | **0** |

**Standard deviations:**

|  | **W** | **E** | **N** | **A** | **T** | **U** | **I** | **H** |
| --- | --- | --- | --- | --- | --- | --- | --- | --- |
| **W** | 0 | 5.8 | 4.15 | 2.66 | 2.57 | 2.71 | 2.34 | **3.63** |
| **E** | 7.23 | 0 | 6.77 | 3.38 | 2.82 | 3.04 | 3.71 | **5.23** |
| **N** | 5.51 | 6.88 | 0 | 3.39 | 3.49 | 3.43 | 3.22 | **4.31** |
| **A** | 1.97 | 2.44 | 2.17 | 0 | 1.65 | 1.61 | 1.62 | **1.89** |
| **T** | 2.23 | 2.27 | 2.25 | 1.84 | 0 | 1.6 | 1.62 | **2.47** |
| **U** | 1.86 | 2.2 | 1.85 | 1.65 | 1.59 | 0 | 1.82 | **1.83** |
| **I** | 1.6 | 2.35 | 1.45 | 1.41 | 1.51 | 1.73 | 0 | **2.16** |
| **H** | **3.72** | **4.11** | **3.25** | **2.14** | **2.23** | **2.43** | **3.31** | **0** |
